# Safety, Tolerability, and Immunogenicity of the Novel Antituberculous Vaccine RUTI: Randomized, Placebo-Controlled Phase II Clinical Trial in Patients with Latent Tuberculosis Infection

**DOI:** 10.1371/journal.pone.0089612

**Published:** 2014-02-26

**Authors:** Andre S. Nell, Eva D’lom, Patrick Bouic, Montserrat Sabaté, Ramon Bosser, Jordi Picas, Mercè Amat, Gavin Churchyard, Pere-Joan Cardona

**Affiliations:** 1 PAREXEL Early Phase Clinical Unit, Bloemfontein, South Africa; 2 PAREXEL International, Madrid, Spain; 3 Synexa Life Sciences, Cape Town, South Africa; 4 TFS Develop, Barcelona, Catalonia, Spain; 5 Janus Developments, Barcelona, Catalonia, Spain; 6 Archivel Farma, Badalona, Catalonia, Spain; 7 The Aurum Institute, Johannesburg, South Africa; 8 Unitat de Tuberculosi Experimental (UTE), Institut per a la Investigació en Ciències de la Salut Germans Trias i Pujol, Universitat Autònoma de Barcelona, Badalona, Catalonia, Spain; Public Health England, United Kingdom

## Abstract

**Objectives:**

To evaluate the safety, tolerability and immunogenicity of three different doses (5, 25 and 50 µg) of the novel antituberculous vaccine RUTI compared to placebo in subjects with latent tuberculosis infection.

**Methods and Findings:**

Double-blind, randomized, placebo-controlled Phase II Clinical Trial (95 patients randomized). Three different RUTI doses and placebo were tested, randomized both in HIV-positive (n = 47) and HIV-negative subjects (n = 48), after completion of one month isoniazid (INH) pre-vaccination. Each subject received two vaccine administrations, 28 Days apart. Five patients withdrew and 90 patients completed the study. Assessment of safety showed no deaths during study. Two subjects had serious adverse events one had a retinal detachment while taking INH and was not randomized and the other had a severe local injection site abscess on each arm and was hospitalized; causality was assessed as very likely and by the end of the study the outcome had resolved. All the patients except 5 (21%) patients of the placebo group (3 HIV+ and 2 HIV−) reported at least one adverse event (AE) during the study. The most frequently occurring AEs among RUTI recipients were (% in HIV+/−): injection site reactions [erythema (91/92), induration (94/92), local nodules (46/25), local pain (66/75), sterile abscess (6/6), swelling (74/83), ulcer (20/11), headache (17/22) and nasopharyngitis (20/5)]. These events were mostly mild and well tolerated. Overall, a polyantigenic response was observed, which differed by HIV− status. The best polyantigenic response was obtained when administrating 25 µg RUTI, especially in HIV-positive subjects which was not increased after the second inoculation.

**Conclusion:**

This Phase II clinical trial demonstrates reasonable tolerability of RUTI. The immunogenicity profile of RUTI vaccine in LTBI subjects, even being variable among groups, allows us considering one single injection of one of the highest doses in future trials, preceded by an extended safety clinical phase.

**Trial Registration:**

ClinicalTrials.gov NCT01136161

## Introduction

Tuberculosis (TB) is caused by *Mycobacterium tuberculosis*, an intracellular microorganism that is still considered to be among the world’s most devastating pathogens. In 2011, there were 8.7 million incident cases of TB, almost 1 million deaths from TB among HIV-negative people and an additional 0.43 million deaths from HIV-associated TB [1]. One third of the world’s population is infected with latent TB, and at risk of developing the active disease. Latently infected *M. tuberculosis* individuals have the possibility to develop active TB throughout their lives. Progression to active TB is relatively low in HIV-negative subjects (from 5 to 25% of infected people), although even this low percentage represents 9 million new TB cases every year [2]. Moreover, in HIV infected subjects, reactivation of TB disease incidence can be 10 to 100 times higher [3]. Standard treatment for latent TB infection (LTBI) requires the administration of isoniazid (INH) from 6 to 9 months, which results in important compliance problems. Moreover, this treatment does not ensure the complete cure of the patient and it is not exempt of adverse effects [4]. Current knowledge of latent bacilli and of the lesions with which they are associated suggests that these bacilli survive inside granulomas. In particular within the central necrotic core and inside the neighbour layer of foamy macrophages (FM). Following its natural evolution, FM drain from the granuloma to alveolar spaces and release dormant bacilli into the airways that can reinfect healthy parenchyma. A new therapeutic vaccine, RUTI, was designed to generate a polyantigenic immune response that targets bacilli that persist after isoniazid preventive therapy and to activate naive macrophages that are phagocyting the necrotic tissue that contains them [5]–[7].

RUTI is a polyantigenic liposomal vaccine made of detoxified, fragmented *M. tuberculosis* cells (FCMtb), for the prevention of active TB in subjects with LTBI. It is developed in Badalona (Catalonia, Spain) by Archivel Farma. In 2007 a Phase I Clinical Trial (CT) was designed to test tolerability and immunogenicity of 4 increasing doses of the vaccine RUTI (5, 25, 100 and 200 µg of FCMb) administrated to healthy volunteers. The results showed that the vaccination was reasonably well tolerated as judged by local and systemic clinical evaluation, though dose dependent local adverse reactions were noted; and it trigged a specific immunological response against *M. tuberculosis* in healthy subjects, compared to placebo [8].

The present study is a double-blind, randomized, placebo-controlled Phase II clinical trial to assess the safety, tolerability, and immunogenicity of three doses of RUTI vaccine (5, 25 and 50 µg of FCMb) administered after completion of one month of isoniazid treatment in HIV-infected and –uninfected subjects with LTBI.

## Materials and Methods

The protocol for this trial and supporting CONSORT checklist are available as supporting information; see Checklist S1 and Protocol S1.

### Study Site

The study was carried out by three South African sites (Bloemfontein, George and Port Elizabeth) from July 2010 to April 2011 (recruitment from July 2010 to January 2011 and follow-up until April 2011).

All the objectives, methodology as well as possible inconveniences and risks due to the study were explained to each subject, orally and in writing (by a consent form) before their inclusion. All the subjects signed the consent form before starting any procedure.

### Study Design

This was a phase II, randomized placebo-controlled clinical trial to assess the safety, tolerability, and immunogenicity of three doses of RUTI vaccine and placebo (randomly assigned both in HIV-positive and HIV-negative subjects) administered after completion of one month treatment with INH (one tablet of 300 mg/day, per mouth). The INH treatment compliance was self-reported by the subjects through diary cards with the investigator’s oversight at each of the 3 visits during INH treatment period. Omission of 1 tablet/week was accepted.

**Figure 1:**
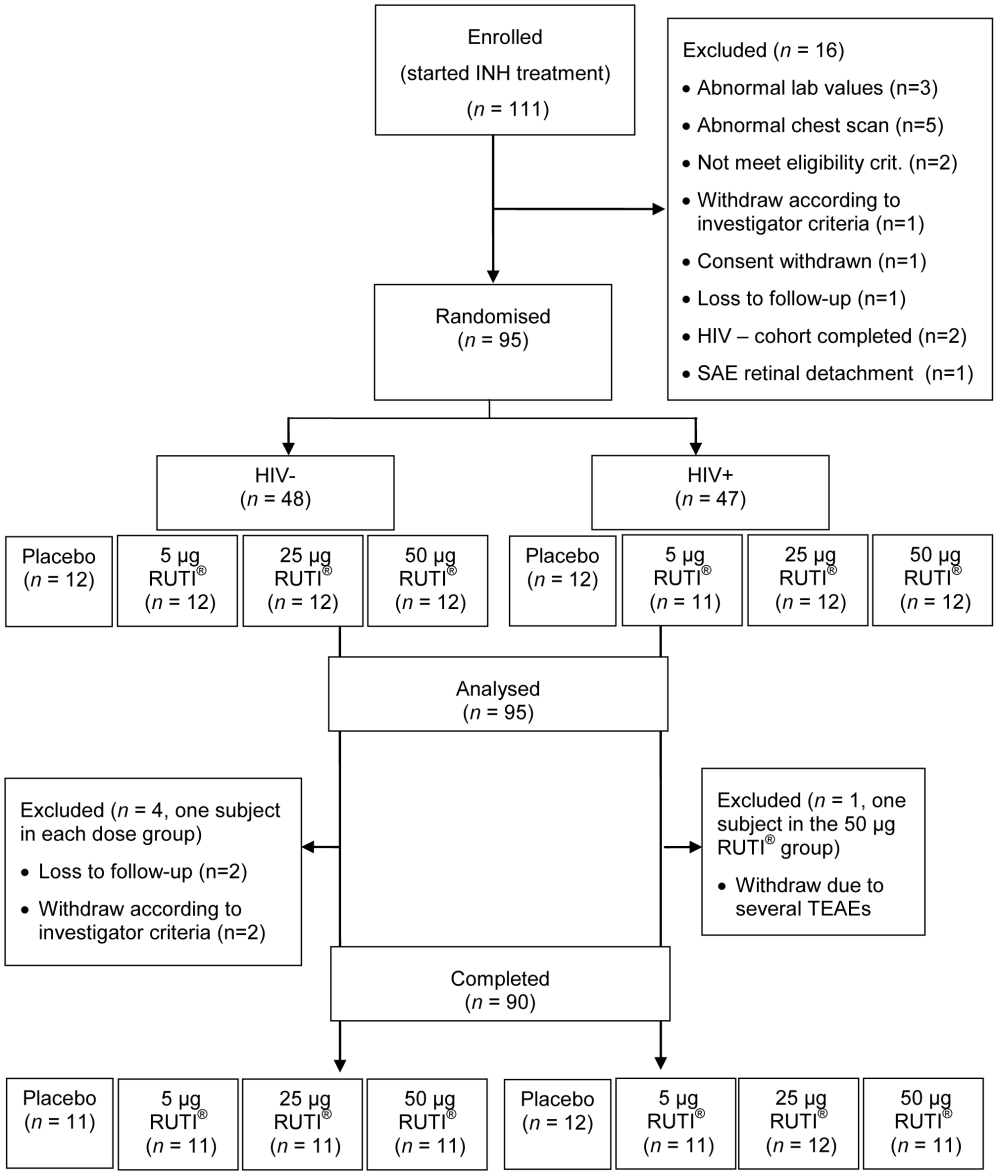
Consort chart representing the participant flow of the CT. From the 111 subjects included, ninety-five patients were randomised and included in both the safety and immunogenic analyses. Five patients withdrew the study, being a total of 90 patients who completed the trial.

**Figure 2:**
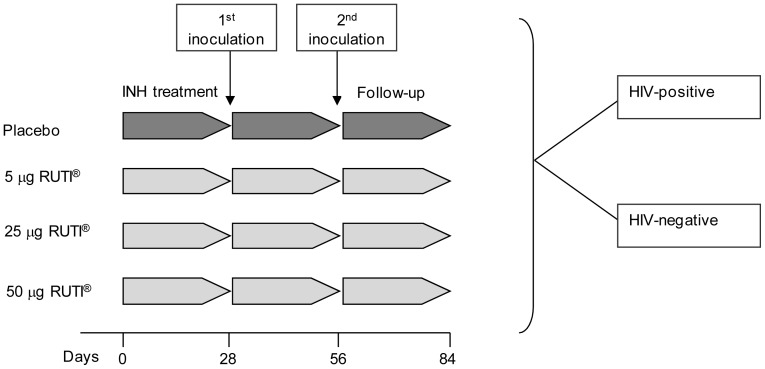
Study design of the clinical trial.

Each subject was randomized to receive one of the four treatments: placebo, 5, 25 or 50 µg (two subcutaneous administrations of 0.3 mL of the same treatment, at deltoid muscle area of alternate arms, 28 Days apart) [Fig. 1]. Each vial of placebo contents: sucrose (50,000.0 µg/mL), soy lecithin (2,114.4 µg/mL), sodium cholate 230.0 µg/mL and sodium chloride 52.1 µg/mL, which is exactly the same composition of RUTI vaccine, excepting the content of drug substance. (FCMtb). Subjects were monitored until one month after the second inoculation with RUTI (Fig. 2).

### Randomization and Blinding

The medication (RUTI vials) was prepared giving the RUTI or placebo after the random attribution to a serial number generated using the RANDPLAN, a locally developed SAS validated macro utilizing the PROC PLAN procedure in SAS. The randomization list was sent to the persons responsible of the medication storage and labelling and the rest of the personnel were kept blinded.

Medication was transferred to pharmacists at each trial site with the correspondent sealed envelope labelled with the serial number containing information on the nature of the medication inside. The envelopes were maintained in a locked secure place throughout the whole study while still allowing access for emergency code breaking only.

### Study Subjects

Study inclusion criteria were men and women 18–50 years of age, without evidence of active TB, positive tuberculin skin test (TST+) [≥5 mm induration] and Quantiferon TB Gold positive result. Additional inclusion criteria in the HIV-positive group were: HIV antibody positive, CD4 count ≥350 cells/mL and clinically stable subjects if on anti-retroviral treatment.

### Protocol Deviations

All deviations were considered minor, with an exception of one deviation that was considered major. The major deviation was recorded for one patient (HIV-negative group 5 µg RUTI) at Day 63, PBMC blood samples were delivered to the laboratory with a delay of more than 4 hours. Additional samples had to be collected next day.

One patient was included in the study while using fluoxetine, which was prohibited by the study protocol. This subject was withdrawn from the study prior to randomisation and was therefore not included in any of the study populations.

An un-contrasted thoracic computerised tomographic scan was to be performed prior to first vaccine inoculation and four weeks after the last one (2^nd^ vaccine inoculation) to evaluate the change in size of the hilar lymph nodes to discard pulmonary TB cases. Although computerised tomographic scan was performed to all subjects, the size of the hilar lymph nodes was not recorded.

Three patients took theophylline in the pre-treatment phase which was a prohibited concomitant medication according the exclusion criteria.

### Evaluation of Tolerability

Local tolerability at the site of injection was assessed for redness, pain, swelling, induration and functional limitation. Time points were Days 28 (post-1^st^ dose), 29, 31, 35, 49, 56 (pre and post-2^nd^ dose), 57, 59, 63 and 84. Pain intensity at the inoculation point was assessed subjectively by patients using a visual analogical scale (VAS) ranging from 0 (no pain) to 100 (maximum pain). The presence of abscess, ulceration or necrosis was also evaluated and measured.

Signs of hilar lymph node enlargement were assessed using an un-contrasted thoracic computerised high resolution tomographic scan prior to the first and four weeks after the last vaccination.

Systemic tolerability defined as: body temperature ≥38°C, asthenia, sweating, malaise, headache, dizziness, nausea, myalgia, arthralgia, rash and generalised pruritus was also assessed on the same days as the local reactions were assessed.

### Evaluation of Safety

The subjects were monitored by the study team in order to perform the following safety evaluations: (1) surveillance for local and systemic adverse events, for the duration of the study, detected through self-report and after questioning; (2) vital signs; (3) physical examination; (4) ECG and (5) laboratory safety tests. AEs detected by the investigator through interrogation or reported by the subject during the defined period of collection of AE were recorded. Adverse events were classified using the System Organ Class (SOC) of MedDRA. Participants with SAEs were followed-up until events resolved.

Safety monitoring of hematological and biochemical parameters were measured at screening at days 21, 28, 35, 49, 56, 63 and 84. Trends in viral load and CD4 counts in HIV-positive patients were also assessed.

An independent Data and Safety Monitoring Committee (DSMC) – was established to supervise the study and to monitor the trial subjects, and to assess the progress of the clinical trial and the safety data. The DSMC met at predetermined intervals to review the unblinded results to decide whether to withdraw a subject for safety reasons and recommend to the sponsor whether to continue, modify or stop the clinical trial.

### Evaluation of Immunogenicity

The samples were labelled and sent to the lab (Synexa Life Sciences) for the immunogenicity testing. PBMC processing was done at most 4 hours after bleeding.

Cellular mediated immunity was assessed using ELISPOT and ELISA techniques. IFN γ Spot Forming Units were measured in the trial subjects’ peripheral blood after stimulation with up to five stimuli [ESAT 6, CFP10 16 kDa, Ag85B and 38 kDa *M. tuberculosis* antigens, and protein purified derivative (PPD)] for 18 hours. A long-term interferon- γ assay (WHO assay) was performed by stimulating the whole blood for seven days with PPD (reference). A commercial T cell Interferon gamma release assay (TSPOT TB assay) was also performed. Humoral response were studied using the commercial kitt Pathozyme TB Complex Assay (Omega Diagnostics, Ltd. Alva, Scotland, UK) to detect IgG against 16 kDa and 38 kDa *M. tuberculosis* antigens. Time points were Days 0, 28 (pre-1^st^ dose), 35, 56 (pre-2^nd^ dose) and 63. All the samples were frozen in liquid nitrogen and analyzed at the end of the trial to minimize inter assay variability.

Individual data were listed by subject and time point including changes from baseline and whether or not the subject was classified as a responder. Responders at each time point were defined as those subjects for whom the change from baseline induced by vaccination for the specific stimulus and time point was higher than the median of the change from baseline obtained in the placebo group (for a given HIV-status) at the same time point and stimulus.

### Statistical Methods

Given that this was a phase II safety and immunogenicity trial a formal sample size calculation was not made. In any case, the numbers in each group (12 subjects), are comparable with the standard sample sizes used in these types of studies.

Changes from baseline were analysed using a repeated measures ANCOVA with treatment and time point as fixed effects and the baseline measurement (Day 28, pre-inoculation) as a covariate in the analysis. A separate analysis was performed for each of the measured immunogenicity variables for TB ELISPOT (CFP10, HPHA, ESAT 6, 16 kDa, Ag85B and 38 kDa *M. tuberculosis* antigens, PPD, PHA and a negative control), IFN-γ (PPD, PHA and negative control), and TB Complex (pathozyme) assay. Confidence intervals for the change from baseline versus placebo were obtained for each dose and day. The placebo subjects were pooled for the purpose of the analysis. The analysis was repeated for the population as a whole and stratified by HIV status.

An additional non-parametric analysis was also performed. The same analysis of variance (ANOVA) model was used (without the baseline covariate); however the change from baseline (dependent variable) was substituted by a rank transformation. The rank transformation was performed by ordering all the changes from baseline in consecutive, ascending order, separately for each parameter and group (i.e., overall, HIV negative and HIV positive) and then ranking these from 1 to ‘n’. The ranked values were included into the analysis.

### Ethics

The study was carried out in accordance with the principles of the Declaration of Helsinki and the Guidelines for Good Clinical Practice. An Independent Ethics Committee (IEC) of the University of the Free State, Bloemfontein, South Africa, and the South African Medicines Control Council (MCC) approved the study. The study protocol was reviewed and approved, being registered as ClinicalTrials.gov identifier: NCT01136161.

## Results

From the 111 subjects enrolled in the study, 95 subjects were ultimately randomized (HIV-: 48 subjects; HIV+: 47 subjects) to receive either placebo or RUTI (Fig. 1). The baseline characteristics for the 95 participants were described in Table 1. The age and weight [mean (SD)], were similar by study arm, however the percent female was higher in HIV-infected compared to HIV-uninfected cohort (except for the placebo group). The large majority of participants were African, except in the group of 25 µg HIV-uninfected participants, where the proportion of African participants was 50%.

**Table 1:** Demographic and anthropometric baseline data by treatment and HIV-status.

	Placebo		5 µg RUTI		25 µg RUTI		50 µg RUTI	
	HIV−	HIV+	HIV−	HIV+	HIV−	HIV+	HIV−	HIV+
	(n = 12)	(n = 12)	(n = 12)	(n = 11)	(n = 12)	(n = 12)	(n = 12)	(n = 12)
**Age (years); mean (SD)**	32.8 (9.4)	35.7 (7.8)	35.8 (9.4)	33.0 (6.3)	36.1 (11.0)	31.2 (7.3)	32.1 (8.5)	32.9 (8.6)
**Ethic origin (%)**								
** African**	66.7	66.7	66.7	72.7	50.0	100.0	83.3	91.7
** Others**	33.3	33.3	25.0	18.2	50.0	0.0	16.7	8.3
**Female (%)**	83.3	83.3	41.7	72.7	58.3	91.7	41.7	83.3
**Weight (kg);mean (SD)**	74.2 (13.8)	69.1 (13.2)	75.9 (16.3)	74.7 (21.5)	83.8 (22.0)	76.6 (14.6)	75.1 (19.4)	71.8 (9.8)

SD, standard deviation.

Patient recruitment was done from July 2010 to January 2011, and follow-up until April 2011.

### Vaccine Safety

Safety data was available for 95 subjects (HIV-negative: 48 and HIV-positive: 47). Overall 90 (95%) subjects [19 (79%) of the 24 subjects in the placebo group; 71 (100%) of the 71 subjects in the RUTI group] reported one or more adverse events (AEs). The most frequently occurring AEs were injection site reactions. All the patients vaccinated with RUTI (both HIV-positive and -negative) reported at least one injection site reaction; while in placebo group the average was lower (i.e. 50% in HIV-positive and 75% in HIV-negative). Among patients vaccinated with RUTI the percent of each local site reaction was as follows: erythema (91% in HIV+ & 92% in HIV−), induration (94% in HIV+ & 92% in HIV−), local nodules (46% in HIV+ & 25% in HIV−), local pain (66% in HIV+ & 75% in HIV−), sterile abscess (defined as pustular lesion with negative culture) (6% both in HIV+ & in HIV−), swelling (74% in HIV+ & 83% in HIV−), ulcer (20% in HIV+ & 11% in HIV−). Among patients vaccinated with placebo the percent of each local site reaction was as follows: erythema (42% in HIV+ & 42% in HIV−), induration (25% in HIV+ & 25% in HIV−), local pain (8% in HIV+ & 42% in HIV−), swelling (33% in HIV+ & 17% in HIV−). No nodules, abscesses nor ulcers were observed. Treatment emergent adverse events by treatment, HIV-status and System Organ Class are detailed in Table 2. Treatment emergent adverse events by treatment, HIV-status and Preferred Term are detailed in Table S1.

**Table 2:** Treatment emergent adverse events by treatment, HIV-status and System Organ Class.

	Placebo		5 µg RUTI		25 µg RUTI		50 µg RUTI	
System Organ Class	HIV−	HIV+	HIV−	HIV+	HIV−	HIV+	HIV−	HIV+
	(n = 12)	(n = 12)	(n = 12)	(n = 11)	(n = 12)	(n = 12)	(n = 12)	(n = 12)
	n (%) E	n (%) E	n (%) E	n (%) E	n (%) E	n (%) E	n (%) E	n (%) E
Gastrointestinaldisorders	3 (12.50) 3	–	1 (4.35) 1	1 (4.35) 1	1 (4.17) 1	–	–	2 (8.33) 2
General disorders andadministration siteconditions	9 (37.50) 20	6 (25.00) 14	12 (52.17) 67	11 (47.83) 53	12 (50.00) 94	12 (50.00) 108	12 (50.00) 96	12 (50.00) 120
Infections andinfestations	3 (12.50) 3	4 (16.67) 4	3 (13.04) 3	4 (17.39) 4	2 (8.33) 2	5 (20.83) 6	4 (16.67) 4	7 (29.17) 9
Injury, poisoningand proceduralcomplications	1 (4.17) 1	–	–	–	1 (4.17) 1	–	–	1 (4.17) 1
Investigations	–	–	–	–	–	–	–	1 (4.17) 1
Musculoskeletal andconnective tissuedisorders	1 (4.17) 2	1 (4.17) 1	–	1 (4.35) 1	1 (4.17) 1	1 (4.17) 1	3 (12.50) 3	1 (4.17) 1
Nervous systemdisorders	3 (12.50) 6	3 (12.50) 3	3 (13.04) 3	2 (8.70) 2	1 (4.17) 1	2 (8.33) 3	6 (25.00) 7	5 (20.83) 5
Psychiatric disorders	–	–	1 (4.35) 1	–	–	–	–	–
Respiratory, thoracicand mediastinaldisorders	1 (4.17) 1	–	–	1 (4.35) 1	–	1 (4.17) 1	–	1 (4.17) 1
Skin andsubcutaneoustissue disorders	–	1 (4.17) 1	1 (4.35) 3	1 (4.35) 1	1 (4.17) 1	–	1 (4.17) 1	–
Vascular disorders	–	–	–	–	–	–	–	1 (4.17) 1

N: Number of subjects inoculated; n: Number of subjects with adverse events; E: Number of adverse events. Percentages calculated as the percentage of the total number of subjects inoculated in each treatment group.

The general occurrence of the local injection site reactions seemed to be more frequent from Day 31 onwards and did not subside on Day 84 (follow-up visit). More skin reactions were observed in HIV-negative and HIV-positive groups at the higher doses (25 µg and 50 µg) of RUTI. The 23 subjects of the group of 5 µg group reported 117 occurrences (22%), 65 (26%) in HIV-negative and 52 (19%) in HIV-positive; the 24 subjects of the 25 µg group reported 194 occurrences (37%); 89 (36%) in HIV-negative and 105 (38%) in HIV-positive; the 24 subjects of the 50 µg group reported 211 occurrences (40%), 93 (38%) in HIV-negative and 118 (43%) in HIV-positive. It should be noted that there was a high number of local nodules (twenty-five subjects had 43 occurrences of local nodules), which occurred more frequently in HIV-positive subjects and worsened after the second injection. Of the 25 participants with injection site local nodules, four subjects underwent incision, surgical drainage and culture of local abscess. The cultures’ results were indicative of sterile abscesses. 11 subjects had 15 occurrences of ulcers. In general, the occurrences of ulcers were assessed as mild and moderate in intensity.

The highest pain scores on the visual analogue scale (VAS) were reported on Day 31, being the median score value of 3, 3.5 and 20 in the 5 µg, 25 µg and 50 µg RUTI dose groups respectively in HIV-positive group, and with less intensity on Day 59. In HIV-negative group, the highest scores were observed on Day 59, being the median score value of 1, 3 and 9 in the 5 µg, 25 µg and 50 µg RUTI dose groups respectively and with less intensity on Day 31. However, in both groups most of the subjects reported no pain (score 0) [Fig. S1, Table S4].

Overall the results of the chest CT scan were assessed as normal at the two time points, Day 28 and Day 84 (Follow-up). A few subjects had abnormal results assessed as NCS and four subjects had CS finding on Day 84 (Follow-up) as active TB was suspected; however, after consulting with the investigators, assessment of active TB was discarded.

Generally, the reported AEs were assessed as mild and moderate in intensity, except for four AEs, which were assessed as severe in intensity.

Related to the clinical laboratory parameters, five subjects (HIV-negative three subjects; HIV-positive two subjects) presented with clinically significant (CS) biochemistry values which on repeat were either assessed as not CS or had returned within acceptable limits. Although a number of subjects in both the HIV-negative and HIV-positive dose groups had abnormal ECG findings, all abnormal findings were assessed as not CS under investigators’ criteria. There were no ECG findings that were recorded as AEs and no differences were observed between different groups or time points (similar findings were recorded before and after treatment).

The HIV viral load and the CD4 count did not change over time in HIV-infected and uninfected RUTI or placebo recipients (see Table S2 and S3).

Overall the randomized subjects, one had a serious adverse event (SAEs) during the study: one subject in the RUTI 25 µg HIV-group had a local injection site sterile abscess and was hospitalized overnight for observation (which was the reason for classifying the AE as serious); causality was assessed as very likely and by the end of the study the outcome had resolved.

On the other hand, one subject in the RUTI 50 µg HIV-positive group was withdrawn (due to several Treatment Emergent Adverse Events -TEAEs) and after withdrawal suffered further adverse events: fecaloma, injection site swelling, hemoptysis and increased creatine kinase in the blood. Whilst these events were assessed as moderate and mild in intensity, fecaloma was assessed as severe in intensity; causality was assessed as not related, according to the investigator criteria. The causality of hemoptysis and increased creatine kinase was assessed as doubtful and possible, respectively; no deaths occurred.

### Vaccine Immunogenicity

The FAS (Full Analysis Set) and PPS (Per Protocol Set) yielded similar results; however, as the PPS is considered as the primary analysis, results of this population are presented.

### TB ELISPOT

**Figure 3:**
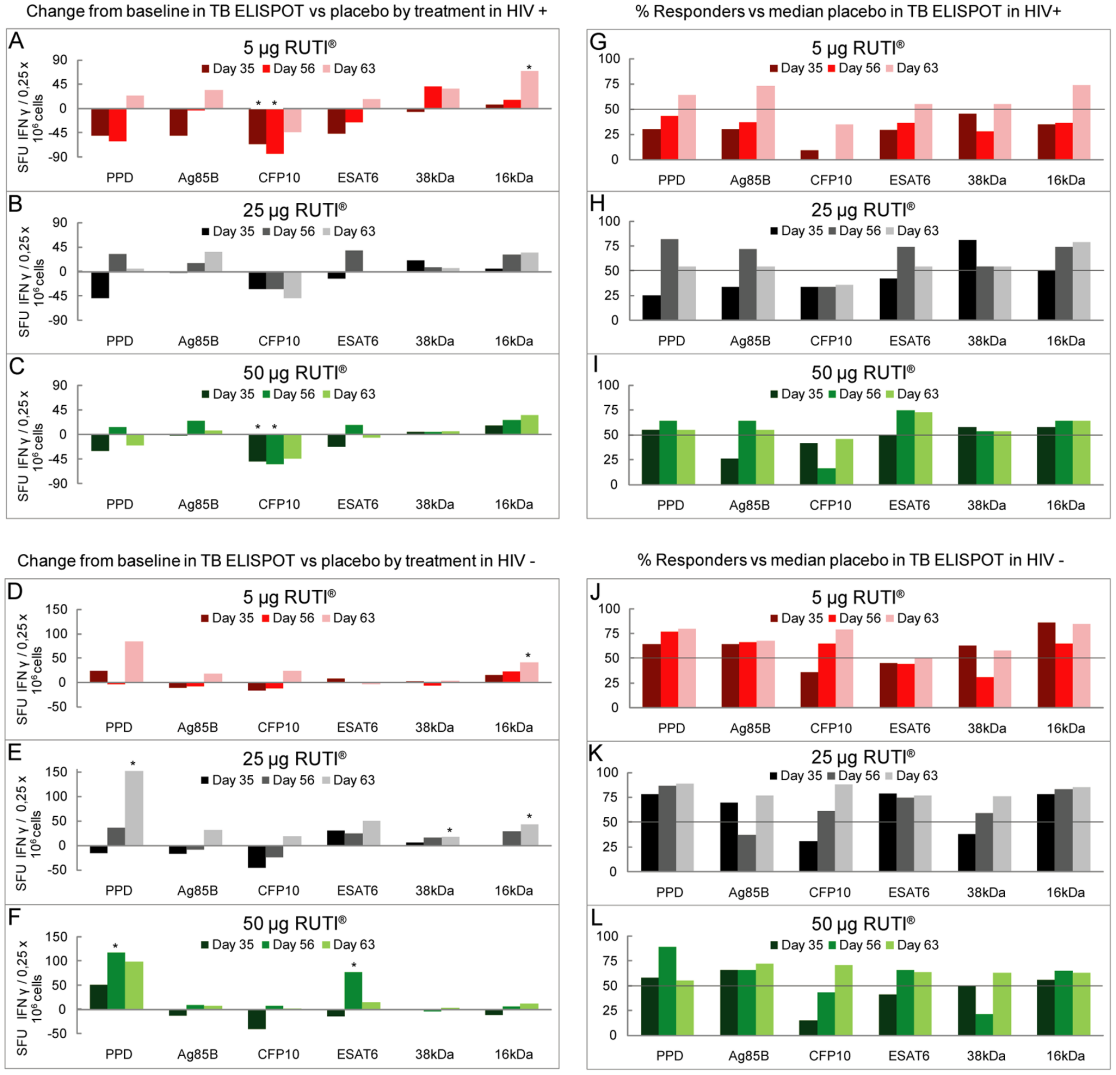
TB ELISPOT. A–F represents the change from baseline in TB ELISPOT vs placebo by treatment in HIV-positive (A, B, C) and HIV-negative (D, E, F) groups. G–L represents the percentage of responders in TB ELISPOT by treatment in HIV-positive (G, H, I) and HIV-negative (J, K, L) subjects. Responders at each time point were defined as those subjects for whom the change from baseline induced by vaccination for the specific stimulus and time point was higher than the median of the change from baseline obtained in the placebo group (i.e. horizontal bar) at the same time point and stimulus. *Statistically significant change from baseline (p-value <0.05).

Results from ELISPOT assays in HIV-negative subjects using 5 µg or 25 µg RUTI showed a good polyantigenic response. This response was slightly increased after the second vaccination, becoming significant at Day 63 compared to baseline in PPD, 38 kDa and 16 kDa. Nevertheless, the injection of 50 µg RUTI loses the polyantigenic response, favouring the response towards ESAT-6 (Fig. 3D–F). These results were supported by the percentage of responders achieved (Fig. 3J–L).

The immune response in HIV-positive subjects was lower than in HIV-negative, especially at the lowest dose (5 µg RUTI) which required a second inoculation to increase the response to some antigens (PPD, Ag85B, ESAT6 and 16 kDa). However, the response after the first injection of 25 or 50 µg of RUTI showed equal or higher values than after the second inoculation (Fig. 3A–C). Similar results were obtained when comparing the percentage of responders (Fig. 3G–I).

### WHO Test

**Figure 4:**
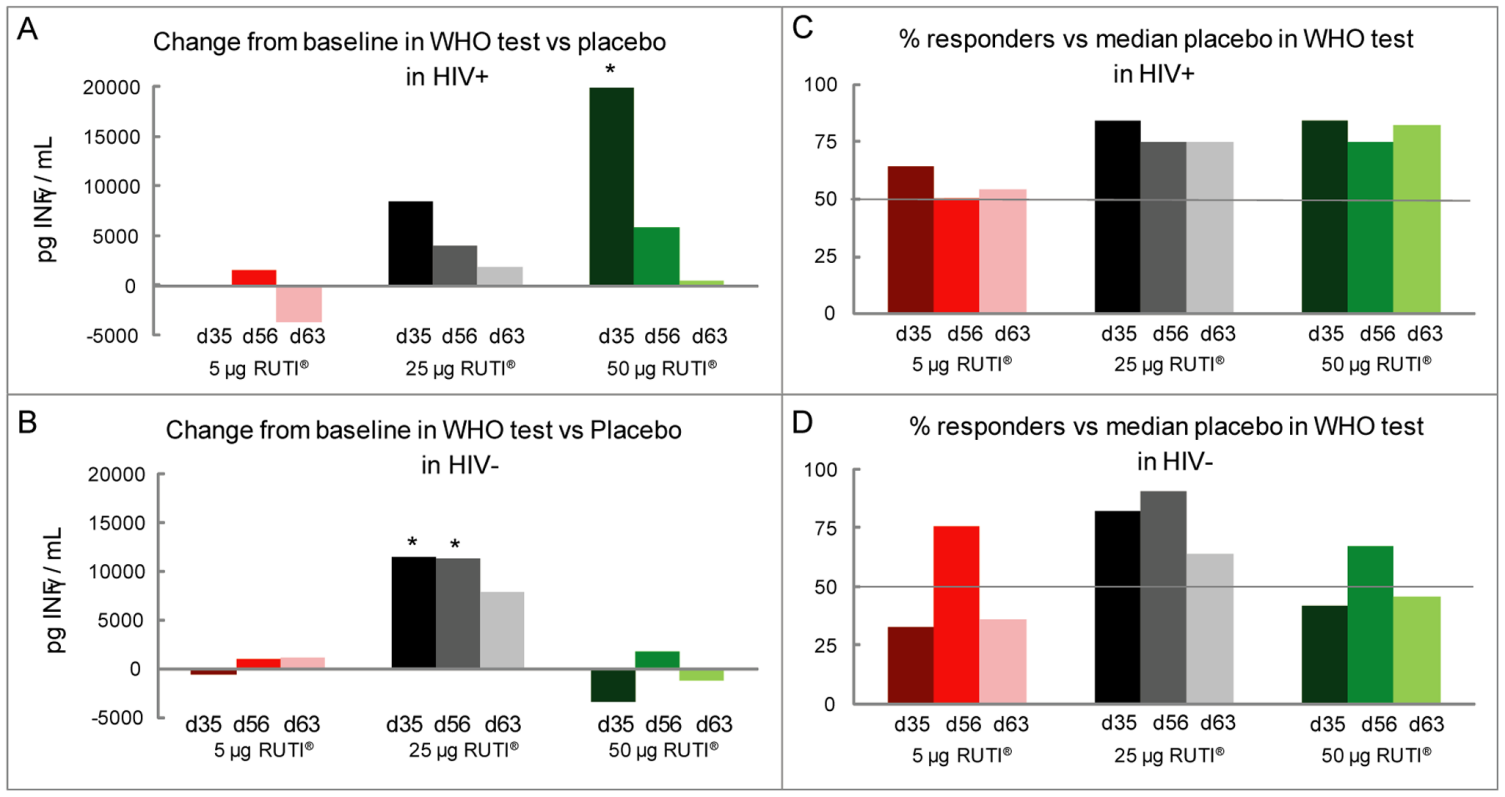
WHO test. A, B represents the change from baseline in the WHO test vs placebo by treatment and HIV-status. C, D represents the percentage of responders in the WHO test by treatment and HIV-status. Responders at each time point were defined as those subjects for whom the change from baseline induced by vaccination for the specific stimulus and time point was higher than the median of the change from baseline obtained in the placebo group (i.e. horizontal bar) at the same time point and stimulus. *Statistically significant change from baseline (p-value <0.05).

The WHO test assay in HIV-negative subjects compared to placebo showed a clear improvement from baseline in the 25 µg group at all-time points, which achieved statistical significance on Day 35 and Day 56. However, after the 50 µg RUTI dose this peak decreased (Fig. 4B). These results are consistent with the percentage of responders achieved (Fig. 4D).

In HIV-positive subjects a clear improvement was observed in the 50 µg group at Day 35, which achieved statistical significance. In the 25 µg group a good response was also observed on Day 35 although this was lower in magnitude than that observed after the 50 µg dose (Fig. 4A). In the 25 µg group, a 75–85% of responders at the different time points were achieved. This percentage was maintained in the 50 µg group (Fig. 4C).

### Humoral Response

There were no relevant changes against 16 kDa and 38 kDa antigens (Pathozyme TB complex assay) at any time point for either HIV negative or HIV positive subjects (at all doses).

## Discussion

We report the results of the first randomized, double-blind, placebo-controlled Phase II trial of RUTI vaccine conducted in South Africa in patients with LTBI. The RUTI safety profile was considered acceptable since only two subjects had SAEs; most AEs reported were mild in intensity, which means the event did not interfere with daily activities, and were well tolerated. The most frequent local reactions at the injection site were local nodules, erythema, induration, swelling and pain as have been recorded in other clinical trials [9], [10]. There were no deaths during the study. It is important to stress the high number of local nodules observed, and that in four subjects who developed abscesses, drainage was required. A proportion of local self-resolving nodules at the site of vaccination was previously reported in TB vaccinated subjects with previous or latently TB infection [11]. We have not been able to discern what the origin of such reaction was because it was not related with the intensity of the specific immune response monitored. Related to the higher number of local nodules at the injection site reported in HIV-positive subjects, as this group of patients showed a lower immunological response than in HIV-negative ones, local nodulation cannot be correlated with the systemic immunological response. The hypothesis to explain this occurrence of local self-resolving nodules at the site of vaccination could be because HIV infection promotes local inflammation of adipose tissue, thus increasing the inflammatory response caused by the subcutaneous delivery of the vaccine [12]. This local reactogenicity should be closely monitored in future trials by doing extra visits considering the time when these events occurred in this trial.

The injection site pain was also a common local adverse event, which spontaneously resolved one week after vaccination (data not shown). The intensity of pain was considered by the subjects as mild and was similar to the intensity of pain demonstrated in the Phase I trial [8]. Of note, one of the most frequently occurring local adverse events observed in the Phase I trial was twitching at the injection site, occurring in all the treatment groups (RUTI and placebo) [8]. One of the objectives of RUTI development was to avoid this adverse event; twitching was not observed in any of the three doses of the vaccine used in this trial, reinforcing the idea that the addition of a stabilising excipient (i.e., sucrose) in the RUTI vaccine formulation has proven to be essential.

No clinically significant changes in laboratory biochemistry and/or haematology were observed. Safety results are in line with those obtained in previous studies carried out, in similar conditions (i.e. vaccinating with *M. tuberculosis* antigens in already infected subjects) even if it is difficult to compare results because there are differences in vaccines’ origin, different regimens and target population [9], [10], [13].

RUTI vaccine did not cause any variation in the HIV viral load or CD4 counts, thus the vaccination did not affect the evolution of HIV infection, unlike some other vaccines which induced a transient increase of the viral load concurrently associated to the loss of CD4 counts [14], although this must be interpreted with caution as the number of subjects were relatively low.

The mechanisms of immune protection against human TB are not fully known, however, it is recognized a protective immunity against *M. tuberculosis* mainly due to the generation of Th1-type cellular response characterized by interferon-gamma (IFN-γ) production [15]–[18]. Therefore, the current TB vaccine (BCG), and most new vaccines under development aim to induce this immunity [18].

Different experimental models of TB in mice have demonstrated that *M. tuberculosis* grew again after short-term chemotherapy, and this was not followed by an immediate stimulation of immunity. One of the aims of this vaccine was to fill the immunological gap left by short-term therapy for *M. tuberculosis* infection. Therefore, the original idea of introducing this vaccine was to “boost” the dominant immunological response against growing *M. tuberculosis* that already exists in the host. Moreover, this vaccine was also designed to trigger a new immunological response against antigens of the latent bacilli [6].

All the pre-clinical and phase I clinical trials conducted with the vaccine RUTI showed (as it has been published elsewhere) that it is able to generate a cellular immune response measurable as IFN-γ secretion. In the present study, ELISPOT results in HIV-negative individuals with 5 µg or 25 µg RUTI showed a good immune response, with a slight increase after the second vaccination. However, the injection of 50 µg RUTI loses the polyantigenic response, favouring the response towards ESAT-6. How this response can also influence the memory immunity is difficult to explain, as it seems that there is an excessive response of effectors cells that may compromise the memory response. The immunological response in HIV-positive subjects displayed a similar polyantigenic profile after the first injection of 25 or 50 µg RUTI which was not increased after the second injection. It is interesting to stress that even when we do expected an increase of the cellular immune response as a consequence of a vaccine intake, we detected a decreased response to CFP10 (in both HIV-positive and HIV-negative groups). This fact is of importance as CFP-10 decreased response is linked to protection [19]. Although a variable pattern of responses has been seen, the immunological results from this study showed that a polyantigenic response was triggered by RUTI vaccination against structural and secreted *M. tuberculosis* antigens, and suggest that only one vaccination will be needed to increase the immune response after short term chemotherapy [6], [20].

The memory immune response using the WHO test assay showed a dose response relationship in the HIV-positive subjects after a single inoculation which lessened after the second inoculation. The best response was obtained using 50 µg RUTI vaccine and a good response was also observed in 25 µg RUTI. Related to the HIV-negative subjects, it is noticed a response peak with 25 µg RUTI dose after the first inoculation which was sustained after the second inoculation. Intriguingly, we have observed a decrease in this response after the 50 µg RUTI dose, caused by a sort of saturation phenomenon, that would require further studies to be confirmed. The percentage of responders was assessed as a post-hoc analysis, and was done in order to have an overall picture of the global immunologic response. Results obtained were consistent with the ones obtained when analysing the intensity of the immune response, as previously mentioned.

The intensity of the immune response, which is of a magnitude of about 2 log variation, is significantly lower than that reported in other clinical trials in subjects with LTBI [11]. However the magnitudes of responses are similar to those seen with BCG and SSI H1 vaccines in adults [9], [13]. The antigens used in this study are an extract of *M. tuberculosis* containing several antigens but all in low concentrations, which are less reactive than peptides used for peripheral blood mononuclear cell stimulation [21], [22].

All the attempts to compare the immune responses between different studies must be viewed with caution since there are differences in origin of vaccines, dosing regimens and the diverse populations studied and assays used [9], [10], [13]. Until now, none of the current vaccines against *M. tuberculosis* infection are tested as therapeutic agents after short-term chemotherapy; therefore no real comparison could be made with the results obtained in this CT. Overall, these results are consistent with our preliminary phase I results [8], where the administration of 25 µg RUTI achieved the best cellular polyantigenic response, and where no relevant humoral response was detected.

In summary, these results show a relative acceptable safety, however the induction of local abscess requires further research. Equally, the immunogenicity profile of RUTI vaccine in LTBI subjects, even being variable among groups, allows us considering one single injection of one of the highest doses in future trials, preceded by an extended safety clinical phase.

## Supporting Information

Figure S1 **Mean pain rating by treatment in HIV-positive (A) and HIV-negative (B) subjects.** Pain intensity at the injection site was assessed subjectively by patients using a visual analogical scale (VAS) ranging from 0 (no pain) to 100 (maximum pain).
(TIF)

Table S1 **Treatment emergent adverse events by treatment, HIV-status and System Organ Class.**
(DOC)

Table S2 **Treatment emergent adverse events by treatment, HIV-status and preferred term.**
(DOC)

Table S3 **Mean CD4 by treatment and change from baseline by time point, treatment and HIV-status.**
(DOC)

Table S4 **Pain rating (Visual Analogue Scale) by time point, treatment and HIV-status.**
(DOC)

Checklist S1 **CONSORT Checklist.**
(DOC)

Protocol S1 **Trial Protocol.**
(PDF)

## References

[1] World Health Organization. Global tuberculosis control: epidemiology s, financing: WHO report 2011.

[2] Parrish NM, Dick JD, Bishai WR (1998) Mechanisms of latency in Mycobacterium tuberculosis. Trends Microbiol 6: 107–112. 9582936 10.1016/s0966-842x(98)01216-5

[3] Harris A MD, Graham. (2004) TB/HIV a clinical manual. 2nd Edition. WHO.

[4] Comstock GW (1999) How much isoniazid is needed for prevention of tuberculosis among immunocompetent adults? Int J Tuberc Lung Dis 3: 847–850. 10524579

[5] Cardona PJ (2010) Revisiting the natural history of tuberculosis. The inclusion of constant reinfection, host tolerance, and damage-response frameworks leads to a better understanding of latent infection and its evolution towards active disease. Arch Immunol Ther Exp (Warsz) 58: 7–14. 20049645 10.1007/s00005-009-0062-5

[6] Cardona PJ (2006) RUTI: a new chance to shorten the treatment of latent tuberculosis infection. Tuberculosis (Edinb) 86: 273–289. 16545981 10.1016/j.tube.2006.01.024

[7] Cardona PJ (2009) A dynamic reinfection hypothesis of latent tuberculosis infection. Infection 37: 80–86. 19308318 10.1007/s15010-008-8087-y

[8] Vilaplana C, Montane E, Pinto S, Barriocanal AM, Domenech G, et al (2010) Double-blind, randomized, placebo-controlled Phase I Clinical Trial of the therapeutical antituberculous vaccine RUTI. Vaccine 28: 1106–1116. 19853680 10.1016/j.vaccine.2009.09.134

[9] Hoft DF, Blazevic A, Abate G, Hanekom WA, Kaplan G, et al (2008) A new recombinant bacille Calmette-Guerin vaccine safely induces significantly enhanced tuberculosis-specific immunity in human volunteers. J Infect Dis 198: 1491–1501. 18808333 10.1086/592450 PMC2670060

[10] van Dissel JT, Arend SM, Prins C, Bang P, Tingskov PN, et al (2010) Ag85B-ESAT-6 adjuvanted with IC31 promotes strong and long-lived Mycobacterium tuberculosis specific T cell responses in naive human volunteers. Vaccine 28: 3571–3581. 20226890 10.1016/j.vaccine.2010.02.094

[11] van Dissel JT, Soonawala D, Joosten SA, Prins C, Arend SM, et al (2011) Ag85B-ESAT-6 adjuvanted with IC31(R) promotes strong and long-lived Mycobacterium tuberculosis specific T cell responses in volunteers with previous BCG vaccination or tuberculosis infection. Vaccine 29: 2100–2109. 21256189 10.1016/j.vaccine.2010.12.135

[12] Giralt M, Domingo P, Villarroya F (2011) Adipose tissue biology and HIV-infection. Best Pract Res Clin Endocrinol Metab 25: 487–499. 21663842 10.1016/j.beem.2010.12.001

[13] Von Eschen K, Morrison R, Braun M, Ofori-Anyinam O, De Kock E, et al (2009) The candidate tuberculosis vaccine Mtb72F/AS02A: Tolerability and immunogenicity in humans. Hum Vaccin 5: 475–482. 19587528 10.4161/hv.8570

[14] Castro P, Plana M, Gonzalez R, Lopez A, Vilella A, et al (2009) Influence of a vaccination schedule on viral load rebound and immune responses in successfully treated HIV-infected patients. AIDS Res Hum Retroviruses 25: 1249–1259. 19943787 10.1089/aid.2009.0015

[15] Agger EM, Andersen P (2001) Tuberculosis subunit vaccine development: on the role of interferon-gamma. Vaccine 19: 2298–2302. 11257351 10.1016/s0264-410x(00)00519-3

[16] Kaufmann SH, Baumann S, Nasser Eddine A (2006) Exploiting immunology and molecular genetics for rational vaccine design against tuberculosis. Int J Tuberc Lung Dis 10: 1068–1079. 17044198

[17] Flynn JL (2004) Immunology of tuberculosis and implications in vaccine development. Tuberculosis (Edinb) 84: 93–101. 14670350 10.1016/j.tube.2003.08.010

[18] Hanekom WA, Dockrell HM, Ottenhoff TH, Doherty TM, Fletcher H, et al (2008) Immunological outcomes of new tuberculosis vaccine trials: WHO panel recommendations. PLoS Med 5: e145. 18597551 10.1371/journal.pmed.0050145 PMC2443198

[19] Lin PL, Dietrich J, Tan E, Abalos RM, Burgos J, et al. The multistage vaccine H56 boosts the effects of BCG to protect cynomolgus macaques against active tuberculosis and reactivation of latent Mycobacterium tuberculosis infection. J Clin Invest 122: 303–314. 22133873 10.1172/JCI46252 PMC3248283

[20] Guirado E, Gil O, Caceres N, Singh M, Vilaplana C, et al (2008) Induction of a specific strong polyantigenic cellular immune response after short-term chemotherapy controls bacillary reactivation in murine and guinea pig experimental models of tuberculosis. Clin Vaccine Immunol 15: 1229–1237. 18524883 10.1128/CVI.00094-08 PMC2519306

[21] Hawkridge T, Scriba TJ, Gelderbloem S, Smit E, Tameris M, et al (2008) Safety and immunogenicity of a new tuberculosis vaccine, MVA85A, in healthy adults in South Africa. J Infect Dis 198: 544–552. 18582195 10.1086/590185 PMC2822902

[22] Sander CR, Pathan AA, Beveridge NE, Poulton I, Minassian A, et al (2009) Safety and immunogenicity of a new tuberculosis vaccine, MVA85A, in Mycobacterium tuberculosis-infected individuals. Am J Respir Crit Care Med 179: 724–733. 19151191 10.1164/rccm.200809-1486OC PMC2858810

