# Supplementary figures and images for: Safety, Tolerability, and Immunogenicity of the Novel Antituberculous Vaccine RUTI: Randomized, Placebo-Controlled Phase II Clinical Trial in Patients with Latent Tuberculosis Infection
Source: PLoS One. 2014 Feb 26;9(2):e89612. doi: 10.1371/journal.pone.0089612 (PMC3935928; doi:10.1371/journal.pone.0089612)

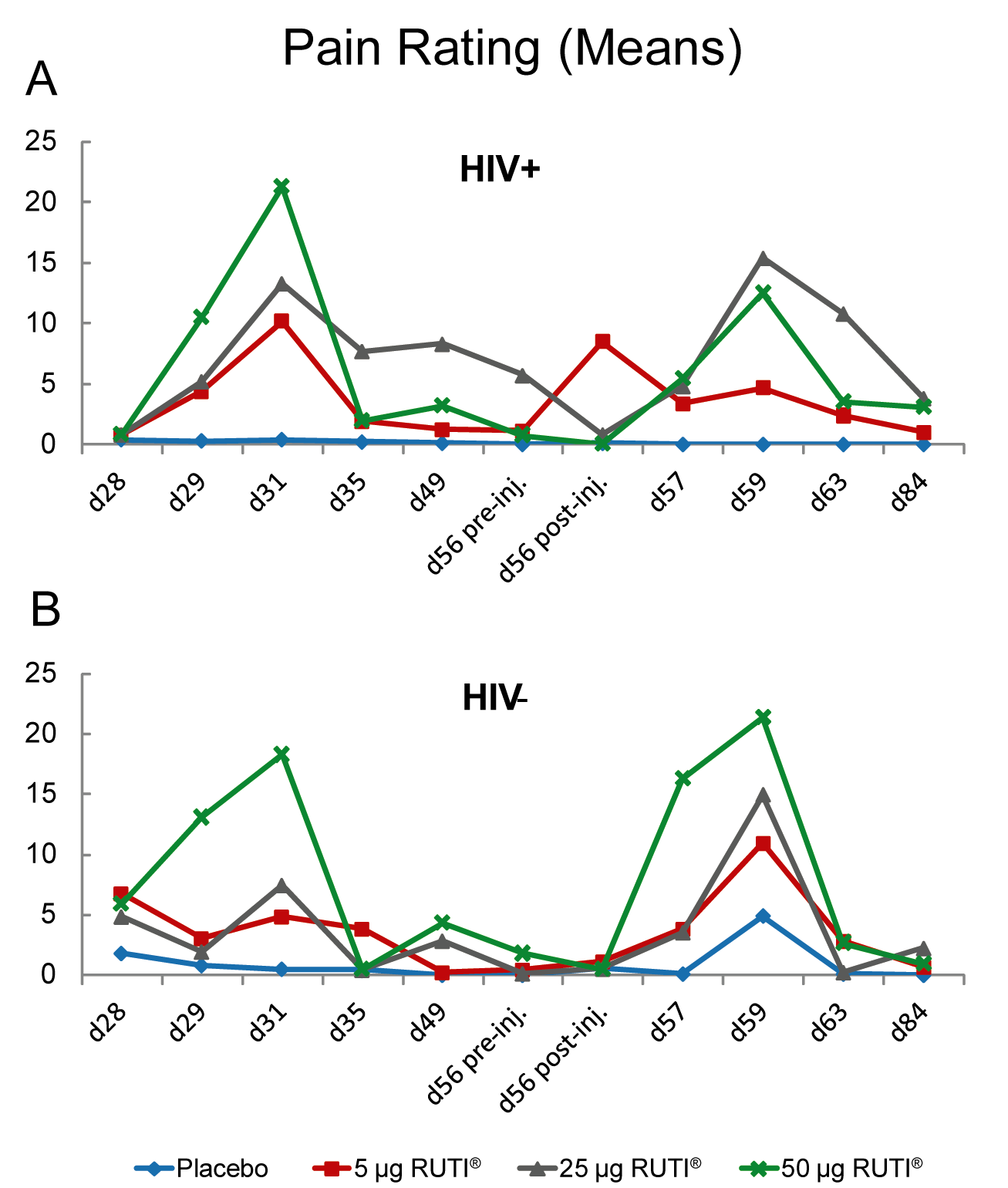

Supplement: Figure S1 — Mean pain rating by treatment in HIV-positive (A) and HIV-negative (B) subjects. Pain intensity at the injection site was assessed subjectively by patients using a visual analogical scale (VAS) ranging from 0 (no pain) to 100 (maximum pain). (TIF) [file pone.0089612.s001.tif]
