# Supplementary material for: Safety, Tolerability, and Immunogenicity of the Novel Antituberculous Vaccine RUTI: Randomized, Placebo-Controlled Phase II Clinical Trial in Patients with Latent Tuberculosis Infection
Source: PLoS One. 2014 Feb 26;9(2):e89612. doi: 10.1371/journal.pone.0089612 (PMC3935928; doi:10.1371/journal.pone.0089612)
Supplement: Table S1 — Treatment emergent adverse events by treatment, HIV-status and System Organ Class. (DOC) [file pone.0089612.s002.doc]

**Table S1. Treatment emergent adverse events by treatment, HIV-status and preferred term**

|  | | **Placebo** | |  | **5 μg RUTI®** |  | **25 μg RUTI®** |  | | **50 μg RUTI®** | |  |
| --- | --- | --- | --- | --- | --- | --- | --- | --- | --- | --- | --- | --- |
| **Preferred term** | | **HIV-** | | **HIV+** | **HIV-** | **HIV+** | **HIV-** | **HIV+** | | **HIV-** | | **HIV+** |
|  | | **(n=12)** | | **(n=12)** | **(n=12)** | **(n=11)** | **(n=12)** | **(n=12)** | | **(n=12)** | | **(n=12)** |
|  | | **n (%) E** | | **n (%) E** | **n (%) E** | **n (%) E** | **n (%) E** | **n (%) E** | | **n (%) E** | | **n (%) E** |
| **Administration site conditions** | |  | |  |  |  |  |  | |  | |  |
| Local nodule^^ | | - | | - | 1 (4.35) 1 | - | 3 (12.50) 6 | 9 (37.50) 18 | | 5 (20.83) 7 | | 7 (29.17) 11 |
| Erythema | | 5 (20.83) 6 | | 5 (20.83) 5 | 9 (39.13) 14 | 9 (39.13) 16 | 12 (50.00) 27 | 11 (45.83) 24 | | 12 (50.00) 20 | | 12 (50.00) 27 |
| Induration | | 3 (12.50) 4 | | 3 (12.50) 3 | 12 (52.17) 20 | 10 (43.48) 19 | 11 (45.83) 25 | 11 (45.83) 26 | | 10 (41.67) 20 | | 12 (50.00) 28 |
| Irritation | | - | | - | - | 1 (4.35) 1 | - | - | | - | | - |
| Pain | | 5 (20.83) 7 | | 1 ( 4.17) 1 | 9 (39.13) 14 | 4 (17.39) 7 | 7 (29.17) 8 | 8 (33.33) 13 | | 11 (45.83) 23 | | 9 (37.50) 17 |
| Pruritus | | - | | - | 1 ( 4.35) 1 | 1 ( 4.35) 1 | - | - | | 1 ( 4.17) 2 | | - |
| Sterile abscess^^ | | - | | - | - | - | 1 (4.17) 2 | 1 (4.17) 2 | | 1 (4.17) 1 | | 1 (4.17) 1 |
| Swelling | | 2 ( 8.33) 2 | | 4 (16.67) 4 | 10 (43.48) 15 | 5 (21.74) 8 | 9 (37.50) 19 | 10 (41.67) 21 | | 11 (45.83) 19 | | 11 (45.83) 28 |
| Ulcer | | - | | - | - | - | 2 (8.33) 4 | 2 (8.33) 2 | | 2 (8.33) 2 | | 5 (20.83) 7 |
| Vesicles | | - | | - | - | - | - | 1 ( 4.17) 1 | | - | | - |
| **General disorders** | | | | | | | | | | | | |
| Chills | | - | | - | - | - | - | - | | 1 (4.17) 1 | | - |
| Fatigue | | - | | - | 1 (4.35) 1 | - | 1 (4.17) 2 | 1 (4.17) 1 | | 1 (4.17) 1 | | 1 (4.17) 1 |
| Influenza like illness | | - | | - | - | - | 1 (4.17) 1 | - | | - | | - |
| Malaise | | - | | - | 1 ( 4.35) 1 | 1 ( 4.35) 1 | 1 ( 4.17) 2 | 2 ( 8.33) 2 | | 1 ( 4.17) 1 | | 1 ( 4.17) 1 |
| Oedema peripheral | | - | | 1 ( 4.17) 1 | - | - | - | - | | - | | - |
| Pyrexia | | 1 (4.17) 1 | | - | - | - | - | - | | - | | - |
| **Infections and infestations** | | | | | | | | | | | | |
| Acute sinusitis | - | | - | | - | - | 1 ( 4.17) 1 | | - | - | - | |
| Fungal skin infection | - | | - | | - | - | - | | - | 1 (4.17) 1 | - | |
| Gastroenteritis | - | | - | | - | - | - | | - | - | 1 (4.17) 1 | |
| Hordeolum | - | | - | | - | - | - | | 1 (4.17) 1 | - | - | |
| Influenza | - | | - | | 2 (8.70) 2 | - | - | | 1 (4.17) 2 | - | - | |
| Nasopharyngitis | 2 (8.33) 2 | | 3 (12.50) 3 | | 1 (4.35) 1 | 2 (8.70) 2 | - | | 1 (4.17) 1 | 1 (4.17) 1 | 4 (16.67) 4 | |
| Oral herpes | - | | - | | - | - | 1 (4.17) 1 | | - | - | - | |
| Pulmonary tuberculosis | - | | - | | - | - | - | | - | - | 1 (4.17) 1 | |
| Sinusitis | - | | - | | - | - | - | | - | 1 (4.17) 1 | - | |
| Tooth abscess | - | | 1 (4.17) 1 | | - | - | - | | - | - | - | |
| Upper respiratory tract infection | - | | - | | - | 1 (4.35) 1 | - | | 1 (4.17) 1 | 1 (4.17) 1 | 2 (8.33) 2 | |
| Urinary tract infection | 1 (4.17) 1 | | - | | - | - | - | | 1 ( 4.17) 1 | - | 2 (8.33) 2 | |
| Vaginal infection | - | | - | | - | 1 (4.35) 1 | - | | - | - | - | |
| **Injury, poisoning and procedural complications** | | | | | | | | | | | | |
| Excoriation | - | | - | | - | - | - | | - | - | 1 (4.17) 1 | |
| Muscle strain | 1 (4.17) 1 | | - | | - | - | - | | - | - | - | |
| Whiplash injury | - | | - | | - | - | 1 (4.17) 1 | | - | - | - | |
| **Investigations** | | | | | | | | | | | | |
| Blood creat-pk increased | - | | - | | - | - | - | | - | - | 1 (4.17) 1 | |
| **Musculoskeletal and connective tissue disorders** | | | | | | | | | | | | |
| Arthralgia | 1 (4.17) 1 | | 1 (4.17) 1 | | - | - | - | | - | 1 (4.17) 1 | - | |
| Back pain | 1 (4.17) 1 | | - | | - | - | - | | - | - | - | |
| Muscle spasms | - | | - | | - | - | 1 (4.17) 1 | | - | 1 (4.17) 1 | - | |
| Musculoskeletal chest pain | - | | - | | - | 1 (4.35) 1 | - | | - | 1 (4.17) 1 | - | |
| Myalgia | - | | - | | - | - | - | | - | - | 1 (4.17) 1 | |
| Neck pain | - | | - | | - | - | - | | 1 (4.17) 1 | - | - | |
| **Nervous system disorders** | | | | | | | | | | | | |
| Dizziness | - | | - | | - | 1 (4.35) 1 | - | | - | 1 (4.17) 1 | 1 (4.17) 1 | |
| Arthralgia | 1 (4.17) 1 | | 1 (4.17) 1 | | - | 1 (4.17) 1 | - | | - | 1 (4.17) 1 | - | |
| Back pain | 1 (4.17) 1 | | - | | - | - | - | | - | - | - | |
| Muscle spasms | - | | - | | - | - | 1 (4.17) 1 | | - | 1 (4.17) 1 | - | |
| Musculoskeletal chest pain | - | | - | | - | 1 (4.35) 1 | - | | - | 1 (4.17) 1 | - | |
| Myalgia | - | | - | | - | - | - | | - | - | 1 (4.17) 1 | |
| Neck pain | - | | - | | - | - | - | | - | 1 (4.17) 1 | - | |
| Dizziness | - | | - | | - | 1 (4.35) 1 | - | | - | 1 (4.17) 1 | 1 (4.17) 1 | |
| Head discomfort | - | | - | | - | - | - | | 1 (4.17) 1 | - | - | |
| Headache | 3 (12.50) 5 | | 3 (12.50) 3 | | 2 (8.70) 2 | 1 (4.35) 1 | 1 (4.17) 1 | | 1 (4.17) 2 | 5 (20.83) 6 | 4 (16.67) 4 | |
| Paraesthesia | 1 (4.17) 1 | | - | | - | - | - | | - | - | - | |
| Syncope | - | | - | | 1 (4.35) 1 | - | - | | - | - | - | |
| **Psychiatric disorders** | | | | | | | | | | | | |
| Anxiety | - | | - | | 1 (4.35) 1 | - | - | | - | - | - | |
| **Respiratory, thoracic and mediastinal disorders** | | | | | | | | | | | | |
| Bronchospasm | - | | - | | - | 1 (4.35) 1 | - | | - | - | - | |
| Hemoptysis | - | | - | | - | - | - | | - | - | 1 (4.17) 1 | |
| Oropharyngeal pain | 1 (4.17) 1 | | - | | - | - | - | | - | - | - | |
| Rhinorrhoea | - | | - | | - | - | - | | 1 (4.17) 1 | - | - | |
| **Skin and subcutaneous tissue disorders** | | | | | | | | | | | | |
| Hyperhidrosis | - | | - | | - | - | - | | - | 1 (4.17) 1 | - | |
| Pruritus | - | | 1 (4.17) 1 | | 1 (4.35) 1 | 1 (4.35) 1 | 1 (4.17) 1 | | - | - | - | |
| Pruritus generalised | - | | - | | 1 (4.35) 2 | - | - | | - | - | - | |
| **Vascular disorders** | | | | | | | | | | | | |
| Hypertension | - | | - | | - | - | - | | - | - | 1 (4.17) 1 | |

N: Number of subjects inoculated; n: Number of subjects with adverse events; E: Number of adverse events. Percentages calculated as the percentage of the total number of subjects inoculated in each treatment group

^^ Although the investigator diagnosed all these cases of local nodule as ‘abscess’, the qualification of abscess should only have been used if the presence of pus was proven. Where this was not the case, the event was considered a local nodule. Presence of pus was notified in only 4 cases (subjects who were referred for drainage and sample analysis) i.e., Subjects 216, 114, 232 and 108. All of these events were sterile.
