# Supplementary material for: Safety, Tolerability, and Immunogenicity of the Novel Antituberculous Vaccine RUTI: Randomized, Placebo-Controlled Phase II Clinical Trial in Patients with Latent Tuberculosis Infection
Source: PLoS One. 2014 Feb 26;9(2):e89612. doi: 10.1371/journal.pone.0089612 (PMC3935928; doi:10.1371/journal.pone.0089612)
Supplement: Table S2 — Treatment emergent adverse events by treatment, HIV-status and preferred term. (DOC) [file pone.0089612.s003.doc]

**Table S2. Viral Load by treatment and change from baseline by time point and treatment**

| Day/Observation/ Intensity | Placebo | | 5 µg RUTI® | | 25 µg RUTI® | | 50 µg RUTI® | |
| --- | --- | --- | --- | --- | --- | --- | --- | --- |
|  | Viral load (cps/mL) | Change from baseline | Viral load (cps/mL) | Change from baseline | Viral load (cps/mL) | Change from baseline | Viral load (cps/mL) | Change from baseline |
| Day | n / Mean  (range) | n / Mean (range) | n / Mean (range) | n / Mean (range) | n / Mean (range) | n / Mean (range) | n / Mean (range) | Mean (range) |
| Screening | (n=4) 3906.5  (450-12000) | - | (n=5) 6455.2 (52-27783) | - | (n=6) 85012.8 (1054-480000) | - | (n=6) 6407.5 (160-25176) | - |
| Day 28 | (n=4) 7551.5  (370-25000) | - | (n=6) 5372.5 (48-27805) | - | (n=7) 151238.6 (1109-970668) | - | (n=5) 12305.4 (710-54269) | - |
| Day 49 | (n=5) 8365.4 (1932-15110) | (n=4) 1155.3 (-12000-13270) | (n=5) 13500.6 (200-64293) | (n=5) 7063.2 (-900-36488) | (n=9 83071.2 (217-686573) | (n=7) -142546 (-965766-5116) | (n=6) 23370.5 (64-125250) | (n=5) 15726.4 (-852-70981) |
| Day 63 | (n=4) 7910.0 (1068-17000) | (n=4) 358.5 (-8000-7211) | (n=5) 11687.0 (368-53844) | (n=5) 5249.6 (-523-26039) | (n=7) 4471.4 (708-11616) | (n=7) -146767 (-969149-2700) | (n=5) 22367.4 (820-103442) | (n=5) 10062.0 (-852-49173) |
| Day 84  (Follow-up) | (n=6) 1964.2 (53-5225) | (n=4) -4634.3 (-19775-787) | (n=4) 708.8 (404-1157) | (n=4) -386.8 (-1539-139) | (n=8) 5385.4 (47-12910) | (n=7) -145091 (-969791-10728) | (n=5) 11923.8 (528-50114) | (n=5) -381.6 (-4155-4566) |

HIV=Human Immunodeficiency Virus; n = number of subjects in each treatment group with measurements at each time point
