# Supplementary material for: Safety, Tolerability, and Immunogenicity of the Novel Antituberculous Vaccine RUTI: Randomized, Placebo-Controlled Phase II Clinical Trial in Patients with Latent Tuberculosis Infection
Source: PLoS One. 2014 Feb 26;9(2):e89612. doi: 10.1371/journal.pone.0089612 (PMC3935928; doi:10.1371/journal.pone.0089612)
Supplement: Table S3 — Mean CD4 by treatment and change from baseline by time point, treatment and HIV-status. (DOC) [file pone.0089612.s004.doc]

**Table S3. Mean CD4 by treatment and change from baseline by time point, treatment and HIV-status**

| **Day/Observation/ Intensity** | **Placebo** |  | **5 µg RUTI®** |  | **25 µg RUTI®** |  | **50 µg RUTI®** |  |
| --- | --- | --- | --- | --- | --- | --- | --- | --- |
|  | **CD4 (cells/mm^3^)** | **Change from baseline** | **CD4 (cells/mm^3^)** | **Change from baseline** | **CD4 (cells/mm^3^)** | **Change from baseline** | **CD4 (cells/mm^3^)** | **Change from baseline** |
| **Day** | **(n) / Mean (range)** | **(n) / Mean (range)** | **(n) / Mean (range)** | **(n) / Mean (range)** | **(n) / Mean (range)** | **(n) / Mean (range)** | **(n) / Mean (range)** | **(n) / Mean (range)** |
| Screening | (n=12) | - | (n=11) | - | (n=12) | - | (n=12) | - |
|  | 578.9 |  | 615.4 |  | 462.3 |  | 587.7 |  |
|  | (398-877) |  | (358-1441) |  | (356-604) |  | (381-871) |  |
| Day 28 | (n=12) | - | (n=11) | - | (n=12) | - | (n=12) | - |
|  | 635.1 |  | 646.5 |  | 501.2 |  | 573.6 |  |
|  | (398-961) |  | (337-1398) |  | (340-753) |  | (365-1009) |  |
| Day 49 | (n=12) | (n=12) | (n=11) | (n=11) | (n=12) | (n=12) | (n=12) | (n=12) |
|  | 652.6 | 17.5 | 648.1 | 1.6 | 482.9 | -18.3 | 565.7 | -7.9 |
|  | (250-1134) | (-229-489) | (336-1409) | (-206-262) | (341-657) | (-234-143) | (369-989) | (-316-187) |
| Day 63 | (n=12) | (n=12) | (n=11) | (n=11) | (n=12) | (n=12) | (n=12) | (n=11) |
|  | 621.3 | -13.8 | 663.5 | 17.0 | 491.8 | -9.3 | 633.8 | 45.1 |
|  | (250-834) | (-153-227) | (344-1626) | (-291-360) | (254-736) | (-170-181) | (338-1194) | (-115-185) |
| Day 84 | (n=12) | (n=12) | (n=11) | (n=11) | (n=12) | (n=12) | (n=11) | (n=12) |
| (Follow-up) | 548.6 | -86.5 | 679.4 | 32.9 | 504.8 | 3.6 | 616.3 | 42.7 |
|  | (253-891) | (-378-117) | (239-1599) | (-179-201) | (216-720) | (-200-174) | (307-1096) | (-308-321) |

HIV=Human Immunodeficiency Virus; n = number of subjects in each treatment group with measurements at each time point
