# Supplementary material for: Safety, Tolerability, and Immunogenicity of the Novel Antituberculous Vaccine RUTI: Randomized, Placebo-Controlled Phase II Clinical Trial in Patients with Latent Tuberculosis Infection
Source: PLoS One. 2014 Feb 26;9(2):e89612. doi: 10.1371/journal.pone.0089612 (PMC3935928; doi:10.1371/journal.pone.0089612)
Supplement: Table S4 — Pain rating (Visual Analogue Scale) by time point, treatment and HIV-status. (DOC) [file pone.0089612.s005.doc]

**Table S4. Pain rating (Visual Analogue Scale) by time point, treatment and HIV-status**

|  | **Placebo** | | **5 µg RUTI®** | | **25 µg RUTI®** | | **50 µg RUTI®** | |
| --- | --- | --- | --- | --- | --- | --- | --- | --- |
| **Day/Observation/ Intensity** | **HIV- (n=12)** | **HIV+ (n=12)** | **HIV- (n=12)** | **HIV+ (n=11)** | **HIV- (n=12)** | **HIV+ (n=12)** | **HIV- (n=12)** | **HIV+ (n=12)** |
|  | **Mean** | **Mean** | **Mean** | **Mean** | **Mean** | **Mean** | **Mean** | **Mean** |
|  | **(min - max)** | **(min - max)** | **(min - max)** | **(min - max)** | **(min - max)** | **(min - max)** | **(min - max)** | **(min - max)** |
| Day 28 | 1.8 (0 – 9) | 0.4 (0 – 3) | 6.7 (0 – 38) | 0.8 (0 – 6) | 4.8 (0 - 50) | 0.8 (0 – 4) | 5.9 (0 – 34) | 0.9 (0 – 6) |
| Day 29 | 0.8 (0 – 6) | 0.3 (0 – 2) | 3.0 (0 - 25) | 4.4 (0 - 23) | 1.9 (0 – 14) | 5.2 (0 – 32) | 13.1 (0 - 53) | 10.5 (0 – 43) |
| Day 31 | 0.5 (0 – 5) | 0.4 (0 – 5) | 4.8 (0 – 43) | 10.2 (0 – 71) | 7.4 (0 – 33) | 13.3 (0 – 42) | 18.3 (0 – 63) | 21.3 (0 – 63) |
| Day 35 | 0.4 (0 – 5) | 0.2 (0 – 2) | 3.8 (0 – 31) | 1.9 (0 – 10) | 0.4 (0 - 2) | 7.7 (0 – 54) | 0.5 (0 – 2) | 2.0 (0 – 12) |
| Day 49 | 0 (0 – 0) | 0.1 (0 – 1) | 0.2 (0 - 1) | 1.2 (0 – 7) | 2.8 (0 – 27) | 8.3 (0 – 93) | 4.3 (0 – 49) | 3.2 (0 – 21) |
| Day 56 pre‑inoculation | 0 (0 – 0) | 0 (0 – 0) | 0.4 (0 – 5) | 1.1 (0 – 9) | 0.1 (0 – 1) | 5.7 (0 – 47) | 1.8 (0 – 18) | 0.8 (0 – 6) |
| Day 56 post‑inoculation | 0.6 (0 – 4) | 0.1 (0 – 1) | 1.1 (0 – 11) | 8.5 (0 - 82) | 0.5 (0 – 5) | 0.8 (0 – 4) | 0.5 (0 – 5) | 0.1 (0 – 1) |
| Day 57 | 0.1 (0 – 1) | 0 (0 – 0) | 3.8 (0 – 27) | 3.4 (0 – 18) | 3.5 (0 – 34) | 4.8 (0 – 20) | 16.3 (0 – 48) | 5.5 (0 – 31) |
| Day 59 | 4.9 (0 – 30) | 0 (0 – 0) | 10.9 (0 – 70) | 4.7 (0 – 20) | 14.9 (0 – 57) | 15.4 (0 – 56) | 21.4 (0 – 76) | 12.5 (0 – 40) |
| Day 63 | 0.1 (0 – 1) | 0 (0 – 0) | 2.8 (0 – 29) | 2.4 (0 – 15) | 0.2 (0 – 2) | 10 8 (0 – 94) | 2.7 (0 – 9) | 3.6 (0 – 37) |
| Day 84 (Follow-up) | 0 (0 – 0) | 0 (0 – 0) | 0.6 (0 – 7) | 1.0 (0 – 9) | 2.2 (0 – 23) | 3.8 (0 – 31) | 0.9 (0 – 7) | 3.1 (0 – 15) |

n=number of subjects; HIV=Human Immunodeficiency Virus; max=maximum; min=minimum

VAS – Visual Analogue Scale 0 to 100
