# Supplementary material for: Safety, Tolerability, and Immunogenicity of the Novel Antituberculous Vaccine RUTI: Randomized, Placebo-Controlled Phase II Clinical Trial in Patients with Latent Tuberculosis Infection
Source: PLoS One. 2014 Feb 26;9(2):e89612. doi: 10.1371/journal.pone.0089612 (PMC3935928; doi:10.1371/journal.pone.0089612)
Supplement: Protocol S1 — Trial Protocol. (PDF) [file pone.0089612.s007.pdf]

Clinical Trial Protocol

Double-Blind, Randomized, Placebo-Controlled Phase II Clinical Trial to Investigate the Safety, Tolerability, and Immunogenicity of the Novel Antituberculous Vaccine RUTI® Following One Month of Isoniazid Treatment in Subjects with Latent Tuberculosis Infection

**Sponsor:**	**ARCHIVEL FARMA, S.L.**<br>C/ Fogars de Tordera, 61<br>Pol. Ind. Bonavista<br>ES-08915 Badalona, Catalonia<br>Spain<br>Tel: 93.497.24.56<br>Fax: 93.497.24.57<br>archivel@archivelfarma.com
**Clinical Research Organization:**	PAREXEL International
**Principal/Coordinating Investigators:**	Dr AS Nell (Bloemfontein)<br>Dr DS Kruger (George)<br>Dr R McPherson (Port Elizabeth)
**PAREXEL Study No.:**	106010
**Sponsor Protocol No.:**	RUTISAPH2
**IMP Name:**	RUTI® Antituberculous Vaccine
**Development Phase:**	Phase II
**Date of Protocol:**	16 September 2009 (Final Version 1.0)<br>15 December 2009 (Final Version 1.0 according to Amendment No. 1)<br>03 March 2010 (Final Version 2.0 according to Amendment No. 2)<br>22 July 2010 (Final Version 3.0 according to Amendment No. 3)<br>06 October 2010 (Final version 4.0 according to Amendment No. 4)

This clinical trial will be conducted according to the protocol and in compliance with Good Clinical Practice (GCP) (1), with the Declaration of Helsinki (2) and with other applicable regulatory requirements.

Confidentiality Statement

This document contains confidential information of ARCHIVEL FARMA, S.L. Do not copy or distribute without written permission from the Sponsor.

SIGNATURE PAGE

Declaration of Sponsor or Responsible Medical Expert

Protocol Title: Double-Blind, Randomized, Placebo-Controlled Phase II Clinical Trial to Investigate the Safety, Tolerability, and Immunogenicity of the Novel Antituberculous Vaccine RUTI® Following One Month of Isoniazid Treatment in Subjects with Latent Tuberculosis Infection

This clinical trial protocol was subjected to critical review. The information it contains is consistent with current knowledge of the risks and benefits of the investigational medicinal product, as well as with the moral, ethical and scientific principles governing clinical research as set out in the Declaration of Helsinki, and the guidelines on Good Clinical Practice applicable to this clinical trial.

Sponsor Signatory/Responsible Medical Expert

Prof Pere-Joan Cardona	Date
(M.D., Ph.D.)	

SIGNATURE PAGE

Declaration of Disease Authority and Medical Advisor

Protocol Title: Double-Blind, Randomized, Placebo-Controlled Phase II Clinical Trial to Investigate the Safety, Tolerability, and Immunogenicity of the Novel Antituberculous Vaccine RUTI® Following One Month of Isoniazid Treatment in Subjects with Latent Tuberculosis Infection

This clinical trial protocol was subjected to critical review. The information it contains is consistent with current knowledge of the risks and benefits of the investigational medicinal product, as well as with the moral, ethical and scientific principles governing clinical research as set out in the Declaration of Helsinki, and the guidelines on Good Clinical Practice applicable to this clinical trial.

Disease Authority and Medical Advisor

Prof Gavin J Churchyard	Date

(MBBCh, M.Med, FCP, PhD)

SIGNATURE PAGE

Declaration of the Biostatistician

Protocol Title: Double-Blind, Randomized, Placebo-Controlled Phase II Clinical Trial to Investigate the Safety, Tolerability, and Immunogenicity of the Novel Antituberculous Vaccine RUTI® Following One Month of Isoniazid Treatment in Subjects with Latent Tuberculosis Infection

This clinical trial protocol was subjected to critical review and has been released by the Sponsor. The information it contains is consistent with current risk and benefit evaluation of the investigational medicinal product, as well as with the moral, ethical and scientific principles governing clinical research as set out in the Declaration of Helsinki, and the guidelines on Good Clinical Practice applicable to this clinical trial. This clinical trial involves research.

Biostatistician

CJ Bester<br>Manager Biostatistics	Date

PAREXEL Bloemfontein

SIGNATURE PAGE

Declaration of the Principal/Coordinating Investigator

Protocol Title: Double-Blind, Randomized, Placebo-Controlled Phase II Clinical Trial to Investigate the Safety, Tolerability, and Immunogenicity of the Novel Antituberculous Vaccine RUTI® Following One Month of Isoniazid Treatment in Subjects with Latent Tuberculosis Infection

This clinical trial protocol was subjected to critical review and has been released by the Sponsor. The information it contains is consistent with current risk and benefit evaluation of the investigational medicinal product, as well as with the moral, ethical and scientific principles governing clinical research as set out in the Declaration of Helsinki, and the guidelines on Good Clinical Practice applicable to this clinical trial. This clinical trial involves research.

Principal/Coordinating Investigator

____

Name (Print)

____ ________________________________

Signature Date

PROTOCOL SYNOPSIS

**Protocol Title:**	Double-Blind, Randomized, Placebo-Controlled Phase II Clinical Trial to Investigate the Safety, Tolerability, and Immunogenicity of the Novel Antituberculous Vaccine RUTI® Following One Month of Isoniazid Treatment in Subjects with Latent Tuberculosis Infection
**Trial Numbers:**	PAREXEL Study No.: 106010<br>Sponsor Protocol No.: RUTISAPH2
**Development Phase:**	Phase II
**Sponsor:**	ARCHIVEL FARMA, S.L.
**Principal/Coordinating Investigators:**	Dr AS Nell (Bloemfontein)<br>Dr DS Kruger (George)<br>Dr R McPherson (Port Elizabeth)
**Trial Centres:**	PAREXEL International<br>PAREXEL Bloemfontein<br>Kampuslaan Suid<br>Campus of the University of the Free State<br>9301 Bloemfontein<br>South Africa<br>PAREXEL George<br>101 Windsor Park<br>1 Herrie Street<br>6529 George<br>South Africa<br>PAREXEL Port Elizabeth<br>63 Worraker Road<br>Newton Park<br>6045 Port Elizabeth<br>South Africa
**Trial Objective(s):**	• To evaluate the safety and tolerability of three different doses (5, 25 & 50 µg of FCMtb) of one formulation of the novel antituberculous vaccine RUTI® in subjects with latent tuberculosis infection (LTBI), compared to placebo.<br>• To evaluate the immunogenicity of three different doses (5, 25 & 50 µg of FCMtb) of one formulation of the novel antituberculous vaccine RUTI® in subjects with LTBI, compared to placebo.
**Trial Design:**	Double-blind, randomized, placebo-controlled phase II trial where the vaccine or placebo, dose randomised to each volunteer, will be administered twice (28 days apart) following one month of pre-treatment with isoniazid (INH: 300mg/day).
**Number of Subjects:**	Ninety-six (96) subjects will be evaluated.
**Trial Population:**	Latent tuberculosis infected subjects, from 18 up to 50 years, HIV+ and HIV-, without any disease that could compromise the evaluation of the response to the vaccine, or could increase the risk of having an adverse outcome from participating in this protocol.
**Methodology:**	This is a safety and tolerability trial (Phase II) where three doses will be tested in order to assess their tolerability as a first end-point and will end after three months from the beginning of the assay.

Subjects will be included randomly in different treatment groups as figured below:

	n	LTBI/HIV-	n	LTBI/HIV+
Phase II	12	RUTI® (5µg of FCMTb)	12	RUTI® (5µg of FCMTb)
	12	RUTI® (25µg of FCMTb)	12	RUTI® (25µg of FCMTb)
	12	RUTI® (50µg of FCMTb)	12	RUTI® (50µg of FCMTb)
	12	placebo	12	placebo

Tolerability Evaluation: Toxicity will be evaluated for the subjects visiting the site according to the flow chart; physical examinations (including vital signs measurement) will be performed, as well as local inoculation inspection, and laboratory sampling for safety testing. Chest computed tomography will also be performed in order to detect any Koch reaction at hilar lymph nodes. Adverse events will be recorded after questioning the subjects at each visit, as communicated spontaneously by the subjects, or as observed by the investigators or their collaborators.

Immunogenicity Evaluation: Samples for immunogenicity testing will be obtained according to the Flow Chart shown in Section 3.2 of this Protocol. Cellular mediated immunity will be monitored using ELISPOT and ELISA techniques. IFN-γ Spot Forming Units will be measured in the subjects' peripheral blood after stimulation with up to five stimuli (ESAT 6, 16 kDa, Ag85B and 38 kDa *M. tuberculosis* antigens, and PPD) for 18 hours. A long-term assay interferon (WHO assay) will be performed by stimulating the whole blood for seven days with PPD. One commercial TIGRA (TSPOT TB assay) will also be performed. Peripheral blood mononuclear cells will be frozen for future immune assays if required. Sera will also be frozen to be further tested for the antibody-mediated immunity against *M.tuberculosis* antigens

Investigational Medicinal Products:

Name:	RUTI® Antituberculous Vaccine
Pharmaceutical Form:	Lyophilised powder for suspension for injection
Administration Form:	Suspension for injection
Administration route:	Subcutaneous route
Dose and Dosage Regimen:	Subjects randomized to receive either 5 µg, 25 µg or 50 µg doses will be injected twice (28 days apart).

Name:	Matching RUTI® Placebo
Pharmaceutical Form:	Lyophilised powder for suspension for injection
Administration Form:	Suspension for injection
Administration route:	Subcutaneous route
Dose and Dosage Regimen:	Subjects randomized to receive placebo will be injected twice (28 days apart).

Duration of the Subject Exposure to the Treatments: Two administrations, 28 days apart.

Total Duration of the Trial for Subject: Each subject will be screened within the 28 days period prior to Visit 1 (Day 0). Should the screening period be longer than 28 days subjects may be re-screened after agreement between the sponsor and the investigator. After inclusion into the trial, each subject will remain in the trial for a total of 84 days. The treatment and follow-up periods for each subject is as follows:

- Twenty eight days of pre-treatment with INH
- Further 28 days of RUTI® treatment (2 administrations, 28 days apart)
- Follow-up period of 28 days

**Statistical Analysis:**	Adverse events will be listed and summarized by system organ class and preferred terms for each treatment group using descriptive statistics. Tolerability assessments will be listed per patient and summarized using frequency counts or descriptive statistics, as appropriate. Physical examination results will be summarized using frequency counts and shift tables. Concomitant medication will be coded using the World Health Organization Drug Reference List (WHO-DRL) and Anatomic Therapeutic Chemical (ATC) classification, listed per patient and summarized per treatment group.<br>Vital signs, laboratory parameters and ECG parameters will be listed (both absolute and change from baseline results) and summarized per treatment group and visit, for absolute and change from baseline results, using descriptive statistics.<br>Clinical history results, TST and QuantiFeron, chest X-ray, chest CT scan, CD4 counts, and viral load results will be listed.<br>The immunogenicity parameters [IFN-γ Spot Forming Units in peripheral blood mononuclear cells (PBMCs), IFN-γ concentration in whole blood and antibody concentration in sera] will be listed per patient and summarized using descriptive statistics by treatment group and visit. The difference between treatments effect will be studied by comparing the measurement of the immunogenicity parameters. An analysis of covariance (ANCOVA) will be used, with treatment as main effect and the baseline value as covariate. The data and comparisons between the treatments will be described by the least square means adjusted in accordance to the ANCOVA analysis
**Anticipated Trial Schedule:**	First subject's first visit: by end of June 2010<br>Last subject's last visit: November 2010<br>Preliminary results: February 2011<br>Draft report: March 2011

LIST OF TRIAL STAFF

**Sponsor:**	ARCHIVEL FARMA, S.L.<br>C/ Fogars de Tordera, 61<br>Pol. Ind. Bonavista<br>ES-08915 Badalona, Catalonia<br>Spain<br>T: 93.497.24.56<br>F: 93.497.24.57<br>archivel@archivelfarma.com<br><br>**Sponsor Medical Expert:**<br>Prof. Pere-Joan Cardona, M.D., Ph.D.<br>C/ Fogars de Tordera, 61<br>Pol. Ind. Bonavista<br>ES-08915 Badalona, Catalonia<br>SPAIN<br>Phone no. +34 618 780 698<br>Fax no.: +34 93 497 86 54<br>e-mail address: pj.cardona@gmail.com
**Disease Authority and Medical advisor:**	Prof. Gavin Churchyard MBChB, MMED(Internal Medicine), FCP(SA), PhD.<br>CEO: The Aurum Institute<br>Phone no. +27 (0) 11 484 8844<br>Fax no. +27 (0) 11 484 4682<br>Cell: +27 (0)82 556 5536<br>E-mail address: gchurchyard@auruminstitute.org
**Principal/Coordinating Investigators:**	Dr AS Nell (Bloemfontein)<br>Dr DS Kruger (George)<br>Dr R McPherson (Port Elizabeth)
**Contract Research Organization:**	PAREXEL International (South Africa)
**Clinical Laboratory:**	**Safety:**<br>Morné Wiese<br>Bioanalytical Services Division<br>PAREXEL Bloemfontein<br>Kampuslaan Suid, Campus of the University of the Free State, 9301 Bloemfontein, South Africa<br>Tel: +27 51 410 3197<br>Fax: +27 51 410 3195<br>Email: morne.wiese@parexel.com<br><br>Marlene Fourie<br>Drs Laing, Soldin and Venter (Pathcare)<br>1 Gloucester Lane, PO Box 815, George, 6530, South Africa<br>Tel: +27 44 803 8200

Fax: +27 44 874 4077
Email: marlene.fourie@pathcare.co.za

Robert Gillmore
Ampath Laboratories
Suite 212, Netcare, Greenacres Hospital, CNR Rochelle and Cape Road, Greenacres, Port Elizabeth, 6045, South Africa
Tel: +27 41 363 2339
Fax: +27 41 363 2235
Email: gillmorer@ampath.co.za

Bioanalytical Laboratories:

Edda Zangenberg
Bioanalytical Services Division
PAREXEL Bloemfontein
Kampuslaan Suid, Campus of the University of the Free State, 9301 Bloemfontein, South Africa
Tel: +27 51 410 3178
Fax: +27 51 444 5969
Email: edda.zangenberg@parexel.com

Dr Justin J. Devine
Chief Medical Officer
Synexa Life Sciences
Tel: +27 21 9339581
Fax: +27 21 9319953
Email: jdevine@synexagroup.com

Robert Voogt (sputum culturing)
Dip Clin Path; PM - USB
Administrative Manager
PathCare Clinical Trials
Tel: +27 21 596 35 88
Fax: +27 21 596 37 10
Mobile: 082 807 0333
robertv@pathcare.co.za
www.pathcare.co.za

Pharmacy:

Pharmaceutical Services,
PAREXEL Bloemfontein
Kampuslaan Suid, Campus of the University of the Free State, 9301 Bloemfontein, South Africa
Tel: +27 51 410 3163
Fax: +27 51 444 5969
Email: +Bloemfontein-Pharmaceuticalservices@parexel.com

PAREXEL George
101 Windsor Park
1 Herrie Street
6529 George

South Africa
Tel: 044 803 0200 ~~044 884 1560~~ (Reception)
Fax: 044 884 1566
Email: maryke.reyneke@parexel.com

PAREXEL Port Elizabeth
63 Worraker Road
Newton Park
6045 Port Elizabeth
South Africa
Tel: 041 398 9700 (Reception)
Fax: 041 365 0231
Email: lorraine.nel@parexel.com

Statistics and Data Management:

CJ Bester
PAREXEL Bloemfontein
Kampuslaan Suid
Campus of the University of the Free State
9301 Bloemfontein
South Africa
Tel: +27 51 410 3129
Fax: +27 51 444 5969
Email: ina.bester@parexel.com

Clinical Data Coordinator:

J Dreyer
PAREXEL Bloemfontein
Kampuslaan Suid
Campus of the University of the Free State
9301 Bloemfontein
South Africa
Tel: +27 51 410 3130
Fax: +27 51 444 5969
Email: jaco.dreyer@parexel.com

TABLE OF CONTENTS

PROTOCOL SYNOPSIS 6
LIST OF TRIAL STAFF 9
TABLE OF CONTENTS 12
List of Tables 14
LIST OF ABBREVIATIONS 15
1. BACKGROUND INFORMATION 17
1.1 Name and Description of the Investigational Product 17
1.2 Summary of Findings from Non-Clinical Studies that Potentially Have Clinical Significance and from Clinical Trials that are Relevant to the Trial 17
1.3 Summary of the Known and Potential Risks and Benefits to Human Subjects 27
1.3.1 Potential Risks, Safety, Toxicity and Tolerance 27
1.3.2 Potential Benefits 31
1.4 Description of and Justification for the Route of Administration, Dosage, Dosage Regimen and Treatment Period 33
1.5 Description of the Population to be Studied 37
2. TRIAL OBJECTIVES AND PURPOSE 38
3. TRIAL DESIGN 39
3.1 Endpoints to be Measured During the Trial 39
3.2 Description of the Type/Design of Trial to be Conducted (Diagram of Trial Design, Procedures and Stages) 39
3.3 Randomization and Blinding 41
3.4 Description of the Trial Treatments and the Dosage and Dosage Regimen of the Investigational Product. Dosage Form, Packaging, and Labeling of the Investigational Product 43
3.5 Expected Duration of Subject Participation 45
3.6 Stopping Rules or Discontinuation Criteria for Individual Subjects, Parts of Trial and Entire Trial 45
3.7 Drug Accountability Procedures 46
3.8 Randomization and Maintenance of Trial Treatment Randomization Codes and Procedures for Breaking Codes 46
4. SELECTION AND WITHDRAWAL OF SUBJECTS 48
4.1 Inclusion Criteria 48
4.2 Exclusion Criteria 48
4.3 Subject Withdrawal Criteria 50

5. TREATMENT OF SUBJECTS 52
5.1 Treatment to be Administered (Product, Dose, Dosing Schedule, Route of Administration, Treatment Period, Follow-up Period for Subjects 52
5.2 Prohibitions and Restrictions. Medication(s)/Treatment(s) Permitted and not Permitted Before and/or During the Trial 54
5.3 Procedures for Monitoring Subject Compliance 54
6. ASSESSMENT OF IMMUNOGENICITY 55
6.1 Immunogenicity Parameters 55
6.2 Methods and Timing for Assessing, Recording and Analysing of Immunogenicity Parameters 55
7. ASSESSMENT OF SAFETY 56
7.1 Safety Parameters: Methods and Timing for Assessing, Recording and Analysing 56
7.2 Procedures for Eliciting Reports of and for Recording and Reporting Adverse Event and Intercurrent Illnesses 58
7.2.1 Adverse Event Definitions and Classifications 58
7.2.2 Adverse Event Reporting Procedures 60
7.3 Type and Duration of the Follow-up of Subjects after Adverse Events 61
8. ACTIVE TB SURVEILLANCE [9, 10, 11] 62
8.1 Diagnosis of Pulmonary TB 62
8.1.1 Clinical Features 62
8.1.2 Chest X-Ray in Diagnosis 62
8.1.3 Confirmatory Diagnosis by Smear Microscopy and Mycobacterial Culturing 63
8.2 Diagnosis of Extrapulmonary TB 63
9. STATISTICS (BIOMETRIC PLAN) 65
9.1 Data Management 65
9.2 Immunogenicity Data Handling 66
9.3 Statistical Methods 66
9.3.1 Safety/Tolerability Parameters 66
9.3.2 Immunogenicity Parameters 66
9.4 Interim Analysis 67
9.5 Trial Supervision and Monitoring 67
9.6 Number of Subjects Planned to be Enrolled (Sample Size) 68
9.7 Criteria for the Termination of the Trial 69
9.8 Procedure for Accounting for Missing, Unused, and Spurious Data 69
9.9 The Selection of Subjects to be Included in the Analyses 69
10. DIRECT ACCESS TO SOURCE DATA/DOCUMENTS 71
11. QUALITY CONTROL AND QUALITY ASSURANCE 71
11.1 Data Quality Control 71

11.2 Monitoring ... 71
12. ETHICS ... 73
12.1 Investigator Responsibilities ... 73
12.2 Independent Ethics Committee or Institutional Review Board (IE/IRB) ... 73
12.3 Informed Consent ... 74
12.4 Privacy of Personal Data ... 74
13. DATA HANDLING AND RECORD KEEPING ... 76
13.1 Case Report Form Completion ... 76
13.2 Record Retention ... 76
14. INSURANCE ... 78
15. PUBLICATION POLICY ... 79
16. REFERENCE LIST ... 80
16.1 Bibliography ... 81
17. APPENDICES ... 85
17.1 CDC Classification System for HIV Infection ... 85
17.2 Alcohol Intake Questions ... 87

List of Tables

Table 1 Systemic AEs. Number (%) of subjects reporting one or more treatment emergent AEs and number of AE occurrences by preferred term, possible or probable causality to the treatment and intensity. (O=Occurrences; S=Subjects) ... 30
Table 2 Local reactions and statistical differences between treatments groups ... 30
Table 3 Local AEs. Number (%) of subjects reporting one or more treatment-emergent AEs and number of AE occurrences by preferred term, possible or probable causality to the treatment and intensity. (O=Occurrences; S=Subjects) ... 31
Table 4 Groups involved in the 2 phases of the clinical trial ... 42
Table 5 The qualitative and quantitative composition per vial of RUTI® vaccine ... 43
Table 6 The concentration of each component per vial after reconstitution ... 43
Table 7 “Classical” and “atypical” chest X-ray patterns ... 62
Table 8 In the following tables we give the probabilities of detecting an adverse event between 0.1 to 10% and the exact confidence intervals for the whole set exposed to the vaccine (N=32) ... 68

LIST OF ABBREVIATIONS

AE	Adverse event
ALP	Alkaline phosphatase
ALT	Alanine aminotransferase
AST	Aspartate aminotransferase
ATC	Anatomic Therapeutic Chemical
BCG	Bacilli Calmette-Guérin
BDR	Blind data review
CK	Creatine kinase
CPT	Cell Preparation Tube ™
CRF	Case report form
CXR	Chest X-ray
DSMC	Data Safety Monitoring Committee
ECG	Electrocardiogram
ELISPOT	Enzyme-linked immunosorbent spot
ELISA	Enzyme-linked immunosorbent assay
EPTB	Extrapulmonary tuberculosis
FCMtb	Fragments of *Mycobacterium tuberculosis* cells
GCP	Good Clinical Practice
GGT	Gamma-glutamyl transpeptidase
GLP	Good Laboratory Practice
GMP	Good Manufacturing Practice
HIV	Human immunodeficiency virus
HR	Heart rate
IEC	Independent Ethics Committee
ICH	International Conference on Harmonisation
IFN-γ	Interferon gamma
INH	Isoniazid
IRB	Institutional Review Board
LTBI	Latent tuberculosis infection
MCH	Mean corpuscular haemoglobin
MCHC	Mean corpuscular haemoglobin concentration
MCV	Mean corpuscular volume
MedDRA	Medical Dictionary for Regulatory Activities

NALC	N-acetyl-L-cysteine
NOAEL	Non-observed adverse event level
PBMC	Peripheral blood mononuclear cell
PP	Per protocol
PPD	Protein purified derivative
PTB	Pulmonary tuberculosis
RBC	Red blood cell
SAE	Serious adverse event
SAP	Statistical analysis plan
SUSAR	Suspected unexpected serious adverse reaction
TB	Tuberculosis
TST	Tuberculin skin test
VAS	Visual analogue scale
WBC	White blood cell
WHO	World Health Organization
WHO-DRL	World Health Organization - Drug Reference List

1. BACKGROUND INFORMATION

ARCHIVEL FARMA, S.L. is developing RUTI® vaccine, a polyantigenic liposomal vaccine containing fragments of *Mycobacterium tuberculosis* cells (FCMtb) for the prevention of active tuberculosis (TB) in subjects with latent tuberculosis infection (LTBI). It triggered a strong, specific polyantigenic response in mice, guinea pigs, goats and minipigs after one month's pre-treatment with isoniazid (INH). Safety pharmacology studies (Irwin test and investigations in anaesthetised dogs) did not show any effect on the central nervous, cardiovascular or respiratory systems, nor have autoimmune reactions been detected. The only relevant adverse events (AE) in single- and repeated-dose toxicity studies were local reactions at the injection site. Because of these encouraging results, development of RUTI® vaccine in humans has started, and one Phase I study to assess safety, tolerability, and immunogenicity in healthy volunteers has been completed.

Accordingly with the clinical developmental plan it is necessary to proceed with the trial of safety, tolerability, and immunogenicity in LTBI subjects.

1.1 Name and Description of the Investigational Product

The trial drug is RUTI® vaccine, a polyantigenic vaccine made from fragmented *M. tuberculosis* bacilli grown in stress, detoxified and liposomed, designed to fill the immunological gap left by short-term therapy against LTBI. Administered after a short term therapy with INH (one month), it is able to boost the immunological response, mostly focused against growing bacilli (Figure 1~~Figure 1~~), as well as triggering a new one against structural and "stress induced" antigens, which are present in latent bacilli. As a result, RUTI® achieves to control the reactivation of the *M. tuberculosis* bacilli in different experimental models (Figure 1~~Figure 1~~).

1.2 Summary of Findings from Non-Clinical Studies that Potentially Have Clinical Significance and from Clinical Trials that are Relevant to the Trial

Experience from studies in experimental animal models

The RUTI® vaccine has shown immunogenic and bactericidal efficacy in a wide range of mice strains, as well as in larger animals (guinea-pig, goat and mini-pig models) when administered after a short period of antimicrobial therapy with INH.

Inoculation with RUTI® in infected mice treated with short-period chemotherapy induces a strong immunological Th1/Th2/Th3 response against 13 *M. tuberculosis* antigens, which is able to induce marked accumulation (10-fold) of protein purified derivative (PPD)-specific IFN-γ-producing CD4 and CD8+ T cells in infected lungs (Figure 2~~Figure 2~~) (3). Interestingly, immunotherapy with Bacilli Calmette-Guérin (BCG) increased only the recruitment of CD4+ T cells.

Effectiveness has been demonstrated in a wide range of mice strains, as well as in larger animals such as guinea-pigs, goats and mini-pigs.

Using three different experimental models (mice infected intraperitoneally or by low dose aerosol, and aerosol-infected guinea pig) RUTI® treated animals showed the lowest bacillary load. RUTI® also decreased the percentage of pulmonary granulomatous infiltration in mice, as well as the pathology scoring in the guinea-pig model, where it is especially difficult to demonstrate a bactericidal effect because control of the bacillary concentration is better than in mice (Figure 3~~Figure 3~~).

IFN-γ production was detected through intracellular staining, enzyme-linked immunosorbent spot (ELISPOT) and enzyme-linked immunosorbent assay (ELISA) after being stimulated with a wide range of *M. tuberculosis* specific structural and secreted antigens. Data demonstrates that the difference between the therapeutic administration of BCG and RUTI® resides in the strong activation of IFN-γ+ CD4+ and CD8+ cells against PPD, ESAT-6 and Ag85B; while in comparison to mice only treated with a short-term chemotherapy, the difference also implies other *M. tuberculosis* structural antigens like 16, 19, 38 and 40 kDa and hsp65. The results show that RUTI® plays a protective role in the control of LTBI by triggering a Th1-specific immune response against structural and growing related antigens that reduces both the bacillary load and the pulmonary pathology.

Long progression experiments (i.e. 24 weeks evolution) show no difference from controls in bacillary count, but survival and the extent of pathological pulmonary changes were better in RUTI® treated animals.

This effect was also seen in *M. caprae*-infected goats in a field study.

Short-period chemotherapy markedly reduced both pulmonary and extrapulmonary affectation, but only treatment with Qx plus RUTI® decreased the extrapulmonary dissemination and the pathology scores for the hiliar lymph nodes, a key indicator for efficacy in humans. Following up the animals included in this study monitoring the production of IFN-γ. in the peripheral blood

induced by the ex-vivo stimulation with ESAT 6, also showed a marked increase on those RUTI® treated ones (Figure 4~~Figure 4~~).

RUTI® effectiveness was also demonstrated in the mini-pig experimental model. After infecting 18 *spf* mini-pigs with a low-dose *M. tuberculosis* (by intrapulmonar route), three treatment groups (six animals each) were specified: a non-treated group, INH treated and treated with INH plus two RUTI® inoculations. Samples from each animal were extracted on 9 time points during the 21-week follow up for the immunological study. The animals were then sacrificed and samples extracted for histological and bacteriological exam.

Results of this study showed that RUTI® triggered the specific immune response, increasing the effector T-cells secreting IFN-γ against specific *M. tuberculosis* antigens, also controlling intra and extrapulmonar dissemination, decreasing the tuberculous lesions number and the affected area (Figure 5~~Figure 5~~ and Figure 6~~Figure 6~~).

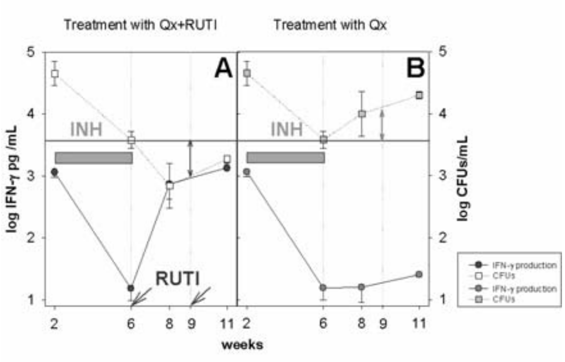

Figure 1 **Control of the bacillary load induced by the inoculation of RUTI® after a short term chemotherapy in a model of LTBI in mice, induced by the intraperitoneal inoculation of *M. tuberculosis*. Picture A shows both control of reactivation and bactericidal activity, while the bacilli reactivates if RUTI® is not administrated (Picture B).**

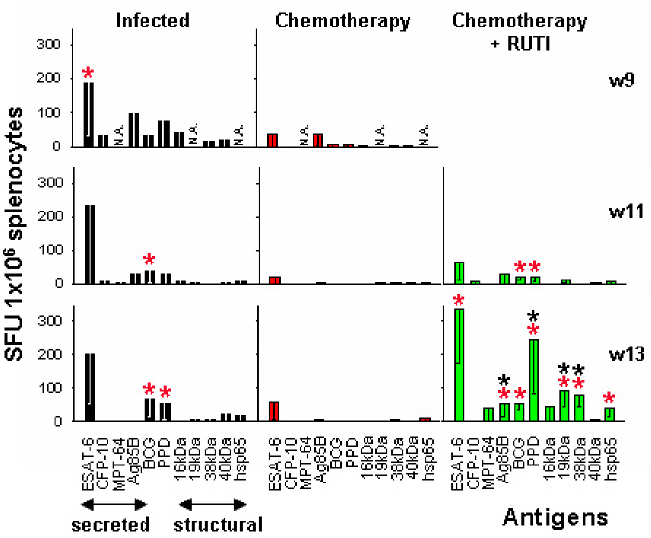

Figure 2 **Cellular immunity in the experimental model of LTBI induced by I.P inoculation. Activated antigen-specific IFN-γ secreting cells from spleen of intraperitoneally-infected mice. After infection, mice were treated with INH/RIF from weeks 3-9 (in red) and with two subcutaneous inoculations of RUTI® at weeks nine and 11 (in green). Untreated mice are shown in black. Data represent the mean and standard deviation. Differences were significant when marked with * for $p < 0.05$ (t-test) and expressed in the experimental group with the higher value. The colour of the asterisk shows the group with lower values to which the comparison was significant. N.A. means not assayed.**

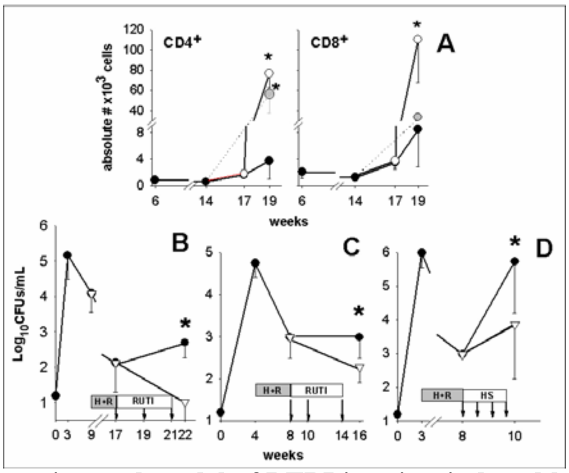

Figure 3 **Experimental model of LTBI in mice, induced by low dose aerosol. Picture A shows the production of IFN-γ + lung cells reacting towards PPD; Picture B shows the control of the bacillary load induced by the inoculation of RUTI®. The same effect can be induced in infected guinea pigs, after just four weeks INH treatment (Picture C). Picture D shows the effect of immune serum induced in immunocompetent mice after INH + RUTI® treatment, in SCID mice treated with R+H for five weeks. Passive serumtherapy lead to a significant control of the reactivation. Legends: H+R= chemotherapy with INH+rifapentine. Groups: infected plus H+R (black); H+R plus RUTI® Tt (white); H+R plus BCG (gray).**

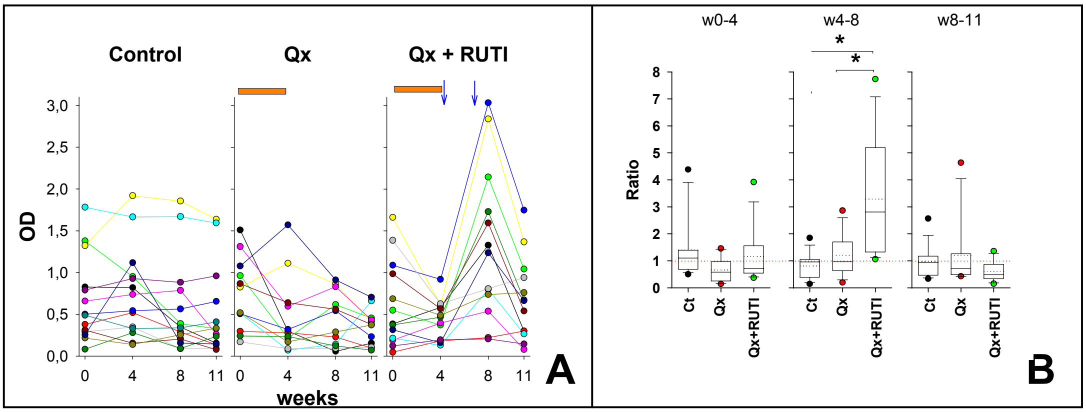

Figure 4 **Field study in *M. bovis* infected goats. Production of IFN-γ after exvivo stimulation of peripheral blood with ESAT-6. Picture A reflects individual monitoring of each animal. Picture B shows the ratios between weeks to check the tendency of the values (<1 decrease; >1 increase). Orange box shows the chemotherapy period and blue arrows RUTI® inoculation.**

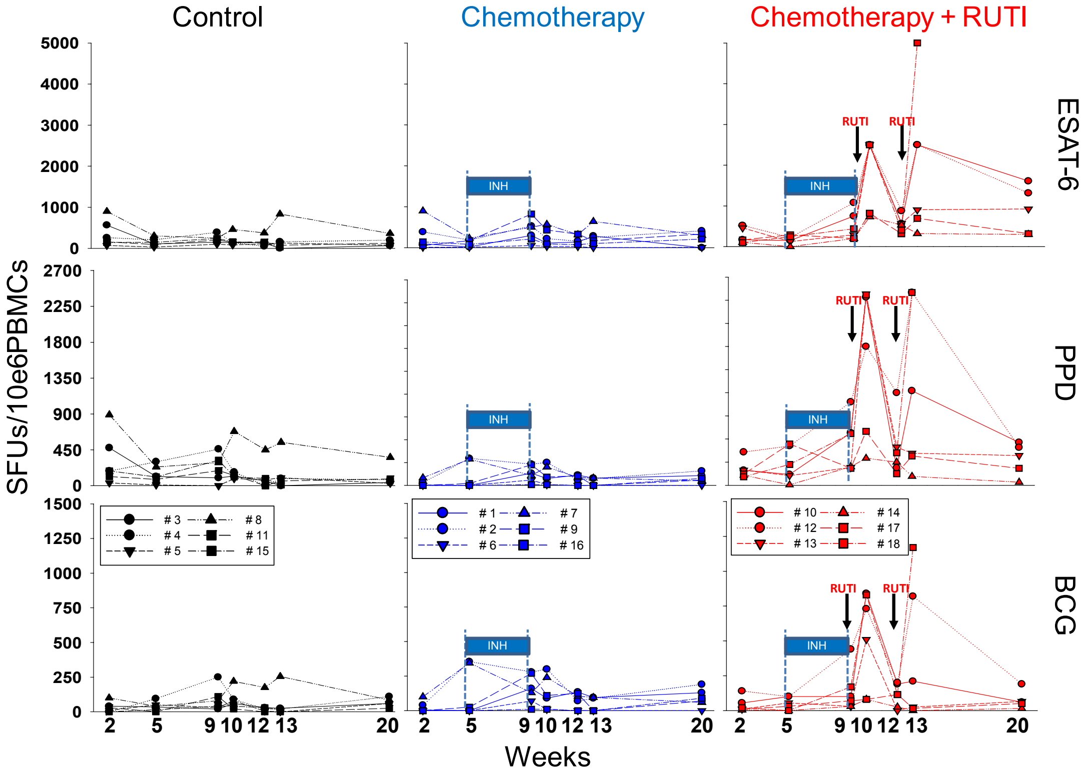

Figure 5 **Experimental model in mini-pigs. ELISPOT results showing IFN-γ producing cells after ex vivo stimulation of PBMCs with PPD, ESAT-6 or BCG. IFN-γ producing cells increase during chemotherapy because bacilli are killed. Tuberculous antigens can thus be acquired and presented by the antigen presenting cells. When chemotherapy is finished, the response decreases because of the lower bacterial load due to the isoniazid treatment. In contrast, an increase in IFN-γ secretion is seen after every RUTI® inoculation in vaccinated animals.**

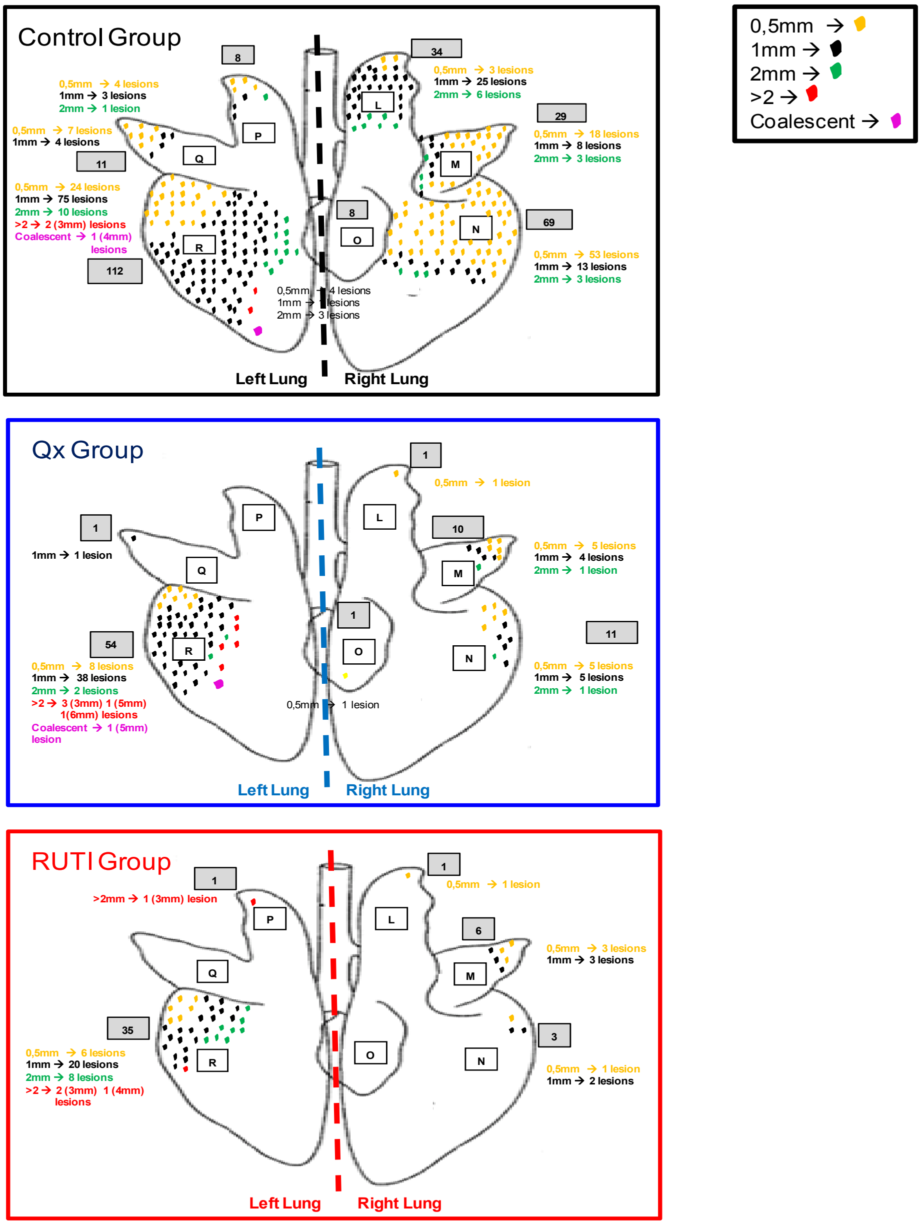

Figure 6 **Histopathological analysis of mini-pig lungs per treatment group. The RUTI® group has fewer lesions in the entire lung and with less dissemination to the right lung (animals were infected through the left lung). The sum of lesions for the six animals in each group in each lobe is shown; the total number of lesions in each lobe is shown in the grey squares.**

The Phase I Clinical Trial Background

The Phase I clinical trial to test the safety and immunogenicity of RUTI® when administered twice (28 days apart) to healthy volunteers began on 23 April 2007. A total of four increasing doses of RUTI® and placebo were tested (the dose was escalated after satisfactory tolerance of the previous dose): 5, 25, 100 and 200 µg FCMtb. Each group consisted of six volunteers, four of whom received RUTI® and two received placebo.

The main goal of the immunological testing was to determine the IFN-γ production by blood cells because this is the most potent tool the body has to fight *M. tuberculosis* infection. T cell interferon-γ-release assay (TIGRA) in peripheral blood after stimulation with up to eight *M. tuberculosis* antigens (ESAT-6, CFP-10, 16 kDa, 19 kDa, Ag85B, MPT-64, 38 kDa and hsp65), PPD and BCG by ELISPOT and ELISA techniques were included in this study. Cellular and humoral response was monitored with a wide range of assays, in order to characterize this response but also to determine the best test (the most reliable, but also the most logistically available) to monitor the Phase II clinical trial. A summary of the results have been added below as Figure 7~~Figure 7~~ and Figure 8~~Figure 8~~.

This Phase I clinical trial was also the first approach to study the mechanisms of action of the vaccine RUTI® in humans. A wide range of assays were planned, in order to screen any immunological response enhanced by the vaccination as well as to determine which assays should be performed in future clinical trials. The results obtained showed us that a specific immunological response was triggered by the vaccination, which sometimes were significantly different from the one obtained in the placebo-vaccinees, and could also be detected by the commercial ELISPOT and ELISA assays designed to diagnose LTBI. A general overview to the results shows us that there exists a dose-related tendency for most of the assays conducted. Highest doses (especially a dose of 200 µg of FCMtb) showed a maximum of highest results, evidenced in terms of the ELISPOT and ELISA techniques to determine the specific IFN-γ secretion. Even though, a dose of 25 µg of FCMtb achieved a polyantigenic response, wider than a dose of 100 µg of FCMtb and similar to the one reached by dose of 200 µg of FCMtb, a fact easily recognizable in the ELISPOT assays. Moreover, even if in the ELISA studies (less sensible and specific than ELISPOT) doses of 100 and 200 µg of FCMtb showed the highest results, a dose of 25 µg of FCMtb showed a discrete but clear increase of the IFN-γ secretion attributable to the vaccination. The most important problem of the ELISA results is the high specific results for some of the antigens obtained by the placebo group in latest time points. Those results are meant to be due to some of the individuals that conferred this group. In spite of

this fact, the superiority of RUTI® compared to placebo is clear in terms of immunological responses. In terms of the stimuli used to determine the specific-IFN-γ mediated cellular response, not all the antigens showed the same evolution after the vaccination. PPD and BCG vaccine were included in both tests because their nature, as they are both extracts of *M. tuberculosis* and *M. bovis* respectively, both containing several mycobacterial antigens. That is the reason for the highest results obtained in both assays for these stimuli, and a clearer RUTI® dose-response. Meanwhile, the single antigens were expected to trigger weaker responses after the vaccination, as RUTI® is an extract of *M. tuberculosis* containing several antigens but every one in low concentrations. The single antigens that showed the highest responses for RUTI® doses in both assays were ESAT-6, 85B, 38kDa, 19kDa, hsp65 and 16kDa (more discrete). CFP-10 and MPT64 showed weak responses compared to the other antigens tested. This clearly suggests that a strong polyantigenic response is achieved with the RUTI® vaccination against *M. tuberculosis* antigens secreted by active and latent bacilli, and will help in order to select the more antigenic antigens to test the RUTI® vaccine in future clinical trials.

All the responses obtained tended to be increased after the second inoculation, and then tended to decrease with a later increase at latest time points of the study. The high and maintained responses obtained in the memory assay, where the production of IFN-γ secretion by the Memory T cell subset is evaluated, reinforced this idea. All this suggests the ability of the vaccination to induce long-lasting protection, even if it would be interesting to follow-up the volunteers in the future to exactly determine how long this protection can last. In terms of % of subsets of T cells, RUTI® vaccination enhanced the T cell populations as was expected from previous studies conducted in animal models during the preclinical development. All the RUTI® doses had at least at one time point mycobactericidal activity after incubating the volunteers' whole blood with *M. tuberculosis* for 72 or 96 hours), while the placebo-vaccinees' whole blood did not. But this ability did not last for long, generally reverting, and even if this assay could be useful, it is not recommended being logistically difficult.

Humoral immunological response was evaluated by detection of specific antibodies against several *M. tuberculosis* antigens, showing a dose-response increase of IgM against all the antigens even if the differences were not strong enough to be statistically significant.

The conclusions of the trial were that the vaccination with RUTI® demonstrated to be safe and well-tolerated when administered to healthy volunteers, even if the lowest doses (5 and 25 µg of FCMtb) showed the best risk/benefit relation; and to trigger a specific immunological response against *M. tuberculosis* in healthy subjects, compared to placebo.

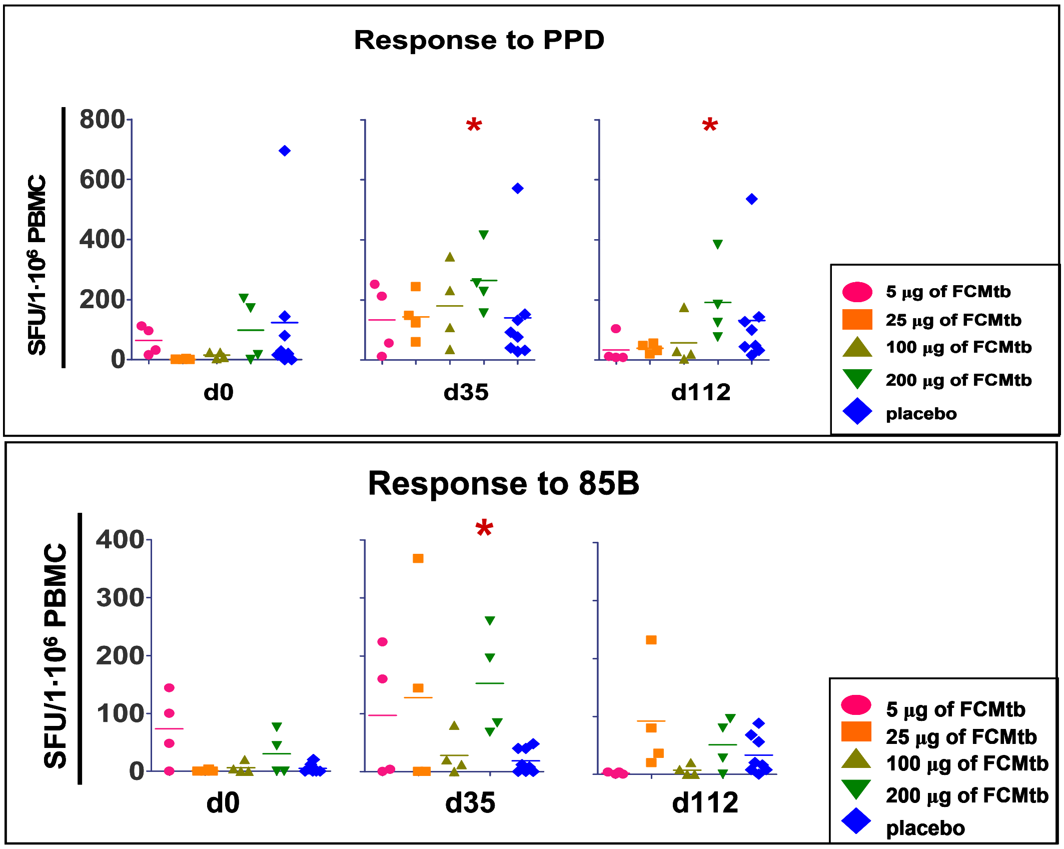

Figure 7 Cellular response (in terms of SFU/1•106 PBMC) against PPD and *M. tuberculosis* recombinant antigen 85B measured by ELISPOT. The red asterisks mean statistically significant differences with the placebo group.

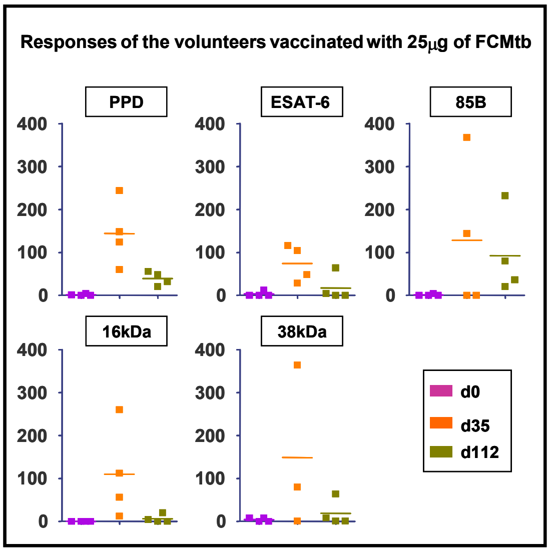

Figure 8 Cellular response (in terms of SFU/1•106 PBMC) against PPD and four *M. tuberculosis* recombinant antigens for the dose of 25µg of FCMtb.

1.3 Summary of the Known and Potential Risks and Benefits to Human Subjects

1.3.1 Potential Risks, Safety, Toxicity and Tolerance

Safety

Standard safety pharmacology tests were conducted with RUTI® by CIDASAL, a Clinical Research Organization belonging to the Harlan Group, and the results showed an appropriate safety margin. No potential adverse effects on general behaviour were found when assessed, except for slight central nervous system stimulating effects reflected in increased motor activity and sensitivity when using RUTI® at ≥300 µg/kg. No effect of the substance on cardiovascular parameters or respiratory system respiratory frequency was observed related to RUTI® when administered intravenously in anaesthetized Beagle dogs.

One *in vitro* study on the development of autoantibodies was also conducted in the Autoimmunity Area of LIRAD-Banc de Sang I Teixits of Germans Trias I Pujol Hospital, and the results concluded that RUTI® does not induce autoantibodies. Even that, antinuclear antibodies detection was included at screening and at the end of the Phase I Clinical Trial, to ensure that point, and no positive result was obtained.

Toxicity

Two single-dose and one repeated-dose toxicity studies were performed in Wistar Hannover rats by the CIDASAL under Good Laboratory Practice (GLP).

Two studies to evaluate local tolerance were conducted using mice and guinea pigs.

The two single-dose studies were performed in order to determine the maximum nonlethal and minimum lethal doses of the administration of RUTI® by subcutaneous and intravenous routes. Both studies were conducted using male and female rats, and a RUTI® concentration of 1.5 mg/kg. Rats were followed-up for 14 days, when they were sacrificed and necropsied. In none of the two studies abnormal clinical signs were found, no animals died and no relevant abnormalities attributable to RUTI® were seen, except for nodules in the injection site of 4/5 male and 1/5 female rats of the subcutaneous study. The conclusion is that 1.5 mg/kg of RUTI® is 1,000 fold higher than the intended dose in humans, therefore not necessary to administer higher doses to determine the subcutaneous or the intravenous maximum nonlethal and minimum lethal doses.

In the 4-week subcutaneous repeated-dose toxicity study, some clinical effects were recorded: slight anaemia and increased leukocyte count versus control group at 0.15 and 1.5 mg/kg; significant but slight decrease of total protein and albumin levels in sera at 1.5 mg/kg versus control group. Subcutaneous nodules and abscesses were observed at the injection site after 1.5 mg/kg of RUTI® (regardless of sex), as well as granulomatous reaction (especially but not only after 1.5 mg/kg). After this study, the non-observed adverse event level (NOAEL) was fixed as 0.015 mg/kg/day. This study also demonstrated the lack of toxicity of RUTI® (0.015, 0.15 and 1.5 mg/kg/day subcutaneous doses tested) on any reproductive organs, nor on female rats' oestrous cycle.

Two studies to evaluate local tolerance were conducted, in mice and guinea pigs. The results demonstrated that a well-tolerated subcutaneous node (of 0.5 – 1 cm of diameter) was detected in the injection site of all mice and all guinea pigs after each RUTI® administration. This nodule diminished along the time but its course was different in the two experimental animal models: while hadn't disappeared in mice by the end of the mice study, only one animal still had it when the guinea pig study finished.

Primary pharmacodynamic studies also gave useful and complementing information about toxicity and tolerance of RUTI® administration in mice, as well as in guinea-pig experimental models, a subcutaneous nodule in the injection site was observed after each inoculation.

The study with naturally infected Murciano-Granadina goats (4) in which no previous INH treatment was administered, the subcutaneous injection of RUTI® was well tolerated. Only a transient temperature increase (1 – 2 ºC) was recorded after each RUTI® inoculation that lasted 24 hours. At the injection site granulomatous indurations were observed. No deaths related to vaccination occurred.

The study with mini-pigs (4) demonstrated RUTI® to be very well tolerated: neither fever or weight decrease was recorded, nor were local adverse effects or mortality induced by the vaccination observed.

In conclusion, no relevant effects were observed after single or repeated dosing. Studies performed in mice, guinea pigs and goats showed a local transient inflammatory response at the injection site. No systemic toxicity (weight loss or increased granulomatous response) was detected in any of the experiments.

Phase I Clinical Trial Safety Aspects

The Phase I clinical trial was the first approach to evaluate the safety of the vaccine RUTI®. The safety of the vaccination to healthy volunteers was evaluated by physical examinations, AE reporting, inoculation point evaluations and safety and tolerability laboratory testing. The vaccine was safe in all doses tested.

As a global evaluation of its safety in healthy subjects, it was reported that the AEs of RUTI® vaccine were in line with other immunogenic products, the overall number being 175 in a study with a duration of 168 days for all 24 volunteers. Table 1~~Table 1~~, Table 2~~Table 2~~ and Table 3~~Table 3~~ summarize the results in terms of AEs reported. Headache was the AE most frequently reported.

No serious AEs were reported.

Only two episodes of clinical significance were found in physical examinations during the study:

- one occurred in a subject randomised to placebo
- the other (sialolithiasis) was addressed as not related to the investigational product.

The incidence of local AEs was superior with the highest doses of RUTI® (100 and 200 µg of FCMtb), the pain intensity being dose response and higher after the second inoculation (Figure 9~~Figure 9~~). In general all the local AEs were increasingly presented in the highest doses, and dose-related. The more important reaction seen was a granulomatous panniculitis in two volunteers, one of them vaccinated with 100 µg of FCMtb and the other with 200 µg of FCMtb. Both cases of panniculitis had negative microbiological cultures, proving to be sterile. All laboratory tests (haematology, clinical chemistry and urinalysis), vital signs (arterial pressure in supine position and heart rate [HR]) and 12-lead electrocardiograms (ECGs) did not show clinically relevant abnormalities.

Table 1 Systemic AEs. Number (%) of subjects reporting one or more treatment emergent AEs and number of AE occurrences by preferred term, possible or probable causality to the treatment and intensity. (O=Occurrences; S=Subjects).

Preferred term	Causality	Intensity	Placebo (n=8) O/S (%)	RUTI doses (in µg of FCMtb) 5 (n=4) O/S (%)	25 (n=4) O/S (%)	100 (n=4) O/S (%)	200 (n=4) O/S (%)
Feeling of body T[a] change	Possibly	Mild	0/0 (0%)	1/1 (25%)	0/0 (0%)	0/0 (0%)	0/0 (0%)
Vasovagal Syncope	Possibly	Mild	0/0 (0%)	0/0 (0%)	2/1 (25%)	0/0 (0%)	0/0 (0%)
Rhinorrea	Possibly	Mild	0/0 (0%)	0/0 (0%)	0/0 (0%)	1/1 (25%)	0/0 (0%)
Sneezing	Possibly	Mild	0/0 (0%)	0/0 (0%)	0/0 (0%)	1/1 (25%)	0/0 (0%)
Lymphadenopathy	Possibly	Mild	1/1 (12.5%)	0/0 (0%)	0/0 (0%)	0/0 (0%)	0/0 (0%)
Nasopharyngitis	Possibly	Mild	1/1 (12.5%)	0/0 (0%)	0/0 (0%)	0/0 (0%)	0/0 (0%)
Skin rash	Possibly	Mild	2/2 (25%)	0/0 (0%)	0/0 (0%)	0/0 (0%)	0/0 (0%)

Table 2 Local reactions and statistical differences between treatments groups

Local reaction	Placebo	5 µg FCMtb	25 µg FCMtb	100 µg FCMtb	200 µg FCMtb
**Erythema** (mild or moderate)	0	0	0	2†	3†
**Inflammation** (mild or moderate)	0	0	0	2†	5†
**Induration**	0	0	0	0	8‡
**Abscessification**	0	0	0	0	0
**Ulcer**	0	0	0	0	0
**Necrosis**	0	0	0	0	0

† Significant statistical differences ($p < 0.05$) compared to 5 µg, 25 µg and placebo groups
‡ Significant statistical differences ($p < 0.05$) compared to 5 µg, 25 µg, 100 µg and placebo groups

Table 3 Local AEs. Number (%) of subjects reporting one or more treatment-emergent AEs and number of AE occurrences by preferred term, possible or probable causality to the treatment and intensity. (O=Occurrences; S=Subjects)

				RUTI doses (in µg of FCMtb)			
Preferred term	Causality	Intensity	Placebo (n=8) O/S (%)	5 (n=4) O/S (%)	25 (n=4) O/S (%)	100 (n=4) O/S (%)	200 (n=4) O/S (%)
Injection site pain	Possibly	Mild	0/0 (0%)	0/0 (0%)	0/0 (0%)	1/1 (25%)	1/1 (25%)
Twitching	Possibly	Mild	7/4 (50%)	0/0 (0%)	1/1 (25%)	10/4 (100%)	4/3 (75%)
Panniculitis	Probably	Mild	0/0 (0%)	0/0 (0%)	0/0 (0%)	0/0 (0%)	1/1 (25%)
	Possibly	Moderate	0/0 (0%)	0/0 (0%)	0/0 (0%)	1/1 (25%)	0/0 (0%)

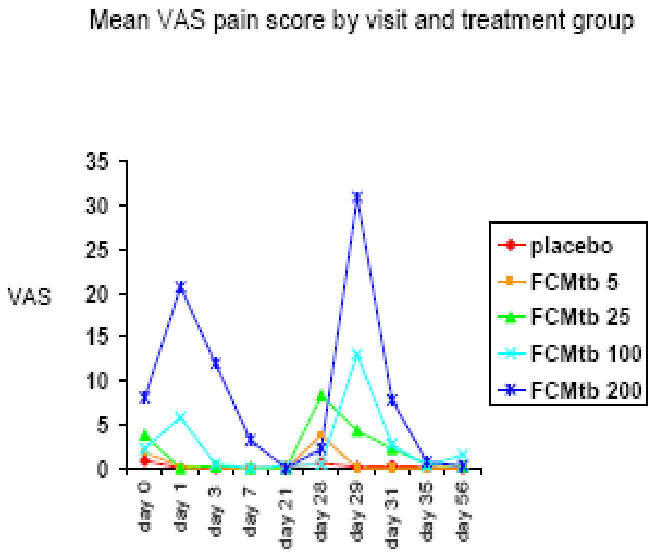

Figure 9 Mean Visual Analogue Scale (VAS) pain score by visit and treatment group

1.3.2 Potential Benefits

The main potential benefit of RUTI® vaccination is the ability to ensure the efficacy of LTBI short chemotherapy treatment, making the treatment compliance easier.

Nowadays, one third of mankind already has LTBI. After *M. tuberculosis* infection, bacilli persist in symptom-free individuals, causing disease in a 10% during the lifetime (a probability that increases to 10% every year in LTBI-Human immunodeficiency virus (HIV) co-infected

subjects). Although LTBI is a well-documented infection in medical practice, the approach to its treatment has largely been empirical, based on knowledge accumulated over many years. The current regimen implies six to nine -month INH treatment, what makes compliance difficult. Isoniazid is the cheapest drug for the treatment of *M. tuberculosis*, and it was demonstrated that a 9-month course was 90% effective (5), which is still considered the gold standard treatment. The lack of compliance has pushed the World Health Organisation (WHO) to promote the six months INH treatment, which is more easy to accomplish in those patients that really need this treatment as HIV infected subjects, whose reactivation towards TB disease incidence can be 10 to 100 times higher (6). Proposing a short treatment regimen consisting of one-month INH treatment plus two doses of RUTI® will make the LTBI treatment compliance easier by preserving the efficacy.

Additionally, RUTI® has demonstrated to have a prophylactic effect in mice and guinea pigs. In both cases, two inoculations were given to the animals three weeks apart, to be infected by a low dose aerosol after seven weeks. A BCG vaccinated group was also included in the experiment, to which a single shot with 10e6 colony forming units was given at the beginning of the experiment.

RUTI® demonstrated a similar prophylactic effect than BCG as observed when animals were sacrificed after three weeks post infection (Figure 10~~Figure 10~~). In the guinea pig experiment, RUTI® also increased the survival time of the animals, but not as much as BCG, as was expected because BCG is a live vaccine (Figure 11~~Figure 11~~).

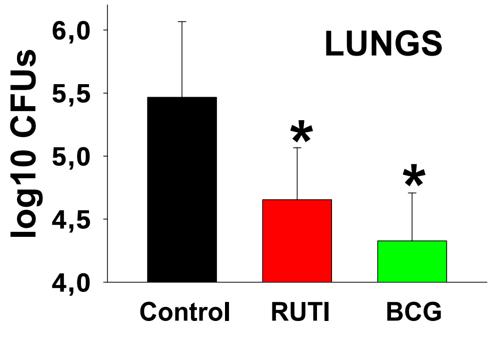

Figure 10 Prophylactic effect of RUTI® in mice, three weeks after a low dose aerosol infection. In both cases the difference with the control was significant (P<0.05). RUTI® B06 batch was used (n=12 in all groups)

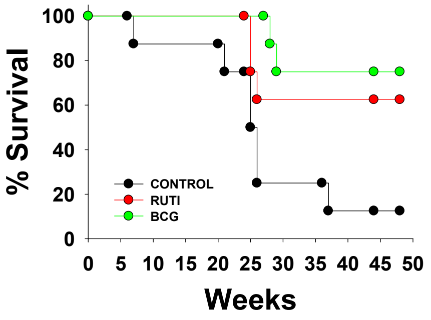

Figure 11 **Prophylactic effect of RUTI® in guinea pigs. Inoculation of RUTI® increased the survival of the animals but not at the same extent that in BCG. RUTI® B06 batch was used (n=8 in all groups)**

1.4 Description of and Justification for the Route of Administration, Dosage, Dosage Regimen and Treatment Period

As our group has shown in two published reviews, little animals (mice and guinea pigs) have such a tolerance in front of *M. tuberculosis* infection that need an important antigenic concentration to develop an immune response. That diminishes the importance of the dose-response experiments in experimental animal models, regarding to *M. tuberculosis*. Even that, different dose-response assays were performed, by measuring both the cellular and the humoral immune responses.

In non-infected animals, four dilutions of RUTI® vaccine were tested (1:1, 1:2, 1:4 and 1:8) and cellular immune response assessed. Determination of specific IFN-γ production by splenocytes after stimulation with PPD, BCG and the *M. tuberculosis* antigen Ag85B was done by ELISPOT method. The results showed a clear dose-immune response relationship was observed (Figure 12~~Figure 12~~).

In other assay, antibodies production in uninfected 129/Sv mice after three inoculations (on weeks 0, 2 and 4) of two RUTI® dilutions (1:1 and 1:5) was determined. The sacrifice revealed that none response was obtained with the animals immunised with the lower dose (Figure 13~~Figure 13~~).

Dose-Response was also analyzed by measuring the humoral immune response in *M. tuberculosis* infected mice (in means of antibodies production, (Figure 14~~Figure 14~~) and by analyzing the bacillary load in infected guinea pigs (Figure 15~~Figure 15~~).

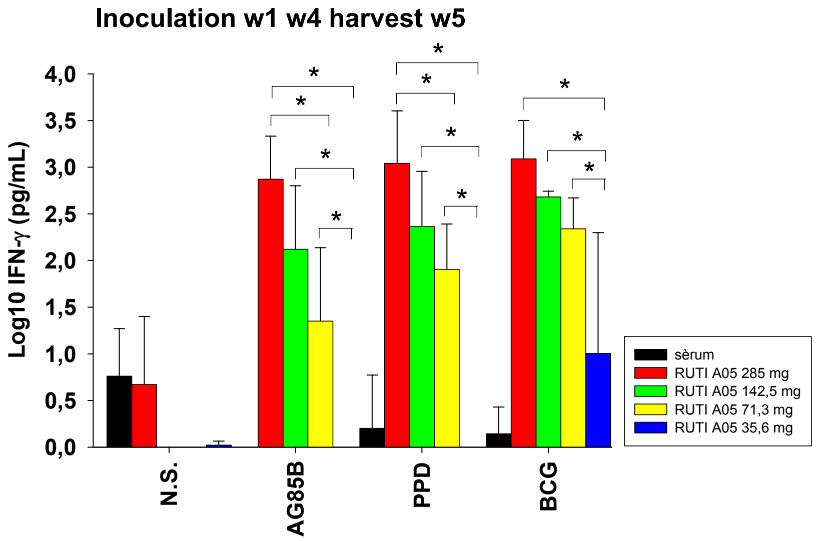

Figure 12 **A clear dose-cellular immune response relationship was observed when analyzing IFN-λ expression by splenocytes stimulated ex vivo by PPD, BCG and *M. tuberculosis* antigen Ag85B. RUTI® dilutions used to vaccinate the animals were: 1:1, 1:2, 1:4 and 1:8.**

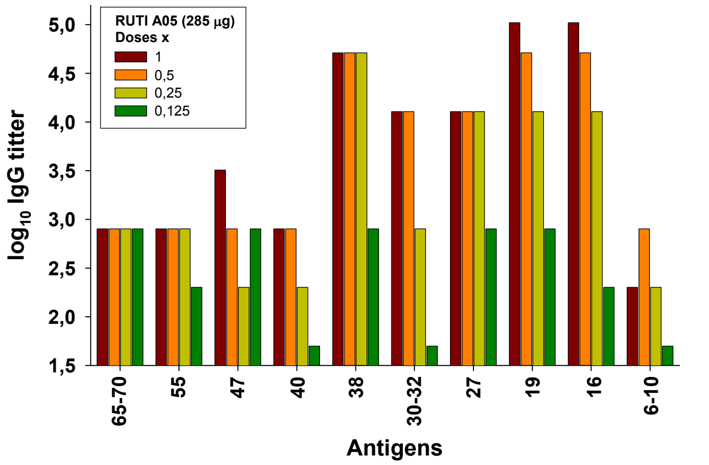

Figure 13 **Dose-response experiment to quantify from Western-Blot the response to different antigens**

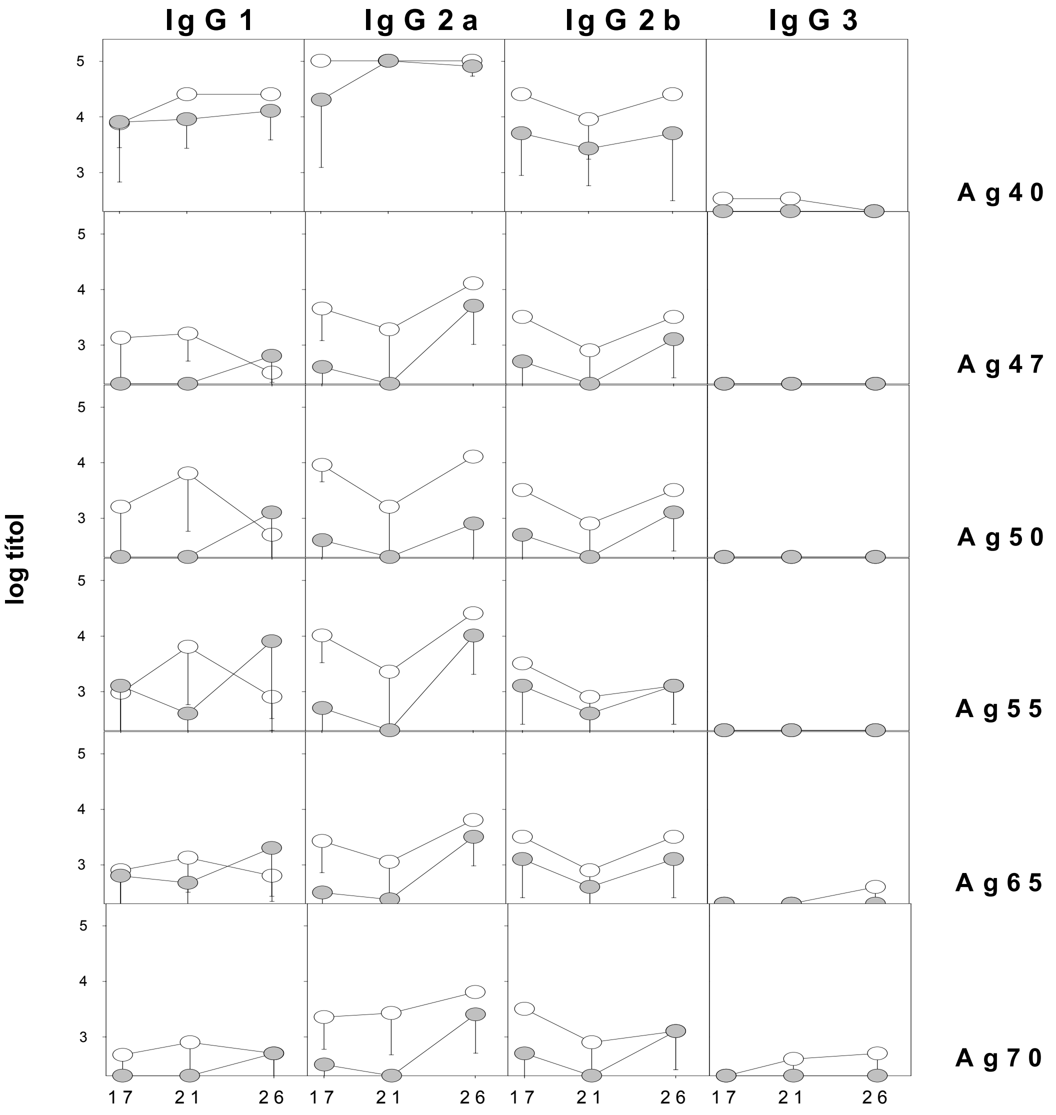

Figure 14 **Antibodies titres against 13 antigens in non-treated *M. tuberculosis* infected DBA/2 mice serum after three inoculations of RUTI® 1:1 (empty symbols) or 1:10 (grey symbols). Antibodies titres were tested to 13 antigens known to be present in RUTI® vaccine. Results showed that mice inoculated with RUTI® 1:10 dose secreted less antibodies.**

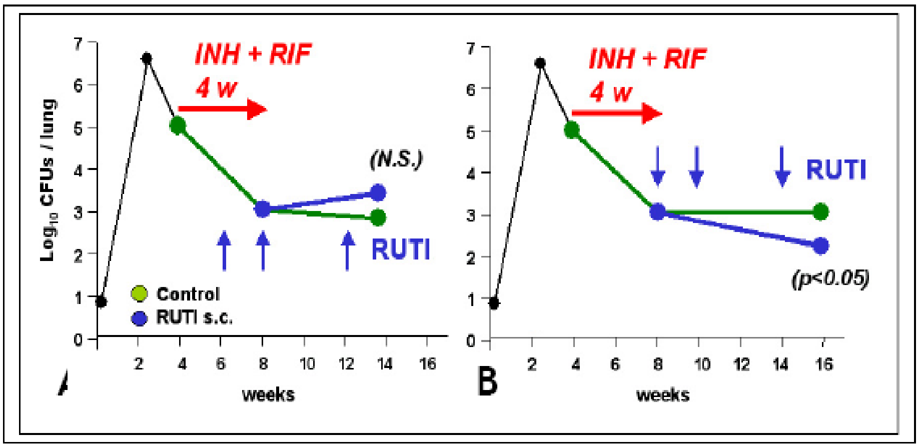

Figure 15 **Efficacy of three inoculations of RUTI® in guinea pig experimental model. In this experiment, two doses were tested: 24.5 µg of FCMtb, batch RUTI®-23 (A), or 262 µg of FCMtb, batch RUTI®-A04. None protection was observed when using the lower dose, but obtained with the approximately 10-fold higher one (B)**

Finally, a study evaluating the mechanism of action showed that two inoculations were enough, demonstrating its efficacy. That was suggested by previously assays performed in uninfected animals, where the antibodies production was determined after two or three inoculations of RUTI® that showed no differences at all.

Studies performed in uninfected animals administering them one, two or three inoculations of RUTI® (185 µg of FCMtb, batch RUTI®-14B), showed an erratic and lower levels of protections with a single inoculation, in means of none immune response detected against some of the *M. tuberculosis* antigens; but no difference were found in means of antibodies levels when comparing the two and three inoculations. (Figure 16~~Figure 16~~).

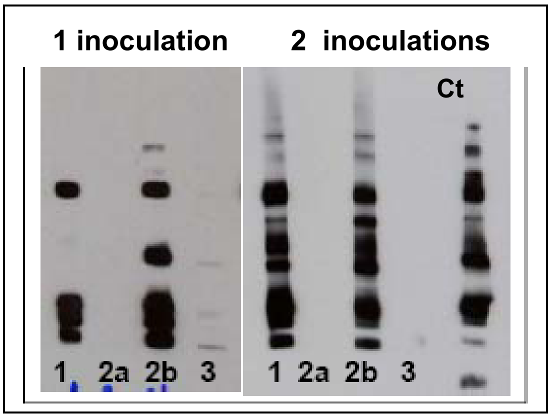

Figure 16 **Western Blot image representing an assay where uninfected mice were immunised with 1 or 2 inoculations of RUTI®-14B (185 µg of FCMtb)**

The rational for the dose escalation at Phase I clinical trial was based on several premises. In the experimental animal models of efficacy, the optimal dose was fixed to the inoculation of a total amount of 200 μg of FCMtb. On the other hand, TB experimental models also reveal that the reaction against PPD to perform the tuberculin skin test requires the inoculation of 0.4 μg in guinea pigs, in order to have the optimal reactivity. In humans the required amount is 0.04 μg. That's why was supposed that the inoculation of a concentration of FCMtb 10 times less that the optimal in guinea pigs, would also have efficacy in humans, and so the optimal dose was fixed to 25 μg.

The vaccine was administered twice to the volunteers (28 days apart), as the animal experiments suggested (one administration was not enough, but no differences had been encountered in the immunological response when comparing two to three inoculations). The results revealed an increased cellular immune response (in means of IFN-γ) production against PPD, BCG and *M. tuberculosis* antigens after the second inoculation vs. the first. Also in immunogenicity terms, although it seemed to exist a dose-related increase on the immune response for some stimuli (PPD and BCG), the polyantigenic response seemed to be better at lower doses (5 and 25 μg). That's why these are the doses we are going to use in the Phase II clinical trial. We will also include the 50 μg to evaluate its safety, tolerability, and ability to induce a polyantigenic immune response.

1.5 Description of the Population to be Studied

The present trial has been designed to test the safety, tolerability and immunogenicity of a treatment regimen against LTBI consisting of RUTI® vaccination after pre-treatment with INH for one month. HIV infected and non-infected subjects will be enrolled as subjects. As a Phase II trial, all the subjects will be TB latently infected (with a positive tuberculin skin test (TST)+ and a QuantiFeron-TB-Gold positive result). Recruitment will be done prospectively in 96 LTBI HIV negative (n=48) and positive (n=48) subjects, once lack of active TB would be ensured. Including HIV- subjects in this trial will provide essential comparative data about safety and immunogenicity that will allow a better understanding of the HIV+ patients demonstrated responses to the vaccine.

There will be no gender preferences for subjects enrolled into this clinical trial.

2. TRIAL OBJECTIVES AND PURPOSE

The aim of the trial is to assess the safety, tolerability and immunogenicity of two doses of RUTI® vaccine administered four weeks apart after one month pre-treatment with INH.

The trial will be double-blinded, randomized and placebo-controlled with 96 subjects (48 HIV- and 48 HIV+ subjects).

OBJECTIVES:

- To evaluate the safety and tolerability of three different doses (5, 25 and 50 µg of FCMtb) of one formulation of the novel antituberculous vaccine RUTI® in subjects with LTBI compared to placebo.
- To evaluate the immunogenicity of three different doses (5, 25 and 50 µg of FCMtb) of one formulation of the novel antituberculous vaccine RUTI® in subjects with LTBI compared to placebo.

3. TRIAL DESIGN

3.1 Endpoints to be Measured During the Trial

Primary endpoint: Safety and tolerability

- Adverse events
- Vital signs, including blood pressure, pulse, respiratory rate, body temperature
- Physical examinations
- Laboratory tests (standard haematology, serum biochemistry and urinalysis; and CD4 counts and viral load in HIV+ patients)

Secondary endpoint: Cellular immunogenicity response

- Immunogenicity by measuring the number of IFN-γ spot-forming units among PBMCs stimulated with five antigens using a non commercial ELISPOT test, after cryopreservation of PBMC from peripheral blood.
- Immunogenicity by measuring the number of IFN-γ spot-forming units among PBMCs stimulated with two (ESAT-6 CFP-10) antigens using a commercial ELISPOT test: TIGRA, after cryopreservation of PBMC from peripheral blood.
- Long-term whole blood assay (stimulation of diluted whole blood with PPD for seven days), the "*WHO assay*".
- Sera will be frozen to be further tested for humoral response against *M. tuberculosis* antigens (antibodies to LAM, 16kDa and 38kDa mycobacterial antigens).

Additional laboratory markers to be determined:

- NAT-2 genotyping: to account for INH metabolism status.
- Retention of cryo-preserved PBMC for retrospective testing.

3.2 Description of the Type/Design of Trial to be Conducted (Diagram of Trial Design, Procedures and Stages)

The trial will be a double-blinded, randomized and placebo-controlled to test three doses of RUTI® and will last three months.

Three different RUTI® doses and placebo will be tested, randomizing assigned both in HIV+ and HIV- subjects, as figured in Table 4~~Table 4~~. Each subject will be randomized to receive one of the four treatments (placebo, 5, 25, 50 µg), after completion of one month INH pre-treatment (one tablet of 300mg/day, p.o.). Each subject will receive two administrations of the same treatment, 28 days apart. Subjects will be monitored until one month after the second inoculation with RUTI®. All trial procedures will be performed according to the schedule displayed in Figure 17~~Figure 17~~.

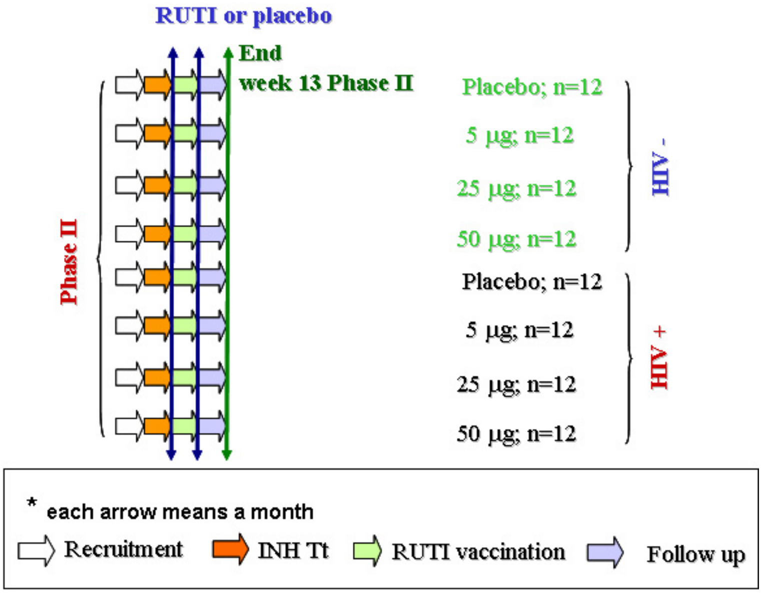

Figure 17 Chart summarizing the trial design of both phases of the clinical trial. Subjects will be recruited at the three PAREXEL South Africa sites.

Every subject will receive the Informed Consent, with all the activities and requirements of the trial. After signing it, they will be screened in order to guarantee the compliance of the inclusion and exclusion criteria.

Once included in the trial, the subjects can do their daily activities, always following the principal investigators instructions, and communicating any event that could interfere with the trial's objectives.

The day-to-day management of the clinical trial has been organized in function of the laboratory work and the clinical part to be done. An organisation chart including the decision-making mechanism for safety and immunogenicity is included below, the timing has also been added (Figure 18~~Figure 18~~).

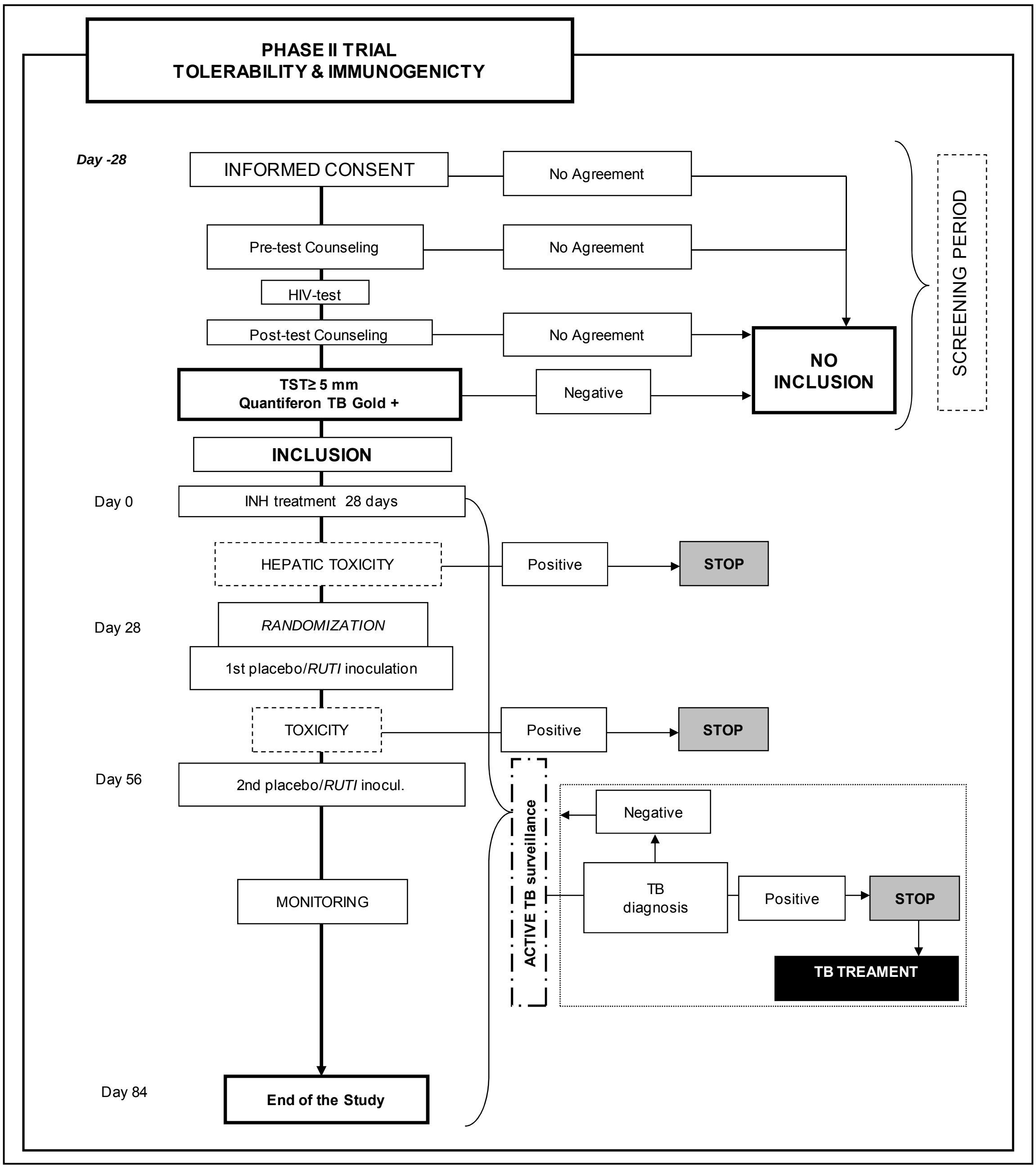

Figure 18 Day to day management

3.3 Randomization and Blinding

After all subjects received one-month of INH treatment, a total of 48 HIV+ subjects and 48 HIV- subjects will be randomised to one of four pre-treatment groups (12 subjects in each treatment group) and will receive either placebo, 5, 25 or 50 µg of FCMtb RUTI® as indicated in Table

4~~Table 4~~ below. Each subject will have two inoculations of the applicable RUTI® dose or placebo, 28 days apart, once the safety is ensured.

Table 4 Groups involved in the 2 phases of the clinical trial

	n	LTBI/HIV-	n	LTBI/HIV+
**Phase II**	12	RUTI® (5µg of FCMTb)	12	RUTI® (5µg of FCMTb)
	12	RUTI® (25µg of FCMTb)	12	RUTI® (25µg of FCMTb)
	12	RUTI® (50µg of FCMTb)	12	RUTI® (50µg of FCMTb)
	12	placebo	12	placebo

The medication (RUTI® vials) will be prepared giving the vero or placebo after the random attribution to a serial number. The randomization list will be sent to the persons responsible of the medication storage and labelling and the rest of the personnel will be kept blinded.

Medication will be transferred to pharmacists at each trial site with the correspondent sealed envelope labelled with the serial number containing information on the nature of the medication inside. This envelope will be opened only in case of necessity (i.e. toxicity), and the reason/s, the responsible/s, time and signature/s will be included in a form.

The investigators will follow the trial's randomization procedures, and will ensure that the code will only be broken in accordance with the protocol. In case of any premature unblinding of the investigational product (e.g. accidental unblinding, unblinding due to a serious adverse event (SAE)) they will promptly document and explain it to the sponsor.

Stratification will be required for this trial.

If one subject withdraws from the clinical trial before receiving the first RUTI® or Placebo inoculation, he/she will be replaced by another subject to complete the trial intended numbers, except if the trial has to be ended because of safety issues or the total number of subjects having received at least one inoculation is reasonably sufficient to achieve the study objectives. This new subject will receive the corresponding medication that the one replaced would have received and will be assigned to the corresponding identification subject code.

3.4 Description of the Trial Treatments and the Dosage and Dosage Regimen of the Investigational Product. Dosage Form, Packaging, and Labeling of the Investigational Product

The trial drug is RUTI® vaccine, and the drug substance is a detoxified, pasteurized mixture of different protein antigens and lipids from fragmented cells of *M. tuberculosis* strain 511 FCMtb.

RUTI vaccine is presented as a dry powder for reconstitution containing 66.7 µg of FCMtb per vial in a liposomal lyophilised formulation.

The qualitative and quantitative composition per vial of RUTI® vaccine is shown next table (Table 5~~Table 5~~):

Table 5 The qualitative and quantitative composition per vial of RUTI® vaccine

COMPONENTS	UNIT PER VIAL	FUNCTION
**DRUG SUBSTANCE**		
FCMtb	66.7 µg	Immunogen
**EXCIPIENTS**		
Sucrose	20000.0 µg	Charge substance (freeze-drying) and cryoprotector
Soy lecithin [1]	845.8 µg	Liposome forming agent
Sodium cholate	92.0 µg	Tensoactive
Sodium chloride [2]	20.8 µg	Solvent

1 Containing Phosphatidylcholine (NLT 94.0%)
2 Added as NaCl 0.9% solution.

RUTI® vaccine is formulated to be administered by subcutaneous route after reconstitution with water for injection (0.4 mL) to give a suspension containing 166.7µg /mLof FCMtb. Next table (Table 6~~Table 6~~) shows the concentration of each component per vial after reconstitution.

Table 6 The concentration of each component per vial after reconstitution

Components	Concentration per vial
**DRUG SUBSTANCE**	
FCMtb	166.7 µg/mL
**EXCIPIENTS**	
Sucrose	50,000.0 µg/mL
Soy lecithin	2,114.4 µg /mL
Sodium cholate	230.0 µg /mL
Sodium chloride	52.1 µg /mL

RUTI® vaccine is manufactured by ARCHIVEL FARMA, S.L. (Badalona. Catalonia, Spain) under GMP.

The manufacturing process of RUTI® has been published elsewhere (7). Briefly, *M. tuberculosis* are cultured for three weeks in Middlebrook 7H11 agar at 37ºC, under a progressive conditions

of low pH and pO_2. Colonies are carefully removed and mechanically disrupted using silica-zirconium beads and a PBS buffer with 4% Triton-X114. After centrifuging at 845 g to remove the entire cells, the pellet is centrifuged at 20,000 g, the lipidic supernatant is discarded and the final product is washed, pasteurized at 65ºC for 60 minutes, and lyophilized.

The freeze-dried drug substance will be finally formulated as liposome drug product. To do this liposome preparation is undertaken after mixing at high speed (lipid phase of the liposomes consisting of soy lecithin solution, sodium cholate solution, and the drug substance FCMtb; the aqueous phase consisting of saline solution (NaCl) diluted with bidistilled apyrogenic water). As charge substance, 5% sucrose is used.

Dosage and Administration

A total of three doses of RUTI® vaccine will be tested: 5, 25 and 50µg of FCMtb, chosen from the Phase I clinical trial experience in terms of tolerability and polyantigenic immune response.

A single dose of the vaccine or placebo (0.3 mL) will be administered twice to the subjects, 28 days apart (on days 28 and 56), by subcutaneous route, after one-month of INH pre-treatment. Sites of injection will be alternated (i.e., the injection should be given subcutaneously in the deltoid muscle area [right or left] that is opposite to the previous injection site). Both the injection site and the opposite deltoid muscle should be evaluated during the course of the trial. The exact date and times of each trial drug administration will be recorded in the CRF together with the side of the injection (right or left deltoid muscle).

Subjects will be followed-up for safety and immunogenicity parameters until the end of the trial

Packaging and Labelling

The trial drug will be packaged in vials containing the active substance and excipients as indicated in Table 5~~Table 5~~. Placebo will be packaged in the same type of vials containing identical amount of excipients without active substance. The vials will be labelled RUTI® or placebo, respectively. Before administration, the pharmacist of the pharmaceutical departments at each site, as designee of the principal investigator, will reconstitute and dilute the content of the vials according to the dose assigned to each subject. Therefore, the pharmacist will not be blinded. The pharmacist will label the administration syringe with the subject's randomization number. For these processes, the pharmaceutical departments at each site adhere to specific rules as described in the Good Manufacturing Practice (GMP) Annex 13 requirements.

Preparation and Handling

The trial drug will be packaged in vials containing the active substance and excipients as indicated in Table 5~~Table 5~~. Placebo will be packaged in the same type of vials containing identical amount of excipients without active substance. The vials will be labelled RUTI® or placebo, respectively. Before administration, the pharmacist of the pharmaceutical departments at each site, as designee of the principal investigator, will reconstitute and dilute the content of the vials according to the regimen (trial drug or placebo) and dose assigned to each subject. Therefore, the pharmacist will not be blinded. The pharmacist will label the administration syringe with the subjects' randomization number. For these processes, the pharmaceutical departments at each site adhere to specific rules as described in the Good Manufacturing Practice (GMP) Annex 13 requirements.

Preparation and Handling

The trial drug (and placebo) will be stored between 2° - 8°C in the pharmaceutical departments at each site until reconstitution proceeds.

Allow the trial drug and placebo to equilibrate at room temperature for at least 5-10 minutes before reconstituting it with sterile water for injection. Mix thoroughly and gently to minimize frothing in order to ensure complete resuspension. After convenient dilution accordingly with the Working Document, a total volume of 0.3 mL will be administered subcutaneously.

3.5 Expected Duration of Subject Participation

Each subject will be screened within the 28 days period prior to Visit 1 (Day 0). Should the screening period be longer than 28 days subjects may be re-screened after agreement between the sponsor and the investigator. After the inclusion in the trial (d0), the subjects will remain in the trial for 84 days. See Figure 18~~Figure 18~~.

3.6 Stopping Rules or Discontinuation Criteria for Individual Subjects, Parts of Trial and Entire Trial

An independent monitoring committee, the Data Safety Monitoring Committee (DSMC), will be established to monitor the trial subjects, and to assess the progress of the clinical trial and the safety data. The DSMC will meet at predetermined intervals to review the unblinded results.

As its main objective is the safety of the patient, the DSMC will make important trial-related decisions. They will decide whether to withdraw a subject for safety reasons and will recommend to the sponsor whether to continue, modify or stop the clinical trial.

Every decision of the DSMC will be taken after consensus of all the members of the committee.

3.7 Drug Accountability Procedures

The clinical investigator or designee is responsible for ensuring that all trial drug received at Pharmaceutical Services is inventoried and accounted for throughout the trial. The dispensing of trial drug to the subject will be documented on the drug accountability form. Unused or partially used trial drug will be stored and disposed of according to the sponsor's instructions. Contents of the trial drug containers must not be combined.

Trial drug must be handled strictly in accordance with the protocol and the container label and will be stored in a limited access area under appropriate environmental conditions. Unused trial drug must not be disposed of until the sponsor's monitor has completed drug accountability during an on-site monitoring visit. The return to the sponsor or destruction on-site of used and unused trial drug will be documented on the Drug return form.

Trial drug will be dispensed under the supervision of the investigator, or a qualified member of the investigational staff. Trial drug will be supplied only to subjects participating in the trial. Trial drug will not be re-labelled or reassigned for use by other subjects. The investigators agree to dispense the trial drug only at the Clinical Units agreed upon with the sponsor.

3.8 Randomization and Maintenance of Trial Treatment Randomization Codes and Procedures for Breaking Codes

A randomization schedule will be provided by Biostatistics from Clinical Data Services of PAREXEL International (South Africa), Bloemfontein. The randomization schedule will be generated using RANDPLAN, a locally developed SAS® macro utilizing PROC PLAN procedure in SAS® (8).

The investigators, ARCHIVEL FARMA, S.L, and any personnel involved in subjects' assessment, monitoring, analysis and data management will be blinded to the subject assignment. Treatments will be packaged by the pharmaceutical departments at each site and/or designee and supplied to the investigator as identical-appearing treatments.

Two copies of emergency code break envelopes will be prepared, one copy for the ARCHIVEL FARMA, S.L. and one copy to be kept at the pharmacy. The envelopes should be maintained in a secure locked place throughout the trial while still allowing access for emergency code breaking. In case of a medical emergency, when the trial drug assignment is needed to make treatment decisions for the subject, the investigator may unblind the subject's drug assignment. ARCHIVEL FARMA, S.L. and the medical monitor should be notified of the event prior to breaking the code, if possible. If this is not possible, ARCHIVEL FARMA, S.L. should be notified immediately afterwards, and the subject's drug code assignment should not be revealed. The circumstances leading to the breaking of the code should be fully documented, in the investigator’s trial files and in the subject’s source documentation. In studies conducted in the EU, for AEs that are defined as: Suspected Unexpected Serious Adverse Reaction (SUSAR), ARCHIVEL FARMA, S.L or designee may break subject’s code for possible regulatory submission.

All randomization code break envelopes will be collected and returned to ARCHIVEL FARMA, S.L. or designee at the completion of the trial.

4. SELECTION AND WITHDRAWAL OF SUBJECTS

4.1 Inclusion Criteria

Subjects who meet the following criteria will be considered eligible to participate in the clinical trial:

1. Asymptomatic adult aged 18 up to 50 years.
2. No evidence of active TB (Section 8).
3. No clinically significant finding at the discretion of the investigator.
4. Willingness to undergo an HIV test.
5. Resident in or near trial site for the duration of the trial.
6. Willingness to allow the investigators to discuss the patient's medical history with his usual doctor or HIV physician.
7. No donation of blood for 56 days prior to screening and agreement to refrain from blood donation during the trial.
8. Willing and able to provide written informed consent.
9. Positive tuberculin skin test (TST +), (≥5 mm induration) and Quantiferon TB Gold positive result (according to manufacturers instructions).
10. Reliable contraception to be used by female subjects during the clinical trial.
11. Additional inclusion criteria for HIV+ groups:

- HIV antibody positive.
- CD4 count ≥350 cells/mL on a single CD4 count at the period of screening.
- Subjects on anti-retroviral treatment can be included if clinically stable.

4.2 Exclusion Criteria

Subjects who meet one or more of the following criteria will not be considered eligible to participate in the clinical trial:

1. Any deviation from the normal range in biochemistry or haematology blood tests or in urine analysis that is considered to be clinically significant at the discretion of the

investigator. Values of Hb, WCC, platelet count, AST/ALT and creatinine should be in a normal range accordingly to the normal laboratory values.

2. Use of any investigational or non-registered drug, vaccine, or medical device other than the trial vaccine within 30 days prior to dosing of trial vaccine, or planned use during the trial period.
3. Administration of chronic (defined as more than 14 days) immunosuppressive drugs within six months of vaccination and required throughout the duration of the trial (for corticosteroids this means prednisolone or equivalent at ≥ 0.5 mg/kg/day).
4. Female of child bearing potential who intends to become pregnant during the trial.
5. Females who are pregnant, lactating, or of child bearing potential with a blood HCG positive result 24-48 hours at the screening period, or prior to every injection of RUTI®.
6. Any AIDS defining illness according to the CDC classification system for HIV infection (Appendix 17.1).
7. Presence of active (previously undiagnosed) TB or being on TB treatment.
8. Suspected or known current alcohol abuse (alcohol intake questionnaire, Appendix 17.2).
9. Suspected or known substance abuse.
10. Presence of any underlying disease, specifically autoimmune disease, asthma, angioedema, bleeding disorders, uncontrolled hypertension and diabetes, and any other disease that compromises the diagnosis and evaluation of response to the vaccine, excluding HIV.
11. Administration of immunoglobulins and/or any blood products within three months prior to the planned administration of the vaccine.
12. Any history of anaphylaxis in reaction to vaccination and/or other medication.
13. Investigator assessment of lack of understanding or willingness to participate and comply with all requirements of the trial protocol.
14. Any other finding which in the opinion of the investigator would significantly increase the risk of having an adverse outcome from participating in the trial.
15. Exclusion criteria relating to INH pre-treatment

- Weight less than 40 kg.

- Known or suspected hypersensitivity to INH.
- Self reported chronic liver disease or symptoms suggesting active hepatitis (jaundice, nausea, vomiting, right upper quadrant pain, dark urine, pale stools).
- Alcohol use exceeding 28 units per week (men) or 21 units per week (women) (see alcohol intake questionnaire, Appendix 17.2).
- History of convulsions.
- History of psychosis.
- Peripheral neuropathy grade 2 or greater.
- Three months post-partum.
- Concomitant medication with phenytoin, carbamazepine; warfarin; theophylline; disulfiram; selective serotonin re-uptake inhibitor antidepressants (e.g. citalopram, fluoxetine, paroxetine, sertraline); oral ketoconazole or itraconazole.

16. Additional exclusion criterion for HIV negative groups:
    - Any confirmed or suspected immunosuppressive or immunodeficient condition, including asplenia.
17. Additional exclusion criterion for HIV+ groups
    - CD4 count < 350 cells/mL.

4.3 Subject Withdrawal Criteria

Every subject included in the trial can withdraw from it at any moment, contacting immediately the investigators to inform them about it. Although the subject is not obligated to give his/her reasons for withdrawing prematurely from a trial, the investigators will make a reasonable effort to ascertain the reason(s) and to record them in the CRF, while fully respecting the subject's rights. This subject will be replaced for another subject excepting if the trial has to be ended because safety issues, if the subject has received at least the first RUTI® or Placebo inoculation or if the total number of subjects having received at least one inoculation is reasonably sufficient to achieve the study objectives. This subject will receive the corresponding medication that the subject replaced would have received.

The investigators can withdraw a subject from the clinical trial for one of the reasons below:

- Lost to follow-up

- Withdrawal of consent
- Subject is not in compliance with requirements of the trial, including inclusion, exclusion criteria, and prohibitions and restrictions
- The trial is prematurely stopped or halted (e.g. clinical halt)
- Discontinuation of trial treatment (final assessments will be obtained). A subject will be discontinued from trial treatment if the investigator believes that for safety reasons (e.g. AE) it is in the best interest of the subject to stop treatment.

If a subject is withdrawn because an AE, he/she will be followed-up by the Investigators until resolution or until reaching a clinically stable endpoint.

If the investigator decides to terminate or to suspend the trial without prior agreement of the sponsor, the investigator will promptly inform the trial subjects, assuring them appropriate therapy and follow-up, and will inform the sponsor and the regulatory authority, providing a detailed written explanation of the termination or suspension.

5. TREATMENT OF SUBJECTS

5.1 Treatment to be Administered (Product, Dose, Dosing Schedule, Route of Administration, Treatment Period, Follow-up Period for Subjects

Four weeks of INH pre-treatment will be administered to all subjects (subjects will keep a diary card to ensure INH treatment compliance), prior to randomization to receive either placebo or RUTI® s.c. twice, on 28th and 56th day (28 days apart). The criterion to be met prior to randomisation is to complete one month of INH (assessed by self reported adherence to INH) and no evidence of active TB prior to vaccination.

The monitoring will include complete physical examinations, laboratory sampling, blood extractions to perform safety and immunogenic tests, HIV+ status evaluation (CD4 counts) when required, and INH pre-treatment surveillance by self-reported adherence to INH. All the patients will be tightly followed-up, and an active TB surveillance will be done (see Section 8). A subject will be considered to have completed the trial if he or she completed all required assessments at the end of the follow-up period.

All the planned activities of the trial are shown in the figure below (Figure 19~~Figure 19~~). As indicated in this figure, the TST, Quantiferon Gold and HIV tests, and chest X-ray will be done at a pre-screen visit. The HIV test will be repeated at screening if the results were negative at the pre-screen visit. In the event that the pre-screen tests were performed longer than 4 months before the screening visit, it may be required to repeat the tests at screening. The chest X-ray may however be repeated at screening at the discretion of the investigator. As part of the monitoring plan there have been scheduled:

- the activities designed to screen the population for the trial
- the Safety measures
- Immunogenicity measures
- Other important activities for the trial are marked: Vaccine/Placebo Inoculation and decision to do it; INH pre-treatment and participant reports to verify its compliance; and active TB surveillance.

All the relevant activities of the subjects will be reported on Case Report Forms (CRF).

Phase II: Safety and Immunogenicity of INH 1 month plus RUTI																
	Study design															
		INH Tt			Pre 1st dose	1st dose	Post 1st dose				Pre 2nd dose	2nd dose	Post 2nd dose			
	**HIV+ & HIV-subjects**															
Visit	*S*	1	2	3	4		5	6	7	8	9		10	11	12	13
Day	.-28/-1	0	7	21	28		29	31	35	49	56		57	59	63	84
Week	.-4/-1	1	2	4	5				6	8	9				10	13
Information to the patient, Informed consent	X															
Clinical History	X															
Inclusion/exclusión Criteria	X	X														
Vaccine inoculation Decision				X						X						
Vital Constants	X	X	X	X	X		X	X	X	X	X		X	X	X	X
Physical examination	X	X	X	X	X		X	X	X	X	X		X	X	X	X
Pregnancy test	X				X						X					X
Serology (HIV, VHB, VHC)[1]	X															
TST + Quantiferon[1]	X															
Chest X-Ray[1]	X															
Chest CT					X											X
ECG	X									X						X
Concomitant medication	X	X	X	X	X		X	X	X	X	X		X	X	X	X
General Laboratory sampling	X			X	X				X	X	X				X	X
Immunogenicity sampling		X			X				X		X				X	
CD4 counts (in HIV+)	X				X					X					X	X
Viral load (in HIV+)	X				X					X					X	X
Inoculation of RUTI or placebo						X						X				
INH provision		X	X	X												
INH pre-treatment 300 mg/daily																
Local inoculation valoration						X	X	X	X	X	X	X	X	X	X	X
Adverse events																
Spontaneous																
After questioning		X	X	X	X	X	X	X	X	X	X	X	X	X	X	X
Active TB surveilance	X															

Figure 19 Programmed activities included in the monitoring of all the subjects included in the trial: screening activities, safety measures; Immunogenicity measurements and other important activities for the trial

[1] ***The HIV test, TST, Quantiferon Gold test and chest X-ray will be performed during a pre-screen visit. The HIV test will be repeated at screening if the results were negative at the pre-screen visit. In the event that the pre-screen tests were performed longer than 4 months before the screening visit, it may be required to repeat the tests at screening. The chest X-ray may however be repeated at screening at the discretion of the investigator.***

5.2 Prohibitions and Restrictions. Medication(s)/Treatment(s) Permitted and not Permitted Before and/or During the Trial

Potential subjects must be willing to adhere to the following prohibitions and restrictions during the course of the trial to be eligible for participation.

1. Must remain at the trial centre from at least 30 minutes after the vaccine administration.
2. Subjects will be advised not to donate blood during the trial and for at least two weeks after completion of the trial or to participate in an investigational drug trial for at least one month completion of the trial.
3. Must refrain from strenuous exercise of all types during the trial.
4. Subjects are strongly encouraged not to use alcohol and illicit substances during the entire trial.
5. Subjects are strongly encouraged not use medications or vaccines (excluding anti-retro viral treatment or users of anti-depressant drugs on stable dose) less than two weeks prior to baseline and during the trial.

If any concomitant medication is needed, it must be reported to the principal investigators and accepted by them. Principal investigators will be also responsible for treatment needed for AEs. Any treatment taken by subjects will be recorded in the CRF, as its dose and duration.

5.3 Procedures for Monitoring Subject Compliance

Trial drugs (RUTI® and placebo) will be administered in the controlled environment of a clinical research centre, under direct observation of the administration of the trial drug by the trial staff to ensure compliance with trial requirements. The date and time of each trial drug administration will be recorded in the CRF. Subjects must take their INH pre-treatment supervised by a peer nominated treatment supporter at home, workplace or in the community or alternatively take self supervised pre-treatment, which is the standard for INH preventive therapy.

6. ASSESSMENT OF IMMUNOGENICITY

6.1 Immunogenicity Parameters

Samples to perform immunogenicity testing will be collected at specified visits sampled according to the events schedule activities (Figure 19Figure 19). Cellular mediated immunity will be assessed using ELISPOT and ELISA techniques. IFN γ Spot Forming Units will be measured in the trial subjects' peripheral blood after stimulation with up to five stimuli (ESAT 6, 16 kDa, Ag85B and 38 kDa *M. tuberculosis* antigens, and PPD) for 18 hours. A long-term assay interferon (WHO assay) will be performed by stimulating the whole blood for seven days with PPD. One commercial TIGRA (TSPOT TB assay) will also be performed. Peripheral blood mononuclear cells will be frozen for future immune assays if required. Serum will also be frozen to be further tested for the antibody-mediated immunity against *M. tuberculosis* antigens.

6.2 Methods and Timing for Assessing, Recording and Analysing of Immunogenicity Parameters

A total of 3 mL of whole blood (in three QTF-Gold In Tube tubes: Nil, TBA and Mitogen tubes, 1 mL per tube) of each subject will be sampled for the QuantiFeron assay required during pre-screening.

A total of 306.5 mL of blood of each subject will be sampled at each time point for the immunogenicity testing (according to the event scheduled activities), in different collection tubes:

- one plain tube for obtaining serum (8.5 mL)
- onetwo sodium heparine tubes (6 mL each)
- two Cell Preparation Tube ™ (CPT) Vacutainer tubes (8 mL)

All the collection data will be recorded on the CRFs.

The samples will be properly labelled and sent to the laboratory as soon as possible (preferably within 30 minutes after samples have been collected). The PBMC processing will be done at the most, 4 hours after bleeding.

After the samples' testing for immunogenicity, the results will be evaluated by comparing the titres obtained on the different days of the follow-up, those for each group. The comparison between treatments will also be done.

7. ASSESSMENT OF SAFETY

7.1 Safety Parameters: Methods and Timing for Assessing, Recording and Analysing

During the trial, approximately 330 mLnot more than 200 mL of blood (excluding repeat and re-screen samplesexcluding repeat samples) for each subject will be sampled at the time points indicated in the programmed activities; includes safety laboratory samples, immunogenicity testing and plasma for assessing the HIV viral load and CD4 levels (Figure 19Figure 19).

The samples will be properly labelled and sent to the central laboratories as soon as possible. The investigators will review the laboratory report, document this review, and record any clinically relevant changes that occur during the trial in the AE section of the CRF. The following safety and tolerability evaluations will be performed:

Tolerability

Local: evaluation of the injection site: The investigator will evaluate the site of injection for redness, pain, swelling, and induration and functional limitation at the time points indicated in the events schedule (Figure 18Figure 18).

Redness, swelling and induration will be evaluated and recorded on the CRF as: 0 = absent, 1 = mild, 2 = moderate, 3 = severe. If present, the extent of the reaction will be measured in mm. Pain will be recorded, after questioning the subjects, by means of a Visual Analogue Scale (VAS from 0 to 100). The presence of abscess, ulceration or necrosis will also be evaluated, measured and adequately documented.

Focal: evaluation of the hilar lymph nodes for inflammation: An un-contrasted thoracic computerised tomographic scan will be performed prior to first vaccine inoculation and four weeks after the last one (2nd vaccine inoculation) to evaluate the change in size of the hilar lymph nodes.

Systemic: body temperature ≥38ºC, asthenia, sweating, malaise, headache, dizziness, nausea, myalgia, arthralgia, rash and generalised pruritus will be reported.

Adverse events will be reported by the subject for the duration of the trial both spontaneously and after questioning.

Vital Signs

Blood pressure (systolic and diastolic), pulse, respiratory rate and body temperature will be assessed for 5 minutes post vaccination.

Physical Examination

Full physical examination will be performed according to the events schedule. Clinical significant findings will be documented as AEs / MH as applicable.

ECG

The ECG tracing will be obtained according to the events schedule. The investigator will assess, sign, date, and comment on each tracing. The following ECG parameters will be measured: Heart rate, PR, QRS, QT and QTc (Bazett) intervals. Any other anomaly on the ECG (such as U wave, ischaemia, rhythm and conduction disturbances) will be evaluated by the investigator.

Laboratory Tests

Blood samples for serum chemistry and haematology and urine sample for urinalysis will be taken under fasting conditions for evaluation of laboratory safety parameters. Safety laboratory parameters are listed below:

Haematology

Hematocrit, haemoglobin, red blood cells (RBC) count, mean corpuscular volume (MCV), mean corpuscular haemoglobin (MCH), mean corpuscular haemoglobin concentration (MCHC), white blood cell (WBC) count, leukocytes, platelet count.

Biochemistry

In serum: sodium, potassium, calcium, aspartate amino transferase activity (AST), alanine amino transferase activity (ALT), alkaline phosphatase (ALP), gamma-glutamyl transferase (GGT), creatine kinase (CK), glucose, creatinine, urea, total bilirubin, total serum proteins, albumin, uric acid.

Urinalysis*: Urinalysis will be performed on all subjects using a Uricheck® or equivalent urine testing device. The following will be captured: protein, bilirubin (if pathological, an urine sediment analysis will be performed), blood, urobilinogen.

*If any abnormalities are noted on urinalysis, microscopy on the urine sediment will be performed.

The HIV antibodies will be tested at pre-screen. Additionally, Hepatitis B surface Antigen (HBsAg), Hepatitis C virus (HCV) antibodies and HIV antibodies (if necessary) will be tested at screening.

Any clinically significant abnormalities relating to the safety laboratory parameters persisting at the end of the trial will be followed up by the investigator until resolution or until reaching a clinically stable endpoint.

7.2 Procedures for Eliciting Reports of and for Recording and Reporting Adverse Event and Intercurrent Illnesses

The original terms used in the CRFs by investigators to identify AEs will be coded using the Medical Dictionary for Regulatory Activities (MedDRA). Special attention will be given to those subjects who have discontinued treatment due to an AE or who experienced a severe or a serious AE.

The present clinical trial will be conducted in accordance to Standard Operating Procedures (SOPs) in conformity with regulatory requirements worldwide to ensure appropriate reporting of safety information. Adverse events will be reported from the time the subject signs the informed consent form until the subject completes the last trial procedure.

7.2.1 Adverse Event Definitions and Classifications

Adverse Event

An AE, as defined by the International Conference on Harmonisation (ICH), is any untoward medical occurrence in a clinical trial subject administered a pharmaceutical product. An AE does not necessarily have a causal relationship with the treatment. An AE can therefore be any unfavorable and unintended sign (including an abnormal laboratory finding), symptom, or disease temporally associated with the use of a medicinal (investigational) product, whether or not related to the medicinal (investigational) product.

This includes any occurrence that is new in onset or aggravated in severity or frequency from the baseline condition, or abnormal results of diagnostic procedures, including laboratory test abnormalities.

Serious Adverse Event

A SAE as defined by ICH is any untoward medical occurrence that at any dose meets any of the following conditions:

- results in death,
- is life-threatening; i.e., the subject was at risk of death at the time of the event; this does not refer to an event that hypothetically might have caused death if it were more severe,
- requires inpatient hospitalization or prolongation of existing hospitalization,
- results in persistent or significant disability/incapacity or
- there is a congenital anomaly/birth defect.

Note: Medical and scientific judgment should determine whether an adverse event should be classified as serious in other situations. Adverse events classified as serious may include important medical events that are not immediately life threatening or could result in death or hospitalization but may jeopardize the subject or require intervention to prevent one of the outcomes listed above. Any AE is considered a SAE if it is associated with clinical signs or symptoms judged by the investigator to have a significant clinical impact.

Unlisted (Unexpected) Adverse Event

An AE is unexpected when its nature or severity is not consistent with the applicable product information. For an investigational product, the expectedness of an AE will be determined by whether or not it is listed in the Investigator's Brochure. For a comparator product with a marketing authorization, the expectedness of an AE will be determined by whether or not it is listed in the Investigator's Brochure.

Associated With the Use of the Drug

The relationship of an adverse event will be assessed independently for INH and the IMP: RUTI® vaccine.

An AE is considered associated with the use of the drug if the association is possible, probable, or very likely according to the definitions listed below:

Attribution Definitions

- Not related

An AE that is not related to the use of the drug.

- Doubtful

  An AE for which an alternative explanation is more likely, e.g., concomitant drug(s), concomitant disease(s), or the relationship suggests that a causal relationship is unlikely.

- Possible

  An AE that might be due to the use of the drug. An alternative explanation, e.g., concomitant drug(s), concomitant disease(s), is inconclusive. The relationship is reasonable; therefore, the causal relationship cannot be excluded.

- Probable

  An AE that might be due to the use of the drug. A relationship to the trial drug is suggested. An alternative explanation, e.g., concomitant drug(s), concomitant disease(s), is less likely.

- Very likely

  An AE that is listed as a possible adverse reaction and cannot be reasonably explained by an alternative explanation, e.g., concomitant drug(s), concomitant disease(s)

Severity Grade Description

Mild	1	Symptoms causing no or minimal interference with usual social and functional activities
Moderate	2	Symptoms causing greater than minimal interference with usual social and functional activities
Severe	3	Symptoms causing inability to perform usual social and functional activities

Outcome

Each adverse event will be characterized according to the outcomes:

Outcome	Description
Resolved	The subject has fully recovered from the event with no observable residual effects.
Improved	The effects of the event are improving but have not returned to baseline.
Persisting	The effects of the event are still present and changing (may be worsening). The event is not considered stabilized or resolved.
Resolved with sequelae	The subject has fully recovered from the event with some observable residual effects.
Fatal	The event was the primary cause of death (may or may not be the immediate cause of death).
Unknown	The event outcome is unknown.

Death is an outcome of an event and not an event per se. However, sudden death or death due to unexplainable cause(s) is to be reported as an adverse event, but follow-up will be pursued until cause of death is determined.

Action Taken with Drug (INH and RUTI ® placebo)

Action taken with study drug in relation to each adverse event will be characterized as follows:

- Drug withdrawn [i.e., study drug(s) discontinued permanently]
- Drug withheld [e.g., study drug(s) not administered for 1 dosing visit or period of time]
- Unknown
- None

7.2.2 Adverse Event Reporting Procedures

All Adverse Events

All AEs will be reported from the time a signed and dated informed consent form is obtained until completion of the last trial-related procedure. The AEs meeting the definition of SAEs must

be reported using the SAE Form (see Serious Adverse Events below), including SAEs spontaneously reported to the investigator within 30 days after the subject has completed the trial (including post-trial follow-up). The sponsor will evaluate any safety information that is spontaneously reported by an investigator beyond the time frame specified in the protocol.

All AEs that meet the definition of a SAE will be reported as SAEs, regardless of whether they are protocol-specific measurements.

All AEs, regardless of seriousness, severity, or presumed relationship to trial drug, must be recorded using medical terminology in the source documents and the CRF. Whenever possible, diagnoses should be given when signs and symptoms are due to a common aetiology (e.g., cough, runny nose, sneezing, sore throat, and head congestion should be reported as "upper respiratory infection"). Investigators must record their opinion concerning the relationship of the AE to trial drug in the CRF. All measures required for AE management must be recorded in the source documents and reported according to sponsor instructions.

The sponsor assumes responsibility for appropriate reporting of AEs to the regulatory authorities. The sponsor will also report all SAEs that are unlisted and associated with the use of the drug to the investigator. The investigator (or sponsor where required) must report these SAEs to the appropriate Independent Ethics Committee/Institutional Review Board (IEC/IRB) that approved the protocol unless otherwise required and documented by the IEC/IRB.

Subjects must be provided with a "trial card" indicating the name of the investigational product, the trial number, the investigator's name, a 24 hour emergency contact number, and, if applicable, excluded concomitant medications.

Serious Adverse Events

All SAEs occurring during the clinical trial must be reported to the appropriate sponsor contact person by investigational staff within 24 hours of their knowledge of the event.

Information regarding SAEs will be transmitted to the sponsor using the SAE Form, which must be signed by a physician on the investigational staff. The initial report of a SAE should be done by fax. If necessary, it may be done by telephone. Subsequent to a telephone report of a SAE, the signed SAE Form must be completed and transmitted to the sponsor within one working day.

7.3 Type and Duration of the Follow-up of Subjects after Adverse Events

Any clinically significant abnormalities or AEs detected during the trial or not resolved upon discontinuation of the subject's participation in the trial, or by the end of the trial, must be followed up until any of the following occurs:

- the event resolves
- the event stabilizes
- the event returns to baseline, if a baseline value is available
- the event can be attributed to agents other than the trial drug or to factors unrelated to trial conduct
- when it becomes unlikely that any additional information can be obtained (subject or health care practitioner refusal to provide additional information, lost to follow-up after demonstration of due diligence with follow-up efforts)

Pregnancy

Pregnancy alone is not considered an adverse event. However, any report of pregnancy that occurs in a female subject within 1 month after the end of visit, even if the subject is withdrawn from study, must be reported on the Pregnancy Form. The investigator must follow the pregnancy either to termination or to term and will collect data on both maternal and fetal outcome. All pregnancy outcomes will be recorded on the Pregnancy Form.

Normal outcomes will be communicated to the Sponsor within 30 calendar days of birth/delivery. Abnormal pregnancy outcomes and/or any adverse event for the child or fetus (including miscarriage), will also be recorded in the adverse event CRF and on the SAE form. The associated SAE form should be sent to the Sponsor. An adverse event CRF and SAE form as appropriate will be completed if the subject sustains an event.

Overdose

Overdose is defined as anything outside the defined or prescribed use as applicable for the study drug and trial design. Occurrences of overdose should be reported to the Sponsor for tracking purposes. Additional instructions for reporting overdose information will be provided by the Sponsor at the time of notification.

8. ACTIVE TB SURVEILLANCE [9, 10, 11]

8.1 Diagnosis of Pulmonary TB

8.1.1 Clinical Features

Accordingly with the WHO Clinical Manual for HIV and TB, the most important symptoms in the diagnosis of pulmonary TB (PTB) are the following:

- Cough for more than 2 or 3 weeks
- Sputum production
- Weight loss

Patients with PTB may also have other symptoms. These may be respiratory or constitutional (general or systemic).

Respiratory: chest pain, haemoptysis, breathlessness

Constitutional: fever, night sweats, tiredness, loss of appetite, secondary amenorrhoea.

Weight loss and fever are more common in HIV+ PTB patients than in those who are HIV-. Conversely, cough and haemoptysis are less common in HIV+ PTB patients than in those who are HIV-. This is probably because there is less cavitation, inflammation and endobronchial irritation in HIV+ patients.

Therefore, a PTB suspect is a patient with a cough for more than 2 to 3 weeks, and must submit sputum samples for smear microscopy and mycobacterial culturing.

8.1.2 Chest X-Ray in Diagnosis

No chest X-ray (CXR) pattern is absolutely typical of PTB, especially with underlying HIV infection. The table below (Table 7) shows so-called "classical" and "atypical" CXR patterns. The classical pattern is more common in HIV- patients, and the atypical pattern in HIV+ patient.

Table 7 "Classical" and "atypical" chest X-ray patterns

CLASSICAL PATTERN	ATYPICAL PATTERN
Upper lobe infiltrates	Interstitial infiltrates (especially lower zones)
Bilateral infiltrates	Intrathoracic lymphadenopathy
Cavitation	No cavitation
Pulmonary fibrosis and shrinkage	No abnormalities

8.1.3 Confirmatory Diagnosis by Smear Microscopy and Mycobacterial Culturing

A PTB suspect should submit three sputum samples. Secretions build up in the airways overnight. So an early morning sputum sample is more likely to contain TB bacilli than one taken later in the day. It may be difficult for an outpatient to provide three early morning sputum samples. Therefore sputum samples will be provided as follows:

Day 1	Sample 1	Patient provides an "on-the-spot" sample under supervision when presenting to the health facility. A sputum container will be given to the patient to take home for an early morning sample the following morning
Day 2	Sample 2	Patient brings an early morning sample
	Sample 3	Patient provides another "on-the-spot" sample under supervision

Specimens will be first processed to remove viable organisms other than mycobacteria through a specific decontamination process. Smear examination and liquid culturing will be done. *Mycobacterium tuberculosis* complex identification and sensitivity testing will be ensured through specific molecular biology testing.

8.2 Diagnosis of Extrapulmonary TB

Extrapulmonary (EPTB) can occur at any age. HIV+ adults are particularly susceptible. Up to 25% of TB cases may present with EPTB. The common forms of EPTB associated with HIV are the following: lymphadenopathy, pleural effusion, pericardial disease, miliary TB, and meningitis. Many patients with EPTB also have coexistent pulmonary TB.

Definitive diagnosis of EPTB is often difficult. Diagnosis may be presumptive, after exclusion of other conditions. Patients usually present with constitutional features (fever, night sweats, weight loss) and local features related to the site. The certainty of diagnosis will depend of the diagnostic tools including image devices and organ sampling. Thus lymphadenopathy will require lymph node needle aspirate or biopsy for cytology/histology and mycobacterial stain and culturing; miliary TB will require CXR and mycobacterial stain and culturing of sputum, cerebrospinal fluid, bone marrow, liver or blood; Pericardial disease would require CXR, ECG, Echocardiograpy and pericardiocentesis, also to be stained and cultured; Pleural disease, would require CXR, pleural aspiration and pleural biopsy, etc.

9. STATISTICS (BIOMETRIC PLAN)

The biometric plan describes the statistical analysis as it is foreseen when the trial is being planned. If circumstances should arise during the trial rendering this analysis inappropriate, or if in the meantime improved methods of analysis should come to light, different analyses may be performed. Any deviations from the biometric plan, reasons for such deviations and all alternative or additional statistical analyses that may be performed, will be described and explained in the detailed Statistical Analysis Plan (SAP) that will be prepared prior to unblinding, as well as in the clinical trial report. The trial statistician in collaboration with the principal investigators (or designees) and ARCHIVEL FARMA, S.L. will be responsible for the preparation of the SAP. Complete procedures regarding the statistical analysis of all variables, the handling of missing values, the determination of trial populations and other details, including the interim analyses, will be detailed in the SAP. The SAP will be finalized before database lock and the clinical trial will be analyzed according to the SAP after database lock.

The creation of tables and listings and the statistical analyses will be performed using SAS® version 9.1 or higher (8).

9.1 Data Management

An integrated and validated data management system with remote data entry capabilities and access restrictions will be used for the informatization of the trial data. The MACRO® (12) software has been used for a number of pharmaceutical industries and academic research organizations and it is compliant with Good Clinical Practice (GCP) (13), general recommendations on data management (14), and with regulatory requirements for validation (15, 16). The data will be entered by the local trial investigators using individual login accesses and the system will check for the data consistency and range checks; automatic queries will be raised to be solved by the trial investigators until the database will be considered clean. Data changes due to query forms raised by the monitoring will be also included into the database and identified in the system audit logs. All filters, consistency rules and range checks will be applied according to a data validation plan. Any system access and data changes will be tracked (including old and new values, date-time, user and justification). The data will be available for the monitoring review and for auditing and inspection procedures.

9.2 Immunogenicity Data Handling

Immunogenicity data generated will be transferred directly to the trial database and appropriate verifications of the data transfer will be performed to ensure the validity of this process. Once all data has been incorporated into the database, a Blind Data Review (BDR) meeting will be held in order to check the quality of the data and to determine the different trial analysis populations. After the BDR meeting and determination of the trial analysis populations, the database will be locked and the final data will be transferred to the trial statistician.

For the interim analysis partial closures of the database with partially validated data will be prepared with an appropriate log and tracking of the process.

9.3 Statistical Methods

9.3.1 Safety/Tolerability Parameters

Adverse events will be listed and summarized by system organ class and preferred terms for each treatment group using descriptive statistics.

Tolerability assessments will be listed per patient and summarized using frequency counts or descriptive statistics, as appropriate.

Physical examination results will be summarized using frequency counts and shift tables.

Vital signs, laboratory parameters and ECG parameters will be listed (both absolute and change from baseline results) and summarized per treatment group and visit, for absolute and change from baseline results, using descriptive statistics.

Clinical history results, TST and QuantiFeron, chest X-ray, chest CT scan, CD4 counts, and viral load test results will be listed.

Concomitant medication will be coded using the World Health Organization Drug Reference List (WHO-DRL) and Anatomic Therapeutic Chemical (ATC) classification, listed per patient and summarized per treatment group.

9.3.2 Immunogenicity Parameters

The immunogenicity parameters (IFN-γ Spot Forming Units in peripheral blood mononuclear cells (PBMCs), IFN-γ concentration in whole blood and antibody concentration in sera) will be listed per patient and summarized using descriptive statistics by treatment group and visit. The difference between treatments effect will be studied by comparing the measurement of the

immunogenicity parameters. An analysis of covariance (ANCOVA) will be used, with treatment as main effect and the baseline value as covariate. The data and comparisons between the treatments will be described by the least square means adjusted in accordance to the ANCOVA analysis.

9.4 Interim Analysis

The interim analyses, to be performed for each DSMC review, will also be described in the SAP.

These interim analyses will be conducted by an independent statistician to guarantee the blinding of the rest of the trial personnel and to avoid any influence on the conduct of the clinical trial. Un-blinded results will be generated by the interim analyses statistician and these results will be exclusively sent to the DSMC and the Ethics Committee.

ARCHIVEL FARMA, S.L., the sponsor, trial investigators and the rest of the trial personnel, except the interim analyses statistician and the DSMC will be kept blinded to the clinical trial results. The DSMC will only communicate the decision to continue, modify or stop the clinical trial.

9.5 Trial Supervision and Monitoring

The DSMC will oversee the progress of the clinical trial.

The monitor will ensure that the trial is conducted, recorded and reported in accordance to the protocol and SOPs and will check the database against the source documents such as clinical records and CRFs. The principal investigators, by signing the protocol, and the volunteers, by signing informed consent, agree that the monitor may consult and/or copy source records (clinical notes and laboratory values) to accomplish their monitoring tasks. Such information will be treated as strictly confidential and under no circumstances will it be made publicly available. The monitoring will adhere to GCP guidelines.

Data which should be verifiable from source documents for trial volunteers includes, but is not limited to:

1. Documentation of any existing conditions or past conditions relevant to the eligibility,
2. Signed informed consent forms,
3. Dates of visits including dates of vaccinations,

4. Reported laboratory results,
5. All AEs, and
6. Concomitant medications.

9.6 Number of Subjects Planned to be Enrolled (Sample Size)

A total of 96 subjects will be enrolled: 48 HIV negative (HIV-) and 48 HIV positive (HIV+) subjects. The subjects of both phases of the clinical trial will be randomized to one of four treatment groups as presented in Table 4~~Table 4~~ (Section 3.3).

Sample size is not based on a statistical rationale. Since this is an exploratory safety and tolerability phase II trial, no formal sample size calculations have been made. However, the numbers foreseen in this trial, 12 subjects group, are comparable with the standard sample sizes used in these studies (Table 8).

Table 8 In the following tables we give the probabilities of detecting an adverse event between 0.1 to 10% and the exact confidence intervals for the whole set exposed to the vaccine (N=32)

Event rate	N	Probability to detect al least:		
		1 Event	2 Events	3 Events
0.1%	72	7.0%	0.2%	0.0%
0.5%	72	30.3%	5.1%	0.6%
1.0%	72	51.5%	16.2%	3.6%
2.0%	72	76.7%	42.3%	17.5%
3.0%	72	88.8%	64.0%	36.7%
4.0%	72	94.7%	78.8%	55.4%
5.0%	72	97.5%	88.1%	70.5%
6.0%	72	98.8%	93.5%	81.4%
7.0%	72	99.5%	96.5%	88.8%
8.0%	72	99.8%	98.2%	93.4%
9.0%	72	99.9%	99.1%	96.3%
10.0%	72	99.9%	99.5%	97.9%

**N**	**n**	**%**	**95%CI**	
72	0	0.0%	0.0%	5.1%
	1	1.4%	0.2%	7.5%
	2	2.8%	0.8%	9.6%
	3	4.2%	1.4%	11.5%
	4	5.6%	2.2%	13.4%
	5	6.8%	3.0%	15.1%

9.7 Criteria for the Termination of the Trial

The trial is considered completed with the last visit of the last subject completing the trial. The final data from the investigational centre will be sent to ARCHIVEL FARMA, S.L. in the time frame specified in the Clinical Trial Agreement.

An investigational centre is considered closed when all required documents and trial supplies have been collected and a centre closure visit has been performed.

The sponsor reserves the right to close the investigational centre or terminate the trial at any time. The investigator may initiate centre closure at any time, provided there is reasonable cause and sufficient notice is given in advance of the intended termination.

Reasons for the early closure of an investigational centre by the sponsor or investigator, or termination of a trial by the sponsor, may include but are not limited to:

- failure of the investigator to comply with the protocol, the sponsor's procedures, or GCP guidelines
- safety concerns
- inadequate recruitment of subjects by the investigator, trial endpoint or subject target reached.

9.8 Procedure for Accounting for Missing, Unused, and Spurious Data

No imputation will be performed in this trial and the analysis will be done with the Available Data Only approach. Repeated laboratory samples per time point will be handled according the following strategy:

- Before treatment: Last observation
- After treatment: First observation
- Missing/Non-interpretable values: Value excluded from analysis

9.9 The Selection of Subjects to be Included in the Analyses

There are two different analyses populations considered for the trial:

1. The Safety Population will include all the subjects who received the one month pre-treatment with INH.

2. The Per Protocol (PP) Population is defined as randomized subjects who received trial medication, completed the trial and do not have any major protocol deviations.

The detailed reasons to exclude the subjects from the PP Population will be specified in the SAP and discussed and documented during the BDR meeting.

10. DIRECT ACCESS TO SOURCE DATA/DOCUMENTS

The sponsor will ensure that the investigators will permit trial-related monitoring, audits, IRB-IECIRB/IEC review, and regulatory inspections, providing direct access to source data and documents.

11. QUALITY CONTROL AND QUALITY ASSURANCE

To ensure the quality and reliability of data gathered and the ethical conduct of the clinical trial, SOPs will be applied for all trial procedures. Regular monitoring and an independent audit of the clinical trial will be performed according to GCP.

11.1 Data Quality Control

Steps to be taken to ensure the accuracy and reliability of data include the selection of qualified investigators and appropriate trial centres, review of protocol procedures with the investigator and associated personnel before the trial, and periodic monitoring visits by the sponsor.

Case report form completion training will be conducted with trial personnel before the start of the trial. The sponsor's monitor will review data for accuracy and completeness during on-site monitoring visits and after his or her return to the sponsor's office; any discrepancies will be resolved with the investigator or designee, as appropriate. The data will be entered into the clinical trial database and verified for accuracy.

11.2 Monitoring

The sponsor will assign monitors who will perform on-site monitoring visits as frequently as necessary and in compliance with the ICH-GCP Section 5.18.4.

Findings from this review of CRFs and source documents will be discussed with the investigational staff. The sponsor expects that, during monitoring visits, the relevant investigational staff will be available, the source documentation will be accessible, and a suitable environment will be provided for review of trial-related documents. The monitor will meet with the investigator on a regular basis during the trial to provide feedback on the trial conduct.

On-Site Audits/Inspections

Representatives of the sponsor's quality assurance department may visit the centre to conduct an audit of the trial in compliance with regulatory guidelines and company policy. These audits will

require access to all trial records, including source documents, for inspection and comparison with the CRFs. Subject privacy must, however, be respected.

Inspections may be conducted by agents of any regulatory body either as part of a national GCP compliance program or in support of the review of a regulatory submission. The investigator should immediately notify the sponsor if they have been contacted by a regulatory agency concerning an upcoming inspection.

12. ETHICS

12.1 Investigator Responsibilities

The investigator is responsible for ensuring that the clinical trial is performed in accordance with the protocol, current ICH guidelines on GCP, and applicable regulatory and applicable regulatory and legal requirements. GCP is an international ethical and scientific quality standard for designing, conducting, recording, and reporting studies that involve the participation of human subjects. Compliance with this standard provides public assurance that the rights, safety, and well being of trial subjects are protected, consistent with the principles that originated in the Declaration of Helsinki, and that the clinical trial data are credible.

12.2 Independent Ethics Committee or Institutional Review Board (IE/IRB)

Before the start of the trial, a written favourable opinion or approval must be received from the IEC/IRB. To achieve this, the investigator or the sponsor will submit to the IEC/IRB, as required by local regulations, current and complete copies of relevant documents.

The written favourable opinion or approval must be dated and must clearly identify the documents reviewed, which should include all the documents that the IEC/IRB requests to fulfil its obligation.

During the trial, the investigator or sponsor, as required, will submit the following for IEC/IRB review or opinion/approval:

- revisions or updates of documents previously submitted to the IEC/IRB.
- relevant new information or documents, as required.
- summaries of the status of the trial (at least annually or at intervals stipulated in guidelines of the IEC/IRB).
- reports of AEs that are serious, unlisted, and associated with the investigational drug.
- deviations from or changes to the protocol to eliminate immediate hazards to the subjects.

When and where required by local regulations, before implementation of any change, protocol amendments and revised documents must receive IEC/IRB favourable opinion or approval.

The IEC/IRB will be given official notification of the trial completion.

12.3 Informed Consent

Each subject must give written consent according to local requirements after receiving a full explanation of the nature of the trial. The consent form must be signed before performance of any trial-related activity. The consent form that is used must be approved by both the sponsor and by the reviewing IEC/IRB. The informed consent process and form should be in accordance with principles that originated in the Declaration of Helsinki, current ICH GCP guidelines, applicable regulatory requirements, and sponsor policy.

Before a subject's entry into the trial, the investigator or an authorized member of the investigational staff must explain to the potential subject the aims, methods, and potential hazards of the trial, and any discomfort it may entail. Subjects will be informed that their participation is voluntary and that they may withdraw consent to participate at any time. They will be informed that choosing not to participate will not affect the care a subject will receive. Finally, they will be told that the investigator will maintain a subject enrolment log for the purposes of long-term follow-up if needed and that their records may be accessed by health authorities and authorized sponsor staff without violating the confidentiality of the subject, to the extent permitted by the applicable law(s) or regulations. By signing the informed consent form the subject is authorizing such access, and agrees to be re-contacted after trial completion, by health authorities and authorized sponsor staff, for the purpose of obtaining consent for additional safety evaluations if needed.

Subjects will be given sufficient time to read the informed consent form in their language of choice and the opportunity to ask questions. After this explanation and before entry into the trial, consent should be appropriately recorded by means of the subject's personally dated signature. After the consent is obtained, a copy of the informed consent form must be given to the subject.

12.4 Privacy of Personal Data

The collection and processing of personal data from subjects enrolled in this trial will be limited to those data that are necessary to investigate the safety, and the effectiveness of the treatment regimen involving the investigational product used in this trial. These data will be collected and processed with adequate precautions to ensure confidentiality and compliance with applicable data privacy protection laws and regulations. A unique identification code will be assigned by the investigators to each trial subject to protect the subject's identity and used instead of the subject's name when the investigators would report AEs and/or other trial related data.

The informed consent obtained from the subject includes explicit consent for the processing of personal data and for the investigator to allow direct access to his or her original medical records for trial-related monitoring, audit, IRB/IEC review, and regulatory inspection. This consent also addresses the transfer of the data to other entities and to other countries.

The subject has the right to request through the investigator access to his or her personal data and the right to request rectification of any data that are not correct or complete. Reasonable steps should be taken to respond to such a request, taking into consideration the nature of the request, the conditions of the trial, and the applicable laws and regulations.

Appropriate technical and organizational measures to protect the personal data against unauthorized disclosures or access, accidental or unlawful destruction, or accidental loss or alteration must be put in place. Sponsor or designee personnel whose responsibilities require access to personal data agree to keep the identity of trial subjects confidential.

13. DATA HANDLING AND RECORD KEEPING

At a minimum, source documentation must be available to substantiate subject identification, eligibility, and participation; proper informed consent procedures; dates of visits; adherence to protocol procedures; records of safety parameters; adequate reporting and follow-up of AEs; administration of concomitant therapy; drug receipt/dispensing/return records; trial drug administration information; and date of subject completion, discontinuation from treatment, or withdrawal from the trial, and the reason if appropriate. Specific items required as source documents will be reviewed with the investigator before the trial.

The data will be recorded directly in the CRF. However, at minimum the following data must be maintained in source documentation: subject identification, subject eligibility, exposure to trial drug, AEs, and concomitant therapy. It is recommended that the author of an entry in the source documents be identifiable. Worksheets may be used for the capture of some data to facilitate completion of the CRF. Any such worksheets will become part of the subjects' source documentation.

Following the ICH-GCP guidelines, direct access to source documentation (medical records) must be allowed for the purpose of verifying that the data recorded in the CRF are consistent with the original source data.

13.1 Case Report Form Completion

An CRF will exist for each subject. The investigator must verify that all data entries in the CRFs are accurate and correct. All CRF entries, corrections, and alterations must be made by the investigator or other authorized trial-centre personnel.

13.2 Record Retention

In compliance with the ICH/GCP guidelines, the investigator/institution will maintain all source documents that support the data collected from each subject, as well as all trial documents as specified in ICH/GCP Section 8, Essential Documents for the Conduct of a Clinical Trial, and all trial documents as specified by the applicable regulatory requirement(s). The investigator/institution will take measures to prevent accidental or premature destruction of these documents.

Essential documents must be retained until at least two years after the last approval of a marketing application in an ICH region and until there are no pending or contemplated marketing

applications in an ICH region or until at least two years have elapsed since the formal discontinuation of clinical development of the investigational product. These documents will be retained for a longer period if required by the applicable regulatory requirements or by an agreement with the sponsor. It is the responsibility of the sponsor to inform the investigator/institution as to when these documents no longer need to be retained.

If the responsible investigator retires, relocates, or for other reasons withdraws from the responsibility of keeping the trial records, custody must be transferred to a person who will accept the responsibility. The sponsor must be notified in writing of the name and address of the new custodian.

Under no circumstance shall the investigator relocate or dispose of any trial documents before having obtained written approval from the sponsor.

14. INSURANCE

Insurance cover has been arranged in compliance with the ABPI guidelines to indemnify the subjects in the event of death or any deterioration in health or well-being caused by participation in the study.

The certificate of insurance will be kept in the Trial Master File

15. PUBLICATION POLICY

All information, including but not limited to information regarding RUTI® or the sponsor's operations (e.g., patent application, formulas, manufacturing processes, basic scientific data, prior clinical data, formulation information) supplied by the sponsor to the investigator and not previously published, and any data including research data, generated as a result of this trial, are considered confidential and remains the sole property of the sponsor. The investigator agrees to maintain this information in confidence and to use this information only to conduct this trial, and will not use it for other purposes without the sponsor's prior written consent.

The investigators understand that the information developed in the clinical trial will be used by the sponsor in connection with the continued development of RUTI®, and thus may be disclosed as required to other clinical investigators or regulatory agencies. To permit the information derived from the clinical studies to be used, the investigator is obligated to provide the sponsor with all data obtained in the trial.

The results of the trial will be reported in a Clinical Trial Report under the supervision of the sponsor and will contain all data from all investigational centres. Results of any analyses performed after the Clinical Trial Report has been issued will be reported in a separate report and will not require a revision of the Clinical Trial Report. Trial subject identifiers will not be used in publication of results. Any work created in connection with performance of the trial and contained in the data that can benefit from copyright protection (except any publication by the investigator as provided for below) shall be the property of the sponsor as author and owner of copyright in such work.

The sponsor shall have the right to publish such data and information. If an investigator wishes to publish information from the trial, a copy of the manuscript must be provided to the sponsor for review at least 60 days before submission for publication or presentation. Expedited reviews will be arranged for abstracts, poster presentations, or other materials. If requested by the sponsor in writing, the investigator will withhold such publication for up to an additional 60 days to allow for filing of a patent application. In the event that issues arise regarding scientific integrity or regulatory compliance, the sponsor will review these issues with the investigator. The sponsor will not mandate modifications to scientific content and does not have the right to suppress information. Authorship of publications resulting from this trial will be based on generally accepted criteria for major medical journals.

16. REFERENCE LIST

1. Note for Guidance on Good Clinical Practice (CPMP/ICH/135/95). The European Agency for the Evaluation of Medicinal Products (EMEA) 2002;1-59.
2. World Health Association Declaration of Helsinki. Recommendations Guiding Physicians in Biomedical Research involving Human Subjects. Adopted by the 18th WMA General Assembly, Helsinki, Finland, June 1964, and amended by the 29th WMA General Assembly, Tokyo, Japan, October 1975, 35th WMA General Assembly, Venice, Italy, October 1983, 41st WMA General Assembly, Hong Kong, September 1989, 48th WMA General Assembly, Somerset West, Republic of South Africa, October 1996, 52nd WMA General Assembly, Edinburgh, Scotland, October 2000, 53rd WMA General Assembly, Washington 2002 (Note of Clarification on Paragraph 29 added), 55th WMA General Assembly, Tokyo 2004 (Note for Clarification on Paragraph 30 added), and 59th WMA General Assembly, Seoul, October 2008.
3. Cardona PJ. RUTI: a new chance to shorten the treatment of latent tuberculosis infection. *Tuberculosis (Edinb)*. 2006 May-Jul;86(3-4):273-89. Epub 2006 Mar 20.
4. Domingo M, Gil O, Serrano E, Guirado E, Nofrarias M, Grassa M, Cáceres N, Pérez B, Vilaplana C, Cardona PJ. Effectiveness and safety of a treatment regimen based on isoniazid plus vaccination with Mycobacterium tuberculosis cells' fragments: field-study with naturally Mycobacterium caprae-infected goats. *Scand J Immunol.* 2009 Jun;69(6):500-7.
5. Comstock GW. How much isoniazid is needed for prevention of tuberculosis among immunocompetent adults? *Int J Tuberc Lung Dis.* 1999 Oct;3(10):847-50.
6. Harris A, Maher D, Graham. TB/HIV a clinical manual. 2nd Edition. WHO. 2004.
7. ARCHIVEL FARMA, s.l. Investigator's Brochure. RUTI® vaccine 50 µg FCMtb powder for suspension for injection. 2nd Edition, August 2009.
8. SAS® Version 9.1 of the SAS System for Personal Computers. Copyright © 2002-2003. SAS Institute Inc. SAS and all other SAS Institute Inc. product or service names are registered trademarks or trademarks of SAS Institute Inc., Cary, NC, USA
9. Schaaf HS, Zumla AI. Tuberculosis. A comprehensive clinical reference. Saunders Elsevier. Europe. 2009.
10. WHO. TB/HIV a clinical manual. World Health Organization. Geneva. 2nd Edition. 2004.

11. Centers for Disease Control and Prevention. *1993 revised classification system for HIV infection and expanded surveillance case definition for AIDS among adolescents and adults .* *MMWR Recomm Rep.* 1992 Dec 18;41(RR-17):1-19.

12. Note for Guidance on Good Clinical Practice. ICHE6.

13. Good Clinical Data Management Practice, Version 4, Society for Clinical Data Management (SCDM), October 2005.

14. EMEA. Reflection paper on expectations for electronic source documents used in clinical trials. London, 17 October 2007. Doc. Ref. EMEA/505620/2007

15. FDA. Guidance for Industry. Part 11, Electronic Records; Electronic Signatures – Scope and Application, August 2003.

16. FDA. Guidance for Industry. Computerized Systems Used in Clinical Investigations (May 2007).

17. FDA. Guidance for Industry. Part 11, Electronic Records; Electronic Signatures – Scope and Application (August 2003).

16.1 Bibliography

Advisory Council for the Elimination of Tuberculosis (ACET). Development of new vaccines for tuberculosis. Recommendations of the Advisory Council for the Elimination of Tuberculosis (ACET). *MMWR Recomm Rep.* 1998 Aug 21;47(RR-13):1-6.

American Thoracic Society. Targeted tuberculin testing and treatment of latent tuberculosis infection. *Am J Respir Crit Care Med.* 2000 Apr;161(4 Pt 2):S221-47.

Andersen P, Doherty TM. TB subunit vaccines--putting the pieces together. *Microbes Infect.* 2005 May;7(5-6):911-21. Epub 2005 Apr 14.

Andersen P, Doherty TM. The success and failure of BCG - implications for a novel tuberculosis vaccine. *Nat Rev Microbiol.* 2005 Aug;3(8):656-62.

Brock I, Weldingh K, Lillebaek T, Follmann F, Andersen P. Comparison of tuberculin skin test and new specific blood test in tuberculosis contacts. *Am J Respir Crit Care Med.* 2004 Jul 1;170(1):65-9. Epub 2004 Apr 15.

Cardona PJ, Amat I, Gordillo S, Arcos V, Guirado E, Díaz J, et al. Immunotherapy with fragmented Mycobacterium tuberculosis cells increases the effectiveness of chemotherapy against a chronical infection in a murine model of tuberculosis. *Vaccine.* 2005 Feb 3;23(11):1393-8.

Cardona PJ, Gordillo S, Díaz J, Tapia G, Amat I, Pallarés A, et al. Widespread bronchogenic dissemination makes DBA/2 mice more susceptible than C57BL/6 mice to experimental aerosol infection with Mycobacterium tuberculosis. *Infect Immun.* 2003 Oct;71(10):5845-54.

Cardona PJ, Ruiz-Manzano J. On the nature of Mycobacterium tuberculosis-latent bacilli. *Eur Respir J.* 2004 Dec;24(6):1044-51.

Cardona PJ. New insights on the nature of latent tuberculosis infection and its treatment. *Inflamm Allergy Drug Targets.* 2007 Mar;6(1):27-39.

Ciaramella A, Cavone A, Santucci MB, Garg SK, Sanarico N, Bocchino M, et al. Induction of apoptosis and release of interleukin-1 beta by cell wall-associated 19-kDa lipoprotein during the course of mycobacterial infection. *J Infect Dis.* 2004 Sep 15;190(6):1167-76. Epub 2004 Aug 11.

Cole ST, Brosch R, Parkhill J, Garnier T, Churcher C, Harris D, et al. Deciphering the biology of Mycobacterium tuberculosis from the complete genome sequence. *Nature.* 1998 Jun 11;393(6685):537-44. Erratum in: Nature 1998 Nov 12;396(6707):190.

Cunningham AF, Spreadbury CL. Mycobacterial stationary phase induced by low oxygen tension: cell wall thickening and localization of the 16-kilodalton alpha-crystallin homolog. *J Bacteriol.* 1998 Feb;180(4):801-8.

da Fonseca DP, Frerichs J, Singh M, Snippe H, Verheul AF. Induction of antibody and T-cell responses by immunization with ISCOMS containing the 38-kilodalton protein of Mycobacterium tuberculosis. *Vaccine.* 2000 Aug 15;19(1):122-31.

Demissie A, Leyten EM, Abebe M, Wassie L, Aseffa A, Abate G, et al; VACSEL Study Group. Recognition of stage-specific mycobacterial antigens differentiates between acute and latent infections with Mycobacterium tuberculosis. *Clin Vaccine Immunol.* 2006 Feb;13(2):179-86.

Espitia C, Elinos M, Hernández-Pando R, Mancilla R. Phosphate starvation enhances expression of the immunodominant 38-kilodalton protein antigen of Mycobacterium tuberculosis: demonstration by immunogold electron microscopy. *Infect Immun.* 1992 Jul;60(7):2998-3001.

Fine PE. The BCG story: lessons from the past and implications for the future. *Rev Infect Dis.* 1989 Mar-Apr;11 Suppl 2:S353-9.

Gil O, Vilaplana C, Guirado E, Díaz J, Cáceres N, Singh M, Cardona PJ. Enhanced gamma interferon responses of mouse spleen cells following immunotherapy for tuberculosis relapse. *Clin Vaccine Immunol.* 2008 Nov;15(11):1742-4. Epub 2008 Sep 30.

Gruppo V, Orme IM. Dose of BCG does not influence the efficient generation of protective immunity in mice challenged with Mycobacterium tuberculosis. *Tuberculosis (Edinb).* 2002;82(6):267-73.

Guirado E, Amat I, Gil O, Díaz J, Arcos V, Caceres N, Ausina V, Cardona PJ. Passive serum therapy with polyclonal antibodies against Mycobacterium tuberculosis protects against post-chemotherapy relapse of tuberculosis infection in SCID mice. *Microbes Infect.* 2006 Apr;8(5):1252-9. Epub 2006 Jan 27.

Guirado E, Gil O, Cáceres N, Singh M, Vilaplana C, Cardona PJ. Induction of a specific strong polyantigenic cellular immune response after short-term chemotherapy controls bacillary reactivation in murine and guinea pig experimental models of tuberculosis. *Clin Vaccine Immunol.* 2008 Aug;15(8):1229-37. Epub 2008 Jun 4.

Lundberg 2006. TB Vaccines. Vienna April 2006.

Holten-Andersen L, Doherty TM, Korsholm KS, Andersen P. Combination of the cationic surfactant dimethyl dioctadecyl ammonium bromide and synthetic mycobacterial cord factor as an efficient adjuvant for tuberculosis subunit vaccines. *Infect Immun.* 2004 Mar;72(3):1608-17.

McCune RM, Feldmann FM, Lambert HP, McDermott W. Microbial persistence. I. The capacity of tubercle bacilli to survive sterilization in mouse tissues. *J Exp Med.* 1966 Mar 1;123(3):445-68.

Muñoz-Elías EJ, Timm J, Botha T, Chan WT, Gomez JE, McKinney JD. Replication dynamics of Mycobacterium tuberculosis in chronically infected mice. *Infect Immun.* 2005 Jan;73(1):546-51.

Olsen AW, Brandt L, Agger EM, van Pinxteren LA, Andersen P. The influence of remaining live BCG organisms in vaccinated mice on the maintenance of immunity to tuberculosis. *Scand J Immunol.* 2004 Sep;60(3):273-7.

Orme IM. Current progress in tuberculosis vaccine development. *Vaccine.* 2005 Mar 18;23(17-18):2105-8.

Orme M. The latent tuberculosis bacillus (I'll let you know if I ever meet one). *Int J Tuberc Lung Dis.* 2001 Jul;5(7):589-93.

Silva CL, Bonato VL, Coelho-Castelo AA, De Souza AO, Santos SA, Lima KM, et al. Immunotherapy with plasmid DNA encoding mycobacterial hsp65 in association with chemotherapy is a more rapid and efficient form of treatment for tuberculosis in mice. *Gene Ther.* 2005 Feb;12(3):281-7.

Vilaplana C et al. Double-blind randomized, placebo-controlled Phase I Clinical Trial of the therapeutical antituberculous vaccine RUTI®. *Vaccine.* 2009 (Submitted).

Wallace JG. The heat resistance of tubercle bacilli in the lungs of infected mice. *Am Rev Respir Dis.* 1961 Jun;83:866-71.

Wayne LG. Dormancy of Mycobacterium tuberculosis and latency of disease. *Eur J Clin Microbiol Infect Dis.* 1994 Nov;13(11):908-14.

World Health Organization. Global Tuberculosis Control: Surveillance, Planning, Financing WHO Report 2004. Geneva, Switzerland.

17. APPENDICES

17.1 CDC Classification System for HIV Infection

The CDC categorization of HIV/AIDS is based on the lowest documented CD4 cell count (Table 1) and on previously diagnosed HIV-related conditions (Tables 2 and 3). For example, if a patient had a condition that once met the criteria for Category B but now is asymptomatic, the patient would remain in Category B. Additionally, categorization is based on specific conditions, as indicated below. Patients in categories A3, B3, and C1-C3 are considered to have AIDS.

Table 1. CDC Classification System for HIV-Infected Adults and Adolescents

CD4 Cell Categories	Clinical Categories		
	**A**<br>**Asymptomatic, Acute HIV, or PGL**	**B**<br>**Symptomatic Conditions,#* not A or C**	**C**<br>**AIDS-Indicator Conditions***
**(1) ≥500 cells/µL**	A1	B1	C1
**(2) 200-499 cells/µL**	A2	B2	C2
**(3) <200 cells/µL**	A3	B3	C3

Key to abbreviations: CDC = U.S. Centers for Disease Control and Prevention; PGL = persistent generalized lymphadenopathy.
# For symptomatic conditions, see Table 2 .

* For AIDS-indicator conditions, see Table 3 .

Table 2. CDC Classification System: Category B Symptomatic Conditions

Category B symptomatic conditions are defined as symptomatic conditions occurring in an HIV-infected adolescent or adult that meet at least 1 of the following criteria:

a) They are attributed to HIV infection or indicate a defect in cell-mediated immunity.

b) They are considered to have a clinical course or management that is complicated by HIV infection.

Examples include, but are not limited to, the following:

- Bacillary angiomatosis
- Oropharyngeal candidiasis (thrush)
- Vulvovaginal candidiasis, persistent or resistant
- Pelvic inflammatory disease (PID)
- Cervical dysplasia (moderate or severe)/cervical carcinoma in situ
- Hairy leukoplakia, oral

- Idiopathic thrombocytopenic purpura
- Constitutional symptoms, such as fever (>38.5°C) or diarrhea lasting >1 month
- Peripheral neuropathy
- Herpes zoster (shingles), involving ≥2 episodes or ≥1 dermatome

Table 3. CDC Classification System: Category C AIDS-Indicator Conditions

Bacterial pneumonia, recurrent (≥2 episodes in 12 months)
Candidiasis of the bronchi, trachea, or lungs
Candidiasis, esophageal
Cervical carcinoma, invasive, confirmed by biopsy
Coccidioidomycosis, disseminated or extrapulmonary
Cryptococcosis, extrapulmonary
Cryptosporidiosis, chronic intestinal (>1-month duration)
Cytomegalovirus disease (other than liver, spleen, or nodes)
Encephalopathy, HIV-related
Herpes simplex: chronic ulcers (>1-month duration), or bronchitis, pneumonitis, or esophagitis
Histoplasmosis, disseminated or extrapulmonary
Isosporiasis, chronic intestinal (>1-month duration)
Kaposi sarcoma
Lymphoma, Burkitt, immunoblastic, or primary central nervous system
Mycobacterium avium complex (MAC) or *M kansasii* , disseminated or extrapulmonary
Mycobacterium tuberculosis , pulmonary or extrapulmonary
Mycobacterium , other species or unidentified species, disseminated or extrapulmonary
Pneumocystis jiroveci (formerly *carinii*) pneumonia (PCP)
Progressive multifocal leukoencephalopathy (PML)
Salmonella septicemia, recurrent (nontyphoid)
Toxoplasmosis of brain
Wasting syndrome due to HIV (involuntary weight loss >10% of baseline body weight) associated with either chronic diarrhea (≥2 loose stools per day ≥1 month) or chronic weakness and documented fever ≥1 month

REFERENCE:

Centers for Disease Control and Prevention. *1993 revised classification system for HIV infection and expanded surveillance case definition for AIDS among adolescents and adults* . MMWR Recomm Rep. 1992 Dec 18;41(RR-17):1-19. Available online at www.cdc.gov/mmwr/preview/mmwrhtml/00018871.htm.

17.2 Alcohol Intake Questions

How much alcohol do you drink in an average week?

1.	Beer/Cider	xxx	(n >= 0) cans/week
		xxx	(n >= 0) pints/week
		xxx	(n >= 0) sakiya (cartons)/week
		xxx	(n >= 0) quarts/week
2.	Whiskey	xxx	(n >= 0) tots/week
		xxx	(n >= 0) nips/week
		xxx	(n >= 0) half-jacks/week
3.	Brandy	xxx	(n >= 0) tots/week
		xxx	(n >= 0) nips/week
		xxx	(n >= 0) half-jacks/week
4.	Wine	xxx	(n >= 0) glasses/week

5.* Other, please specify name of drink and number of units in an average week (optional) A30

Name of drink xxx (n >= 0) units/week

6. On how many days in an average week would you drink alcohol? x (0 =< n <= 7) days/week

7.* Number of units of beer per week

8.* Number of units of whiskey per week

9.* Number of units of brandy per week

10.* Number of units of wine per week

11.* Total number of units of alcohol per week
